# Biosynthetic mechanisms of isoflavone accumulation affected by different growth patterns in *Astragalus mongholicus* products

**DOI:** 10.1186/s12870-022-03769-5

**Published:** 2022-08-23

**Authors:** Fusheng Zhang, Xuan Zhang, Yangyang Luo, Huijuan Li, Xuemei Qin

**Affiliations:** Modern Research Center for Traditional Chinese Medicine, the Key Laboratory of Chemical Biology and Molecular Engineering of Ministry of Education, Shanxi University, Taiyuan, 030006 Shanxi China

**Keywords:** *Astragalus mongholicus*, Isoflavone accumulation, Biosynthesis, Transcriptomics, Metabolomics, Different growth patterns

## Abstract

**Background:**

At present, *Astragalus mongholicus* products on the market represent two growth patterns: imitative wild *A. mongholicus* (WAM) and cultivated *A. mongholicus* (CAM)*.* The 6-year-old WAM (A6) and 2-year-old CAM (B2) products are often sold as commodities. This study aimed to explore the effects of the abovementioned growth patterns on the biosynthetic mechanisms of isoflavone accumulation in *A. mongholicus* products.

**Results:**

In this paper, the content of calycosin-7-*O*-β-D-glucoside in 6-year-old WAM (A6) was significantly higher than that in 2-year-old CAM (B2) based on high-performance liquid chromatography. Tissue anatomy indicated that A6 has developed phloem fibers, thickened secondary walls, and a more well-developed vascular system than B2. Thirteen differentially accumulated metabolites were found in A6 and B2 by UHPLC-ESI-Q-TOF-MS/MS, of which isoflavones were highly and significantly enriched in A6. By combining transcriptomics and metabolomics analysis, we found that the metabolomics profile was the same as the transcriptomics profile in both A6 and B2. In total, 11 novel isoflavone-related genes were isolated using BLAST and functional annotation through RNA-Seq and Iso-Seq. The results of integrated analysis, Short Time-series Expression Miner analysis, and Pearson correlation analysis showed that the regulation of four key enzymes, cinnamate 4-hydroxylase, 6-deoxychalcone synthase, chalcone reductase, and chalcone isomerase, led to the high accumulation of isoflavones in A6. In addition, *Am*UFGT (c778119) and *Am*UCGT (c303354) were predicted to be 7-*O*-glycosyltransferases by phylogenetic analysis; these genes catalyze formononetin and calycosin, respectively.

**Conclusions:**

The findings of this work will clarify the differences in the biosynthetic mechanism of isoflavone accumulation between A6 and B2, which will guide the cultivation of *A. mongholicus*.

**Supplementary Information:**

The online version contains supplementary material available at 10.1186/s12870-022-03769-5.

## Introduction

Astragali radix is origin from the dried root of *Astragalus membranaceus* (Fisch.) Bge. or *Astragalus membranaceus* (Fisch.) Bge. var. *mongholicus* (Bge.) Hsiao, which belongs to the legume family [1]. Astragali radix is one of the most commonly used traditional Chinese herbs and has been used for debility, chronic illness, and spleen deficiency [2]. In addition, the dried roots of the plant have been added to decoctions to prevent diseases and maintain health in daily life [3]. With the modernization of traditional Chinese medicine, Astragali radix has been listed on the “One Root of Medicine and Food” by the National Health Commission of the People’s Republic of China [4]. At present, *A. mongholicus* occupies the majority of the market for Astragali radix, and the authentic production area of *A. mongholicus* is Shanxi Province in China [5].

**Fig. 1:**
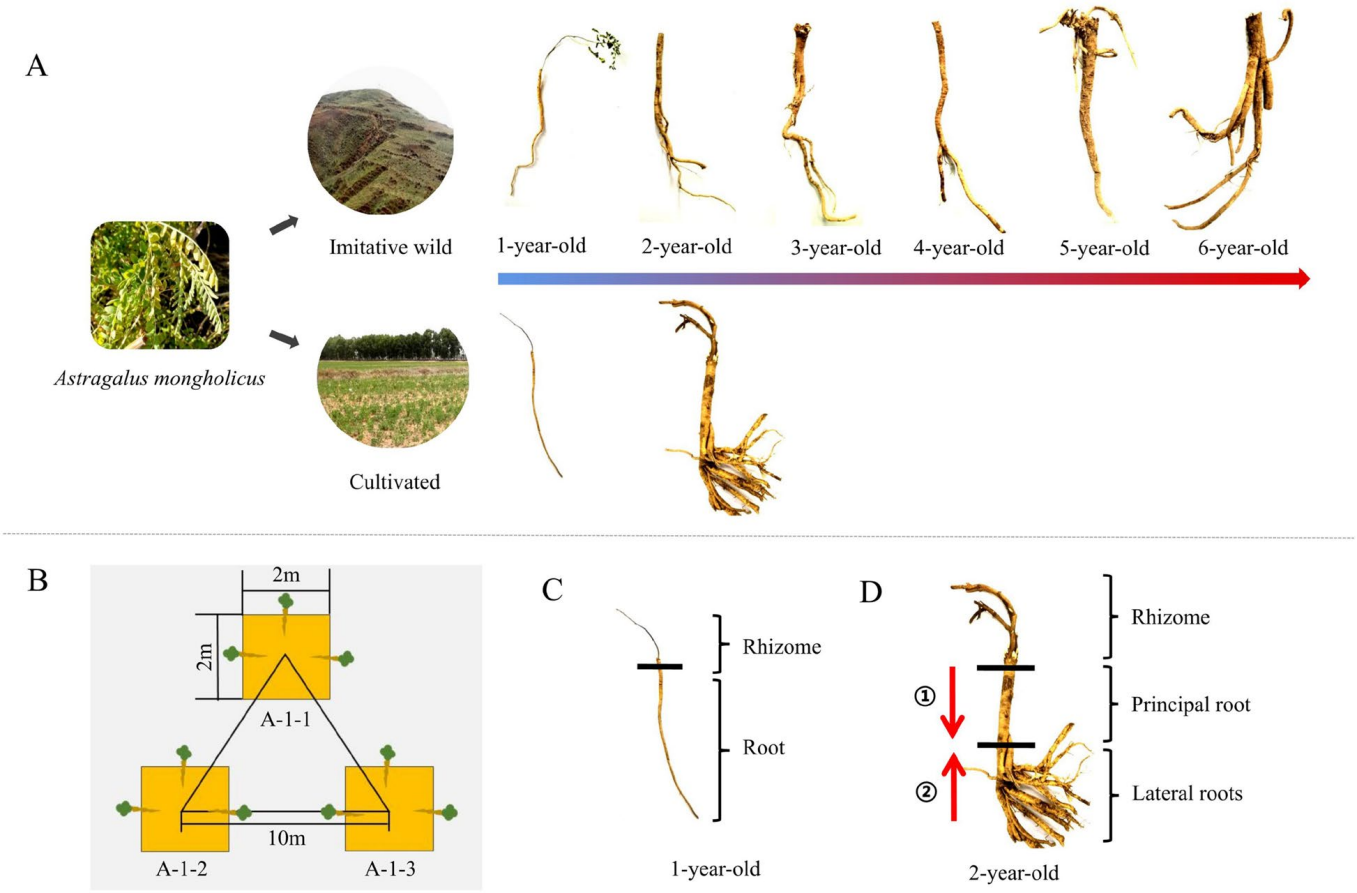
Schematic representation of the sample selection. **A** 1- to 6-year-old *A. mongholicus* with different growth patterns in their growth environment. **B** Selection of quadrats and collection of samples. In the figure, A: imitative wild *A. mongholicus*, A-1: the number represents growth in years, A-1-1: the first quadrat of A-1. **C**, **D** Different treatments of 1- to 6-year-old *A. mongholicus*. In the figure, ①/②: the samples were collected from *A. mongholicus* in accordance with the direction of the red arrow

*A. mongholicus* is a cross-pollinated plant that is self-incompatible [6]. By the 1980s, the resources for the wild *A. mongholicus* and imitative wild *A. mongholicus* (WAM) were in short supply, which led to the emergence of the much needed cultivated *A. mongholicus* (CAM) [7]. Although both products belong to *A. mongholicus*, their growth patterns are different. The planting of WAM is mainly through artificially sowing the seeds on semisunny slopes, letting the plants grow naturally. Until the harvest of WAM, no manual management is taken during the growth period. Additionally, the semisunny slopes were ploughed and the weeds were removed before sowing seeds, which is beneficial for the growth of WAM [7]. The roots of WAM grow vertically, and the length of the root can reach up to 1 m [7]. WAM is primarily distributed in the middle of the Hengshan Mountains (Shanxi, China), with an altitude of 1300–1800 m. These plants primarily grow on semisunny slopes, whose soil is sandy loam with strong physical weathering, coarser particles, and much gravel. The thickness of the soil layer is more than 5 m. The growth period of WAM is at least 4 years but can reach 6 or more years, including beyond a dozen years. In contrast, CAM is primarily planted on farmland with sandy loam where the soil is loose with a thickness of approximately 60–90 cm (Fig. 1A) [8]. The seeds of CAM are mainly collected from wild *A. mongholicus* and/or WAM and need to be manually manipulated, including seeding, watering, and fertilizing [7]. Normally, CAM primarily grows for 2 years but cannot exceed 3 years. If the growth period exceeds 3 years, the rhizome of CAM will become rotten and/or hollow [9]. Additionally, the lateral roots of CAM will be significantly thickened, while the principal roots will grow slowly. These attributes will affect the continuous improvement of yield per mu. Most roots of CAM grow laterally, and they can be harvested after transplantation for a year [7, 8]. The 6-year-old WAM (A6) and 2-year-old CAM (B2) products are often sold as commodities on the Chinese medicine market, and B2 accounts for over 80% of the total transaction volume in commercial medicinal materials. On the other hand, the diameters of the principal roots are similar across A6 and B2, whereas A2 will be immature in terms of its length and diameter. Our study aimed to clarify the differences between the two products and explore the changes in isoflavones in WAM and CAM during the growth period (Fig. 1A).

Given the different cultivation patterns, some different physical characteristics have also emerged in WAM and CAM, such as the length and diameter of the principal roots and the intensity of the beany smell [10]. In addition, some studies have shown that WAM and CAM have different pharmacological effects, such as antifatigue effects [11, 12]. With regard to chemical content, the contents of formononetin and calycosin in WAM were higher than those in CAM [13, 14]. Moreover, the contents of ononin, calycosin-7-*O*-β-D-glucoside (CG), and astragaloside III in WAM were significantly higher than those in CAM [13, 14]. Furthermore, flavonoids such as CG and ononin in *A. mongholicus* increase with growth years [14]. Previous research has focused on the difference between traditional medicinal pharmacological effects and metabolites in WAM and CAM. However, the biosynthetic mechanisms of isoflavone accumulation in WAM and CAM still need to be clarified.

**Fig. 2:**
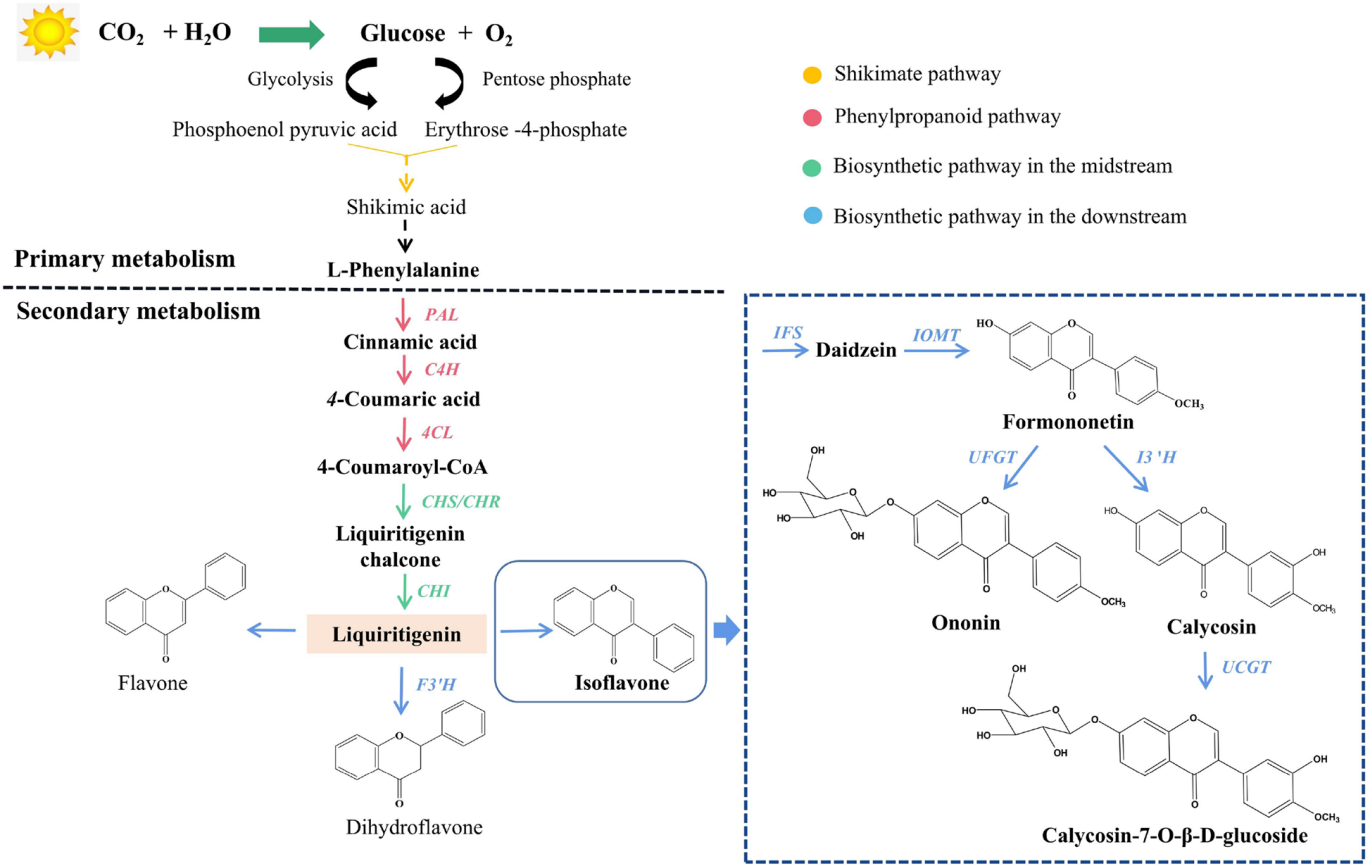
Isoflavone biosynthetic pathway in *A. mongholicus.* PAL, phenylalanine ammonia lyase; C4H, cinnamate-4-hydroxylase; 4CL, 4-coumaroyl CoA ligase; CHS, chalcone synthase; CHR, chalcone reductase; CHI, chalcone isomerase; IFS, isoflavone synthase; IOMT, isoflavone *O*-methyltransferase; I3′H, isoflavone 3′hydroxylase; UFGT, UDP-glucose: formononetin-7-*O*-glucosyltransferase; and UCGT, UDP-glucose: calycosin-7-*O*-glucosyltransferase

In living organisms, particularly in plants, algae, and certain bacteria, carbon dioxide (CO_2_) and water (H_2_O) are converted into glucose and oxygen (O_2_) through photosynthesis (Fig. 2). In plants, phosphoenol pyruvic acid (PEP) and erythrose-4-phosphate (E4P) are generated by the glycolysis and pentose phosphate pathways [15]. PEP and E4P are converted into shikimic acid and the phenylpropanine skeleton (C6-C3) primarily through the enzymatic reactions of the shikimic acid pathway [16, 17]. In general, the biosynthetic pathway of isoflavones starts from L-phenylalanine. Subsequently, 4-coumaroyl CoA is generated through continuous catalysis of phenylalanine ammonia-lyase (PAL), cinnamate 4-hydroxylase (C4H), and 4-coumaric acid coenzyme A ligase (4CL), which is based on L-phenylalanine. The abovementioned three steps are also called the phenylpropanoid pathway. After the formation of 4-coumaroyl CoA, 6-deoxychalcone synthase (CHS) or chalcone reductase (CHR) catalyzes the synthesis of liquiritigenin chalcone, and then liquiritigenin is generated by chalcone isomerase (CHI) [18, 19]. Liquiritigenin is often used as a precursor in subsequent reactions to form flavonoids, such as flavones, dihydroflavones, and isoflavones. Among them, dihydroflavones are mainly catalyzed by flavonoid 3′-hydroxylase (F3′H) [20, 21]. In *A. mongholicus*, isoflavone synthase (IFS) converts liquiritigenin into daidzein, and then formononetin is generated under the catalysis of isoflavone *O*-methyltransferase (IOMT). Calycosin is synthesized by isoflavone 3′hydroxylase (I3′H) with formononetin as a substrate [2, 22]. Finally, glycosylation modification occurs at the last step of the isoflavone biosynthetic pathway, during which UDP-glucuronosyltransferase (UGT) transfers glycosyl to the isoflavone receptor [23–25]. For *A. mongholicus*, formononetin and calycosin are glycosylated by UFGT and UCGT to produce ononin and CG, respectively.

To date, studies on WAM and CAM have been limited to their chemical components, such as isoflavones and astragalosides, while the abovementioned biosynthetic mechanism has not been explored. Metabolomics and transcriptomics reflect the process of information passing from genes to metabolites in plants from various aspects [26]. Metabolomics was used to explore whether the metabolomic differences in different cultivation patterns of *A. mongholicus* were consistent with the change trend of relevant genes. In contrast, transcriptomics was used to verify whether the expression of relevant genes was consistent with the abovementioned change trend of metabolites.

Thus, we focus on two main commercial products of *A. mongholicus* (A6 and B2). First, the content of CG between WAM and CAM was determined by high-performance liquid chromatography (HPLC). Second, tissue anatomy was used to compare the structural characteristics of various vegetative organs between WAM and CAM. To explore the differences in isoflavone compounds in *A. mongholicus* with two different growth patterns, Iso-seq and RNA-seq were used for transcriptomic sequencing, and UHPLC-ESI-Q-TOF-MS/MS was used for metabolomics. Moreover, quantitative real-time polymerase chain reaction (qRT–PCR) was conducted to validate the conclusions drawn from transcriptomics. Finally, integrated analysis, Pearson correlation, and Short Time-series Expression Miner (STEM) analysis of the gene expression and metabolite data were also used to provide a comprehensive understanding of the biosynthetic mechanisms between WAM and CAM. The findings of this work will clarify the biosynthetic mechanism of isoflavone accumulation between A6 and B2, which will guide the cultivation of *A. mongholicus*.

## Materials and Methods

### Plant materials and treatments

The fresh roots of WAM (1 to 6 years) and CAM (1 to 2 years) were collected from Shanxi Province, China. The information for all samples is listed in Table S1. All samples were identified as *A. mongholicus* by Professor Xue-mei Qin and preserved in the Modern Research Center for Traditional Chinese Medicine, Shanxi University (China). To reflect production practice and the overall pattern of isoflavones in *A. mongholicus*, two batches of samples were collected from the same area. The first batch of samples (No.20180628) was collected on June 28 (2018), for differential analysis of the overall profile of the transcriptomics and metabolomics between WAM and CAM. Moreover, to further verify the accuracy of the above data and results, another batch of samples (No.20181023) was collected on October 23, 2018.

For the samples collected from each growth year, three quadrats were selected as three biological replicates, and the length and width of each quadrat were 2 m. The distance between each quadrat was 10 m. Three plants were selected from each quadrat to reduce the difference within the group (Fig. 1B).

The roots of A1 and B1 were selected for follow-up tests; the rhizomes were removed to reflect the overall situation of the root sample (Fig. 1C). For A2-A6 and B2, the lengths of the principal root of *A. mongholicus* are between 30 cm-70 cm [27]. The diameter of the base (principal root under rhizome) is thicker, whose growth age is the same as the plant, while the tip (end of the principal root) is smaller, whose growth age is younger. The tip of the root has been grown just for the recent 1-2 years. In order to truly reflect the growth years of one sample, the base and tip from *A. mongholicus* were cut out separately, then mixed as the final experimental sample of a single plant for subsequent related experiments (The detailed steps of sample processing were in Fig. S1 ).

### Analysis of CG by HPLC

#### Diameter of A. mongholicus with different growth patterns

The root diameter data were displayed (or plotted) using a frequency distribution diagram in order to minimize within year variability [28]. Currently, the medicinal material samples have relatively large within-group variance regarding the diameter of root samples from a single growth year. The purpose of this method is to select medicinal material samples that are more representative of the growth years to ensure the credibility of the results.

**Fig. 3:**
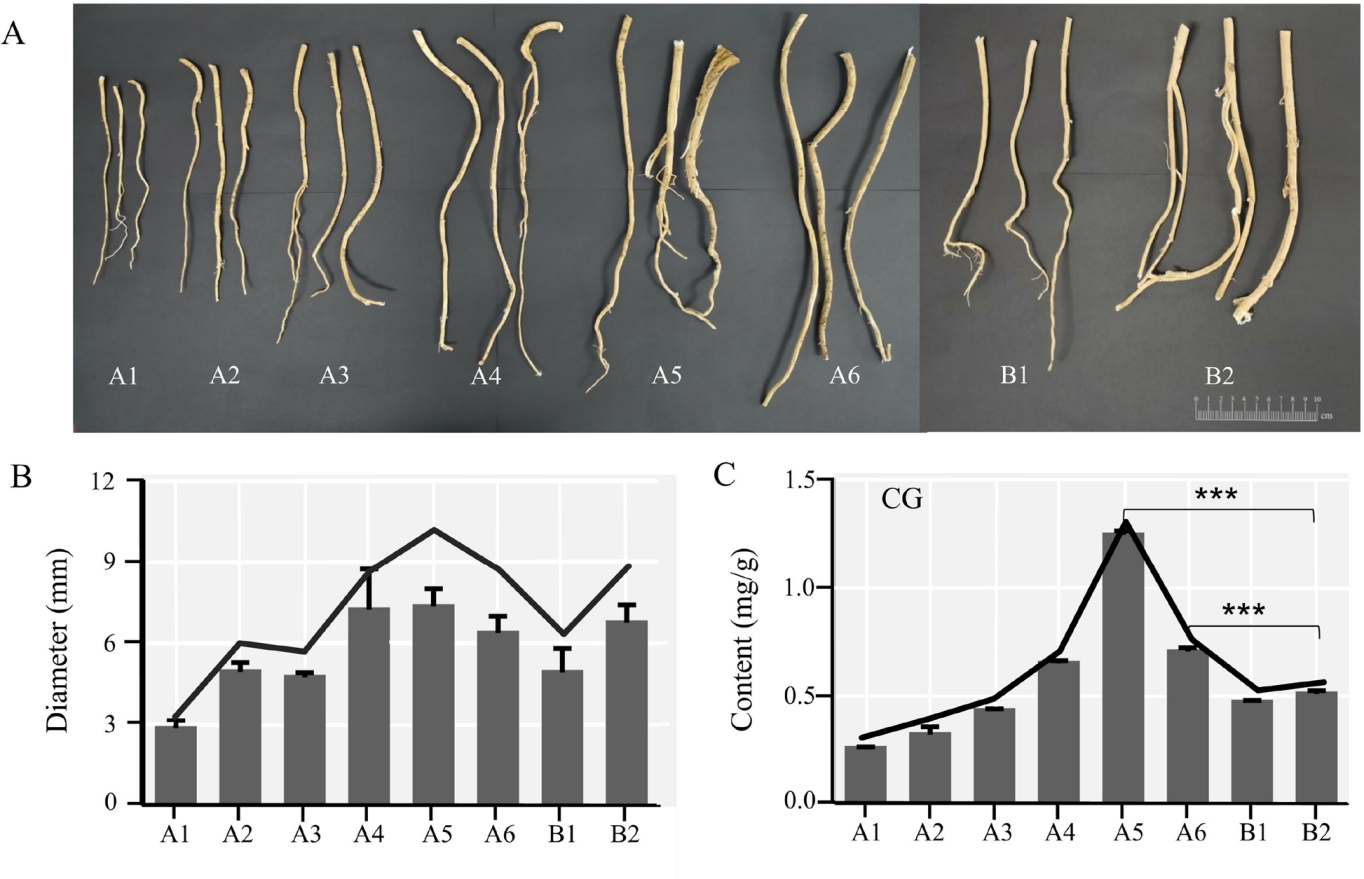
Distribution of CG in *A. mongholicus*. **A** Samples used for HPLC. **B** The diameter of *A. mongholicus*. **C** The content of CG in *A. mongholicus* by HPLC. In the figure, A: imitative wild *A. mongholicus*, B: cultivated *A. mongholicus*; A1: the number represents growth in years, B1: the number represents growth in years

First, the rhizome and redundant lateral roots of *A. mongholicus* were cut and removed, and diameters at sections 3.5 cm below the incision were recorded (Fig. 3A). Next, the scatter diagrams and frequency distribution diagrams were analyzed by SPSS 16.0 (International Business Machines Co., Ltd., USA) to assess the range of diameters for WAM and CAM across different growth years. The range of diameters was calculated from the frequency distribution histogram, where the values covered in 60% of the whole frequency distribution were retained. Samples with diameters in the above-mentioned diameter range, were taken in for subsequent experiments.

#### Reference solution

The standard substance CG was precisely weighed, and methanol was added to make a 50 μg/mL reference solution [1].

#### Test solution

Approximately 1 g of powder (particle size ≤ 250±9.9 μm) was weighed and placed into a round-bottomed flask. A total of 50 mL of methanol was added to the round-bottomed flask, and the round-bottomed flask was weighed. The powder was extracted into the reflux device for 4 h. After extraction, the round-bottomed flask was weighed again after cooling, and methanol was added to the round-bottomed flask to replenish the lost weight. Then, 25 mL of the subsequent filtrate was measured precisely, and the filtrate was recovered until it was completely dry. Finally, the residue was dissolved in methanol, and the test solution was transferred to a volumetric flask (5 mL) [1].

#### Chromatographic conditions

Venusil MP C18 column (250 mm × 4.6 mm, 5 µm). The mobile phase consisted of acetonitrile (A)-0.2% formic acid in water (B), the conditions were set as follows: 0–20min, 20–40%A; 20–30 min, 40% A. The detection wavelength is 260nm.

### Principal roots anatomy of *A. mongholicus*

The principal roots of WAM (1 to 6 years old) and CAM (1 to 2 years old) were cut into suitable sizes. All samples were fixed in FAA for more than 24 hours. After dehydration, transparent wax, and embedding, the samples were sliced by a microtome (LEICA RM2265) to a thickness of 8 μm. Then, the sections were stained with safranin-fast green and sealed with neutral balsam. Finally, the paraffin sections were observed and photographed by a microscope (LEICA DM2500).

### Metabolite extraction and analysis

#### Sample preparation

A total of 1 g of powder was steeped in a 15-fold volume of 75% methanol in a reflux device and extracted twice; each extraction lasted for 1 h. Then, the test solution was filtered through a 0.22 μm Millipore filter. Finally, 3 μL of test solution was injected for UHPLC-ESI-Q-TOF-MS/MS analysis [29].

#### Chromatographic conditions

An ACQUITY™ UPLC HSS T3 column (100 mm × 2.1 mm, 1.8 μm, Waters Corporation, USA) was used for separation at 35 °C. The mobile phase consisted of 0.1% formic acid in water (A) and 0.1% formic acid in acetonitrile (B). The conditions were set as follows: 0.01–3 min, 1%–10% B; 3–9 min, 10%–30% B; 9–18 min, 30%–100% B; 18–22 min, 100%–100% B; 22–24 min, and 100%–0.01% B. The flow rate was maintained at 0.4 mL/min, and the injection volume was set to 3 μL. On this basis, all samples were set to the same volume and mixed as quality control (QC) samples [29].

#### Mass spectrometry conditions

In positive mode (POS), the ion spray voltage floating (ISVF) was set to 5500 V; the ESI heater temperature was maintained at 600 °C; the nebulizer gas (GS 1), auxiliary gas (GS 2), and curtain gas (CUR) were set to 55, 55, and 30 psi, respectively; and the declustering potential (DP) and collision energy (CE) were set to 100 and 10 V, respectively. The accumulation time of TOFMS was set to 0.15 s. In negative mode (NEG), the ISVF was set to −4500 V, and the other parameters were the same as those in POS. During the positive and negative acquisition modes, MS and MS/MS data were acquired simultaneously via an information-dependent acquisition (IDA) mode. Under the IDA-MS/MS experiment, CE was set to 40±20 V, and at most 10 candidate ions were monitored per cycle. In addition, the dynamic background subtract mode was used, and continuous calibration was performed every four injections during analysis [29].

#### Metabolomics data processing

Metabolomics data were transformed through One-MAP-PTO 2.0. The metabolites with more than 50% missing values in the group were removed to ensure parallelism between samples and metabolites, and the data were normalized by mass. Second, the data were imported into SIMCA-P 14.1 (Umetrics, Sweden) for pattern recognition after Pareto scaling, and multidimensional statistical analysis was performed. On this basis, the metabolites with VIP > 1 and *P* < 0.05 were screened and identified as differentially accumulated metabolites (DAMs).

### Combined analysis of Iso-Seq and RNA-Seq for nonreference transcriptomics

#### Construction of the nonreference transcriptomics database

The next-generation data files were bam files. First, the reads of interest (ROI) were obtained by in-hole correction of the next-generation data. Then, the ROI were classified on the basis of whether the 5′primer/3′primer/Poly A was complete and chimeric. The full-length nonchimeric sequence was obtained. Finally, the full-length nonchimeric sequences were clustered to obtain nonredundant isoforms.

Subreads were extracted and filtered from raw data (RNA-Seq), and adapters and low-quality sequences were removed. The filtered reads were compared with the isoform sequence. Besides, the clean reads that were not comparable to the isoform were spliced using Trinity software to obtain transcripts, and then subsequent analysis was performed.

The nonredundant isoform and spliced transcripts were combined and clustered by CD-HIT, and unigenes were obtained and used for subsequent analysis. Transcriptomic sequencing, including cDNA construction, was undertaken by Shanghai Personal Biotechnology Co., Ltd. (China).

#### Identification of genes related to the isoflavone biosynthetic pathway

The full names of candidate enzymes in *A. mongholicus* were searched in the nucleotide library of the National Center for Biotechnology Information (NCBI), and all sequences with full-length coding sequences (CDSs) and corresponding GenBank IDs were recorded. Moreover, the names of candidate enzymes were searched via the functional annotation data. Then, short candidate sequences were removed, and the remaining sequences were blasted through Nucleotide BLAST in NCBI. Afterward, the sequences with high query cover and percent identity were retained. Finally, the abovementioned sequences were compared with the CDSs published on NCBI by multiple sequence alignment in DNAMAN (Lynnon Biosoft Co., Ltd., USA).

#### qRT–PCR of isoflavone-related genes

Fresh roots of *A. mongholicus* were used for RNA extraction. Purity and concentration were measured via 1.5% agarose gel electrophoresis using a SpectraMax® QuickDrop™ spectrophotometer (Molecular Devices, LLC, USA). cDNA was amplified from the total RNA (500 ng) by qRT–PCR. The reaction system was 20 μL, including 10 μL of SYBR Premix Ex Taq II 2× (Takara Biomedical Technology (Beijing) Co., Ltd., China), 0.8 μL of Primer F (10 μM), 0.8 μL of Primer R (10 μM), 2 μL of cDNA, and 6.4 μL of ddH_2_O. All reactions were conducted in 96-well plates using the Heal Force Real-Time PCR System (Heal Force CG-05, Hangzhou Jingle Scientific Instrument Co., Ltd., China). The qPCR protocol included annealing at 95 °C for 60 s, followed by 40 cycles of 95 °C for 15 s, primer annealing at 58 °C for 15 s, and extension at 72 °C for 45 s. A negative control without template for each primer pair was included in each qPCR analysis. Three biological replicates were used for each sample. The expression levels of genes were calculated using 2^−△△Ct^ [30]. The qRT–PCR primers are shown in Table S2. The sequences of candidate genes are in Additional file 2.

### Statistical and phylogenetic analysis

#### Statistical analysis

Statistical analysis was conducted using SPSS 16.0. Correlations among data were calculated using Pearson’s correlation coefficients (r).

#### Phylogenetic analysis

The amino acid sequences of UGTs were collected from NCBI (http://www.ncbi.nlm.nih.gov). The information for these sequences is listed in Table S3. Sequence alignment was performed by ClustalW analysis. Evolutionary distances were computed using the overall mean distance in MEGA7.0 (https://www.megasoftware.net). For phylogenetic analysis, an unrooted neighbor-joining (NJ) tree was generated with MEGA7.0. Bootstrap values from 1000 replicates are indicated at each branch [31].

## Results

### Differences in the CG content across *A. mongholicus* with different growth patterns

CG is a main active component of *A. mongholicus* and is an isoflavone. A scatter diagram and frequency distribution diagram were used to visualize the range of diameters of WAM and CAM across different growth years (Fig. 3A, Fig. S2/S3). Based on the diameters of *A. mongholicus* with different growth patterns, the diameters of WAM increased over time, with the diameter of A5 being the maximum. The change in the diameter of CAM also increased over time. The diameters of A6 and B2 were the same (Fig. 3B, Table S4). Similarly, the CG contents of A5 were the highest across all samples. The CG contents of A5 and A6 were significantly higher than that of B2 (Fig. 3C). Moreover, the Pearson correlation between the diameters and CG content in *A. mongholicus* with different growth patterns was significant (r = 0.83, *P* = 0.01). Therefore, the change in the CG content was consistent with the change in the diameter of *A. mongholicus*.

### Anatomical characteristics of principal roots of *A. mongholicus*

**Fig. 4:**
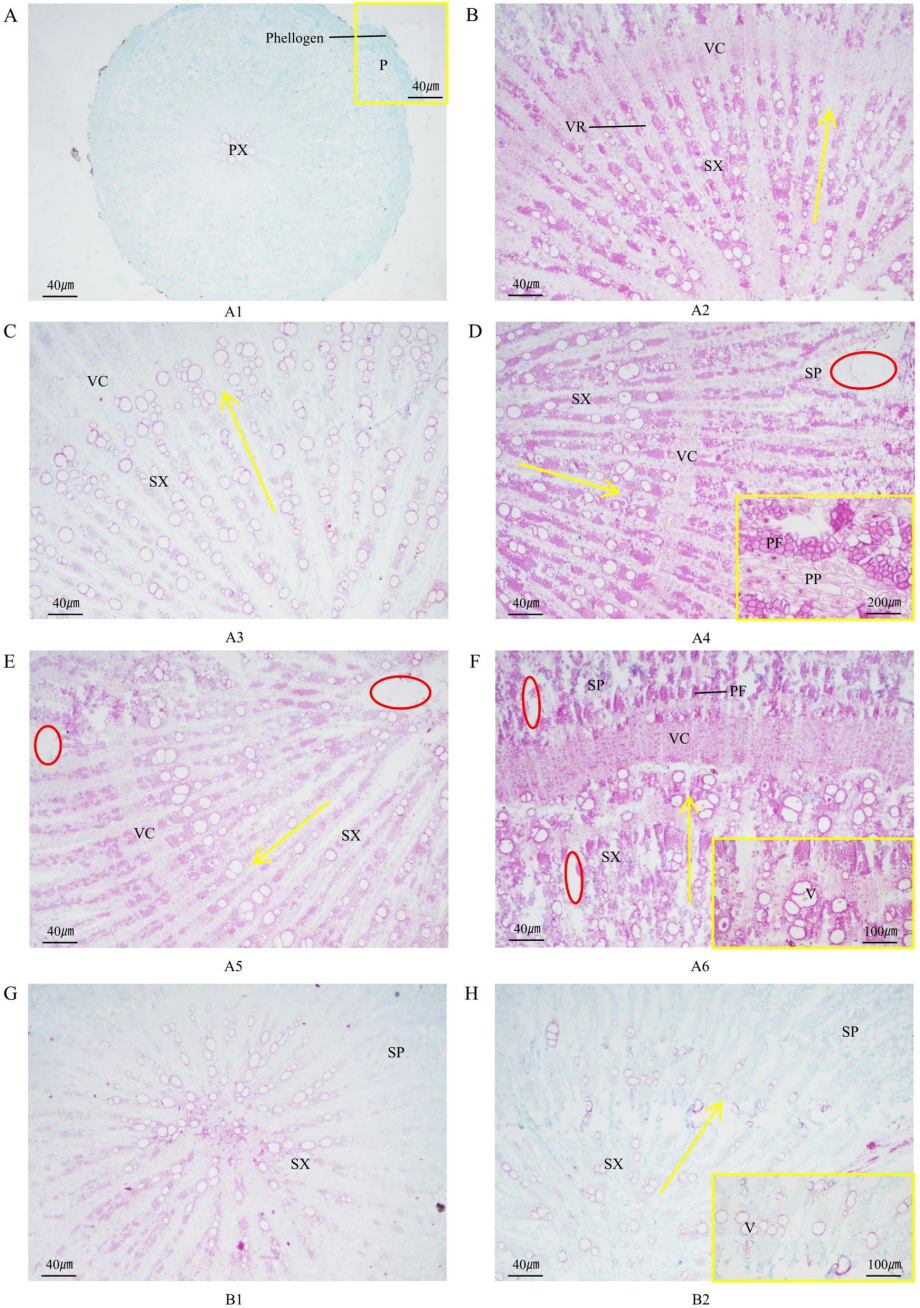
Structural anatomy of *A. mongholicus* roots. P: Periderm; SP: Secondary phloem; PF: Phloem fiber; PP: Phloem parenchyma cells; PX: Primary xylem; V: Vessel; VR: Vascular ray; VC: Vascular cambium; and SX: Secondary xylem. The red circle indicates the gaps in the principal roots. The yellow box indicates an enlarged view of the principal root

The principal roots of *A. mongholicus* were composed of periderm and secondary vascular tissues (Fig. 4A/B). The periderm of the roots consisted of the cork layer, phellogen, and phelloderm in sequence from the outside to the inside. The cells of the cork layer were arranged neatly and tightly in the radial direction, and some cells of the cork layer were crushed (Fig. 4A). The secondary vascular tissue consisted of secondary phloem, vascular cambium, and secondary xylem. The secondary phloem was mainly composed of phloem parenchyma and phloem fibers. The phloem parenchyma was darkly stained, and the contents of the cells were rich in spindle-shaped organs (Fig. 4D). However, the vascular rays in the main root consisted of 1-2 rows of cells (Fig. 4B). The phloem fibers were combined into bundles, while the sieve tubes and companion cells only appeared in the secondary phloem near the vascular cambium. The vascular cambium was arranged in a ring with 5-7 layers of phloem parenchyma cells, which was the dividing line of the secondary xylem and secondary phloem (Fig. 4D).

Only the primary xylem and periderm were found in the principal roots of A1, and secondary vascular tissues began to appear in the principal roots of A2 (Fig. 4A/B). The vessels were measured according to the direction of the yellow arrow, which means the number of vessels per ray. A2 had approximately 10 vessels per ray, A3-A4 had approximately 20 vessels per ray, and A5-A6 had approximately 15 vessels per ray (Fig. 4B/C/D/E/F). Abundant vascular tissue could enable the transportation and distribution of water, minerals, and organic nutrients in the plants. The vessels in the principal roots of A3-A4 were the highest, which indicated that in this period, WAM grew vigorously, while the growth of A5-A6 became slow. Gaps began to appear in the vascular rays of the principal roots in A4-A6, and the parenchyma cells in the rays were tightly arranged. However, the thickness of the slice may not have been sufficient, thus resulting in gaps during the slicing process.

In addition to primary xylem, B1 also had secondary xylem. The proportion of secondary phloem in B2 increased, and the number of vessels in the secondary xylem also increased (Fig. 4G/H). Compared with B2, the phloem fibers of the secondary phloem in A6 were well developed, with a significantly thickened secondary wall (Fig. 4F/H). Moreover, the vascular cambium in the principal roots of A6 was more obvious and had well-developed and tightly arranged vascular bundles with ring-shaped connections. There were more vessels with a larger diameter in the principal roots of A6, while B2 had only approximately 10 vessels (Fig. 4F).

### Metabolomics analysis of *A. mongholicus* with different growth patterns

#### Principal component analysis

**Fig. 5:**
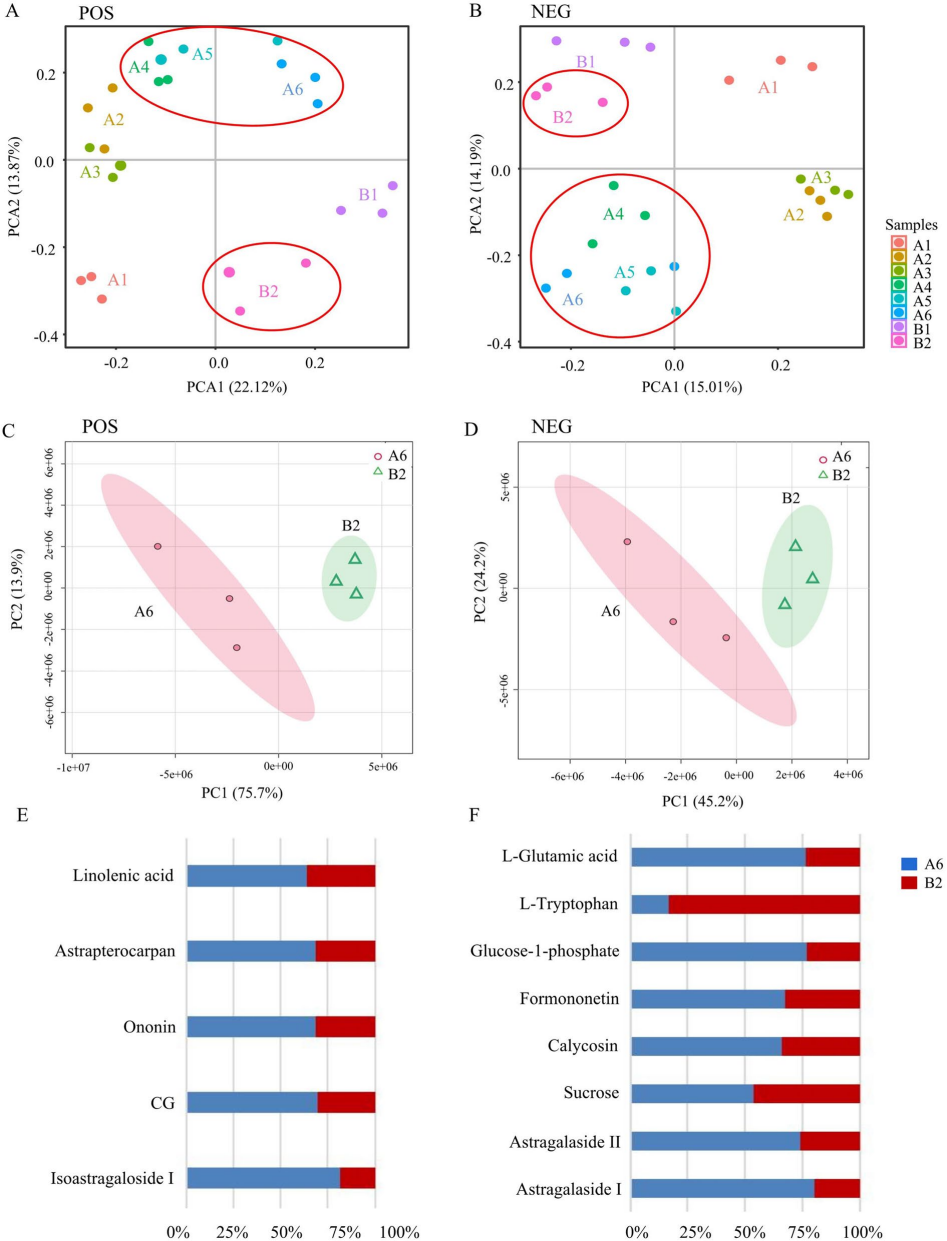
PCA of metabolomics data for *A. mongholicu*s with different growth patterns based on UHPLC-ESI-Q-TOF-MS/MS. The data were obtained from the area of each component based on LC–MS. **A** PCA of metabolomics in POS. **B** PCA of metabolomics in NEG. **C** PCA of A6 and B2 in POS. **D** PCA of A6 and B2 in NEG. **E** DAMs of A6 and B2 in POS. **F** DAMs of A6 and B2 in NEG

Principal component analysis (PCA) was used for the metabolomics data analysis. PCA is an unsupervised pattern recognition method that can effectively determine important information in data by reducing the original complexity of that data [32]. In this study, the metabolomics of *A. mongholicus* with different growth patterns were analyzed. From the level of metabolomics, A6 and A4-A5 were distinguished by the PC1 axis in POS, whereas B1-B2 and A4-A6 were distinguished by the PC2 axis. For A4 and A6, the aggregation of A5 and A6 was more evident than that of A4 (Fig. 5A). However, the PCA results for A6 and B2 showed that A6 and B2 were distinguished by the PC1 axis in POS (Fig. 5C). In NEG, B1-B2 and A4-A6 were distinguished by the PC2 axis (Fig. 5B). Consistent with the previous PCA results in POS, A6 and B2 were distinguished by the PC1 axis in NEG (Fig. 5D).

In summary, it was found that A6 and B2 were significantly divided into two groups, whether in POS or NEG. The results also revealed that the influence of different growth patterns on *A. mongholicus* was reflected in the metabolites. Hence, DAMs should be further analyzed to explore the effects of the abovementioned growth patterns on the metabolites in *A. mongholicus*.

#### Identification of differentially accumulated metabolites

The partial least squares discriminant analysis (PLS-DA) model was verified by the permutation test (Fig. S4A/B). In the figure, the R^2^ and Q^2^ values on the left were lower than those on the right, and the regression line of Q^2^ intersected at the negative ordinate axis. Significant differences in the metabolites were observed between A6 and B2. Thus, the OPLS-DA model was used to obtain the scatter plot of A6 and B2 (Fig. S4C/D).

A total of 13 DAMs were identified from A6 and B2 by comparing the m/z values, retention time, and fragmentation patterns using the standards in the database (Fig. 5E/F). The DAMs included five primary metabolites and eight secondary metabolites. Of the five primary metabolites, L-tryptophan was significantly higher in B2 than in A6. Other primary metabolites, such as L-glutamic acid, glucose-1-phosphate, linolenic acid, and sucrose, were significantly higher in A6 than in B2. Additionally, saponins and flavonoids including astragalaside II, astragalaside I, isoastragaloside I, and astrapterocarpan, were significantly higher in A6 than in B2. Consistent with the HPLC results, isoflavones, including formononetin, ononin, calycosin, and CG, accumulated more in A6 (Fig. 5E/F, Table S5).

#### Classification of isoflavones in A. mongholicus from metabolomics data

**Fig. 6:**
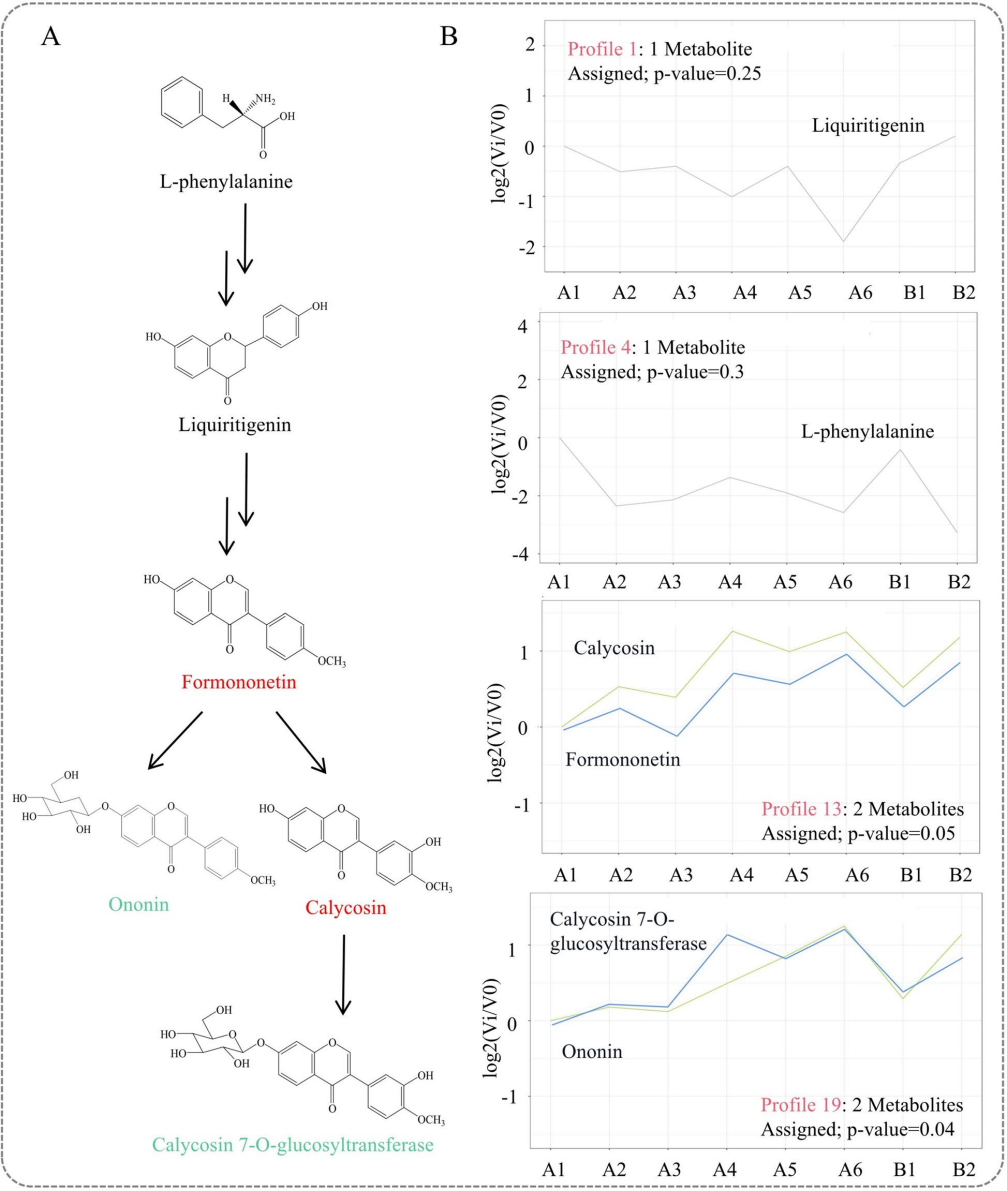
STEM analysis of metabolites in the isoflavone biosynthetic pathway by UHPLC-ESI-Q-TOF-MS/MS. The results were analyzed by STEM online at http://www.omicshare.com/. **A** Isoflavone biosynthetic pathway. The same color indicates that the profiles of metabolites were the same. Black indicates that the metabolite had a different profile. **B** The change trend of metabolites in different profiles

A series of clustering tests were used to classify the different metabolites into different profiles through STEM analysis to reveal the unique change patterns of biological samples. The most representative metabolite cluster and corresponding trend characteristics were found. Based on the metabolomics data, a total of six metabolites in the isoflavone biosynthetic pathway were identified, including L-phenylalanine, liquiritigenin, and four isoflavones in the downstream pathway from formononetin to CG (Fig. 6A, Table S5).

The peaks of six metabolites in the isoflavone biosynthetic pathway were analyzed by STEM (Table S6). The results showed that the six metabolites corresponded to four profiles. Among them, profile 19 was significantly enriched in two metabolites (*P* < 0.05). The enrichment of the other three profiles, 13, 1, and 4, was not significant.

Profile 13 included formononetin and calycosin, which showed alternating increasing and decreasing trends for A1-A6 and B1-B2. Moreover, profile 19 included CG and ononin, which gradually increased in A1-A6 and B1-B2 and were significantly enriched in A6. L-phenylalanine and liquiritigenin corresponded to profile 4 and profile 1, respectively. Profile 4, which contained L-phenylalanine, was characterized by a gradual decrease in A1-A6 and B1-B2. Profile 4 showed that liquiritigenin was enriched in B2 (Fig. 6B).

### Nonreference transcriptomics analysis by Iso-Seq and RNA-Seq

**Fig. 7:**
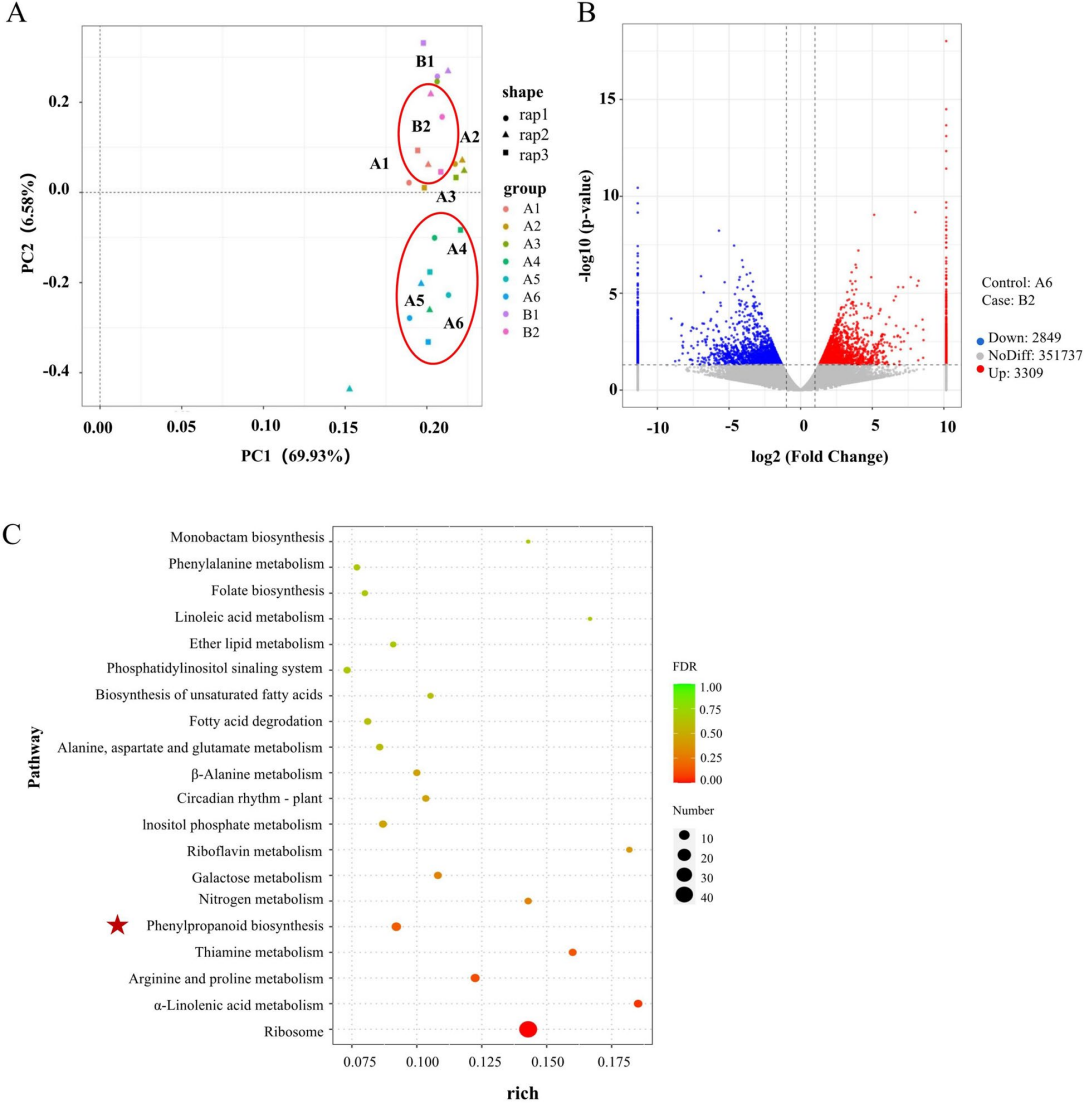
Differential expression analysis of *A. mongholicu*s with different growth patterns by Iso-Seq and RNA-Seq, via PacBio and Illumina HiSeq, respectively. **A** PCA of transcriptomics for *A. mongholicu*s with different growth patterns. **B** The volcano plot of DEGs in A6 and B2. **C** The enriched KEGG pathways for DEGs in A6 and B2. The five-point star indicates the phenylpropanoid pathway

At the transcriptomics level, when PC2 was plotted along the X axis, A4-A6 and B2 were divided into two groups (Fig. 7A). The results further revealed that the transcriptomic profile of *A. mongholicus* with different growth patterns was basically consistent with the trends identified for the metabolite profiles (Fig. 4A).

#### Functional annotation of unigenes

In total, 833,216 unigenes were obtained with an N50 of 978 bp. The unigenes were annotated by Gene Ontology (GO) and Kyoto Encyclopedia of Genes and Genomes (KEGG). GO analysis categorized 27,252 transcripts into molecular functions, cellular components, and biological processes. Genes in the molecular function category were enriched for transferase activity, kinase activity, and protein kinase activity. Genes involved in cellular components were primarily enriched for the membrane, membrane part, and integral component of the membrane. With regard to biological processes, the genes were primarily involved in the regulation of biological, metabolic, and cellular processes (Fig. S5). The KEGG results demonstrated that 4,276 transcripts were mapped to 325 KEGG pathways. A total of 89 genes were assigned to the biosynthesis of secondary metabolites (Fig. S6).

#### Identification of phenylpropanoid pathways and isoflavone-related genes

Gene expression among different groups was analyzed by DESeq analysis. A total of 6158 differentially expressed genes (DEGs) were found between A6 and B2, including 3309 upregulated genes and 2849 downregulated genes (Fig. 7B, Additional file 3). All DEGs between A6 and B2 were annotated against the KEGG database. The results showed that the DEGs were primarily involved in the ribosome, phenylpropanoid pathway, arginine and proline metabolism, α-linolenic acid metabolism, and thiamine metabolism. The ribosome, arginine and proline metabolism, and α-linolenic acid pathways were also related to translation, amino acid metabolism, and lipid metabolism, respectively. Thiamine metabolism is rooted in the metabolism of cofactors and vitamins. In addition, the phenylpropanoid pathway is related to the biosynthesis of secondary metabolites. Notably, the isoflavone biosynthetic pathway is an important branch of the phenylpropanoid pathway (Fig. 7C) [33]. Therefore, the differences in isoflavones between A6 and B2 were primarily due to the differential expression of isoflavone-related genes.

In this study, 11 candidate genes participating in the isoflavone synthesis pathway were found, including c759450 (*PAL,* GenBank: ON420729), c831948 (*C4H,* GenBank: MN241505), c795398 (*4CL*, GenBank: ON420730), c780996 (*CHS,* GenBank: MN241506), c828977 (*CHR,* GenBank: ON420728), c801189 (*CHI,* GenBank: MN241507), c759107 (*IFS,* GenBank: ON420726), c773593 (*IOMT,* GenBank: MN241507), c792702 (*I3′H,* GenBank: ON420727), c778119 (*UFGT,* GenBank: ON375915), and c303354 (*UCGT,* GenBank: MN241498) (Table S7/S8). All candidate genes had been submitted in NCBI database.

### Expression patterns of isoflavone-related genes in *A. mongholicu*s

#### Expression analysis of 11 isoflavone-related genes in A. mongholicus

Based on the clustering results of the transcriptomics samples, A4, A5, and A6 were clustered together. B2/B1/A1 and A2/A3 were also clustered together. Additionally, it was found that the differences among the three quadrats of A2-A6 were large, whereas the differences among the three quadrats of A1, B1, and B2 were small.

**Fig. 8:**
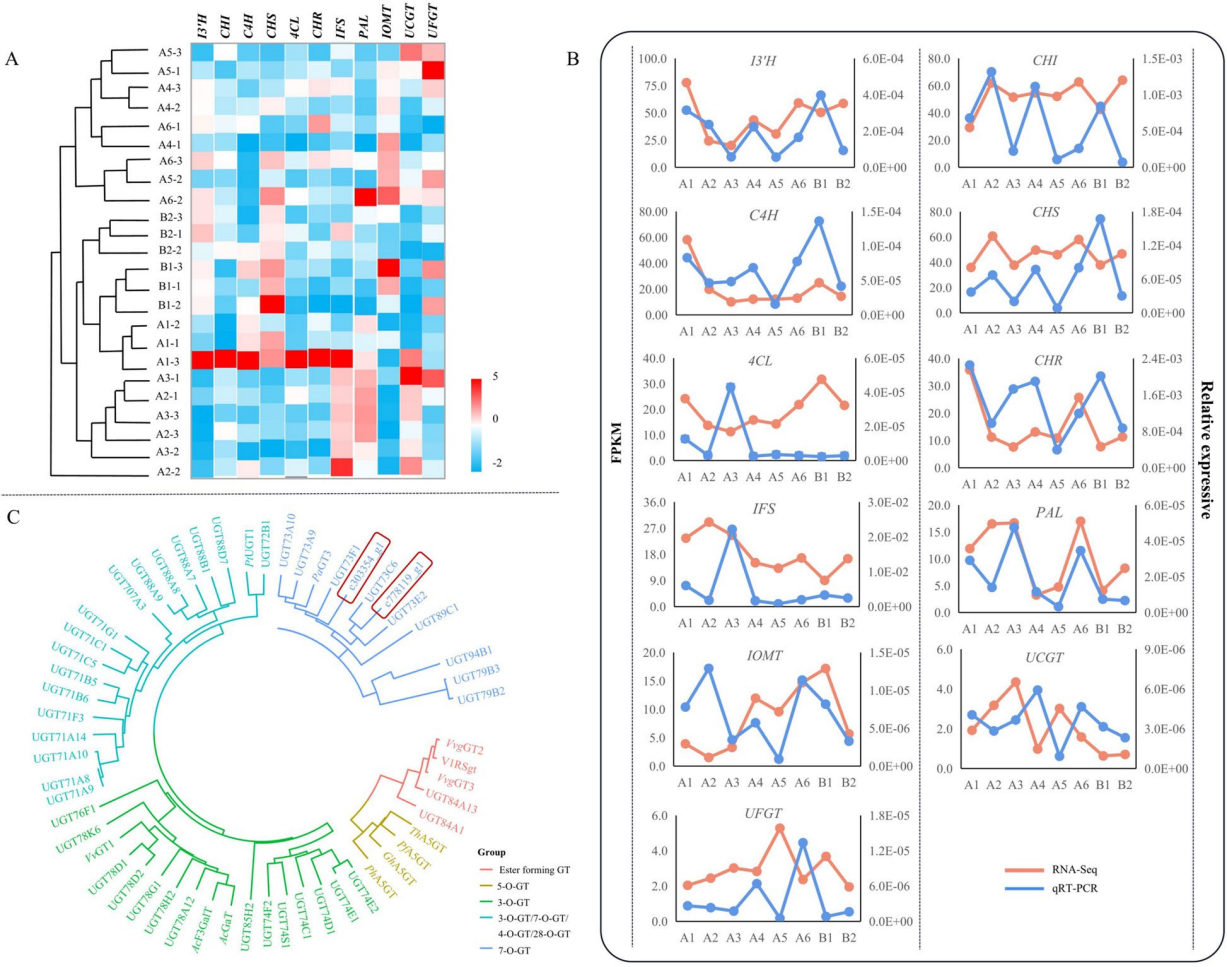
Expression analysis of isoflavone-related genes. **A** Heatmap of FPKM for isoflavone-related genes and clustering of samples. **B** The integrated analysis of qRT–PCR and RNA-Seq validation for 11 isoflavone-related genes in *A. mongholicus.* **C** Phylogenetic analysis of UGTs. The red boxes represent two candidate genes

The expression levels of key enzymatic genes in the isoflavone biosynthetic pathway of *A. mongholicus* were predicted based on FPKM values. The expression levels of *I3′H*, *CHI*, *C4H*, *CHS*, *4CL*, and *CHR* were high in all samples, whereas those of *IFS*, *PAL*, *IOMT*, *UFGT*, and *UCGT* were low in all samples. Overall, the total expression of the 11 isoflavone-related genes in A1 was the highest across all samples, and the expression levels of all genes in A6 were higher than those in B2. In addition, the expression levels of *C4H*, *CHS*, and *CHI* were the highest in A1, and no significant change was observed in the other groups. The expression levels of *I3′H*, *CHR*, and *C4H* in A6 were second only to those in A1, and the expression level of *PAL* was highest in A6. The results showed that the expression level of *IOMT* was the highest in B1, and the expression levels of *CHS*, *CHI*, and *IFS* were the highest in A2 (Fig. 8A). Except that, the STEM analysis about the qRT-PCR of the 11 isoflavone-related genes were put in the Additional file 1 (Fig. S7).

#### Integrated analysis of 11 isoflavone-related genes with RNA-Seq and qRT–PCR

The results of qRT–PCR and RNA-Seq were selected for integrated analysis (Fig. 8B/S8). The results of the Pearson correlation analysis of *PAL* and *I3′H* using qRT–PCR and RNA-Seq data showed a correlation coefficient (r) of 0.738/0.599, which further indicated that the expression profiles based on qRT–PCR were consistent with those based on RNA-Seq. The expression of *CHS* and *CHI* in B1 differed between the qRT–PCR and RNA-Seq analyses. The qRT–PCR and RNA-Seq results of *C4H* in A3 were different. Beyond that, the expression of *CHR* and *4CL* based on qRT–PCR was inconsistent with that based on RNA-Seq in both A3 and B1. For *IFS* and *IOMT*, the expression based on qRT–PCR in A2 and B1 was different from that based on RNA-Seq. Other genes with low expression, such as *UFGT* and *UCGT*, showed remarkable differences across all samples between the qRT–PCR and RNA-Seq results (Fig. 8B/S8). The differences between the qRT–PCR and RNA-Seq results were primarily concentrated in A2, A3, and B1. Considering that the samples used for qRT–PCR and RNA-Seq analyses were collected at different times, the expression patterns of *PAL* and *I3′H* in *A. mongholicus* were relatively stable.

Based on the qRT–PCR results, the expression levels of *IFS*, *CHR*, *CHI*, *CHS*, and *I3′H* were high in all samples, whereas those of *4CL*, *C4H*, *PAL*, *IOMT*, *UFGT*, and *UCGT* were low in all samples. Moreover, the expression levels of *IFS* and *4CL* were the highest in A3, and no significant differences were found in the other samples. The expression levels of *C4H* and *CHS* were the highest in B1. However, the expression levels of *CHR*, *CHI*, *UCGT*, *4CL*, *I3′H*, and *IOMT* in A1 were higher than those in B1 (Fig. S9). Consistent with the FPKM results, the overall expression levels of 11 isoflavone-related genes were higher in A1 than in B1, and the expression levels of all genes in A6 were also higher than those in B2. Furthermore, the expression levels of 11 isoflavone-related genes in A6 and B2 were compared separately. The results showed that the expression levels of isoflavone-related genes in A6 were higher than those in B2, except for *IFS*. Among them, the expression level of *CHI* in A6 was significantly higher than that in B2 (Fig. S10).

#### Phylogenetic analysis of AmUCGT and AmUFGT

Given the identification of two UGTs with low expression in *A. mongholicus*, UGTs were assessed by phylogenetic analysis and were located downstream of the isoflavone biosynthetic pathway. The bootstrapped NJ phylogenetic tree was constructed on the basis of *Am*UCGT (c303354), *Am*UFGT (c778119), and 54 other UGTs (Table S3) whose functions were already known. These UGT enzymes had five functions: ester-forming GT, 5-O-GT, 3-O-GT, 7-O-GT, and 3-O-GT/7-O-GT/4-O-GT/28-O-GT. c303354 of *A. mongholicus* was clustered with UGT73E2 and UGT73C6, whose functions were 7-O-GT. Similarly, c778119 was clustered with UGT73F1 (Fig. 8C). Considering that UGT73E2, UGT73F1, and UGT73C6 are flavonol-3-*O*-glycoside-7-*O*-glucosyltransferases, c303354 and c778119 were predicted to be 7-*O*-glycosyltransferases that catalyze formononetin and calycosin, respectively.

#### Correlation analysis and biosynthetic mechanisms of isoflavone-related genes and metabolites

**Fig. 9:**
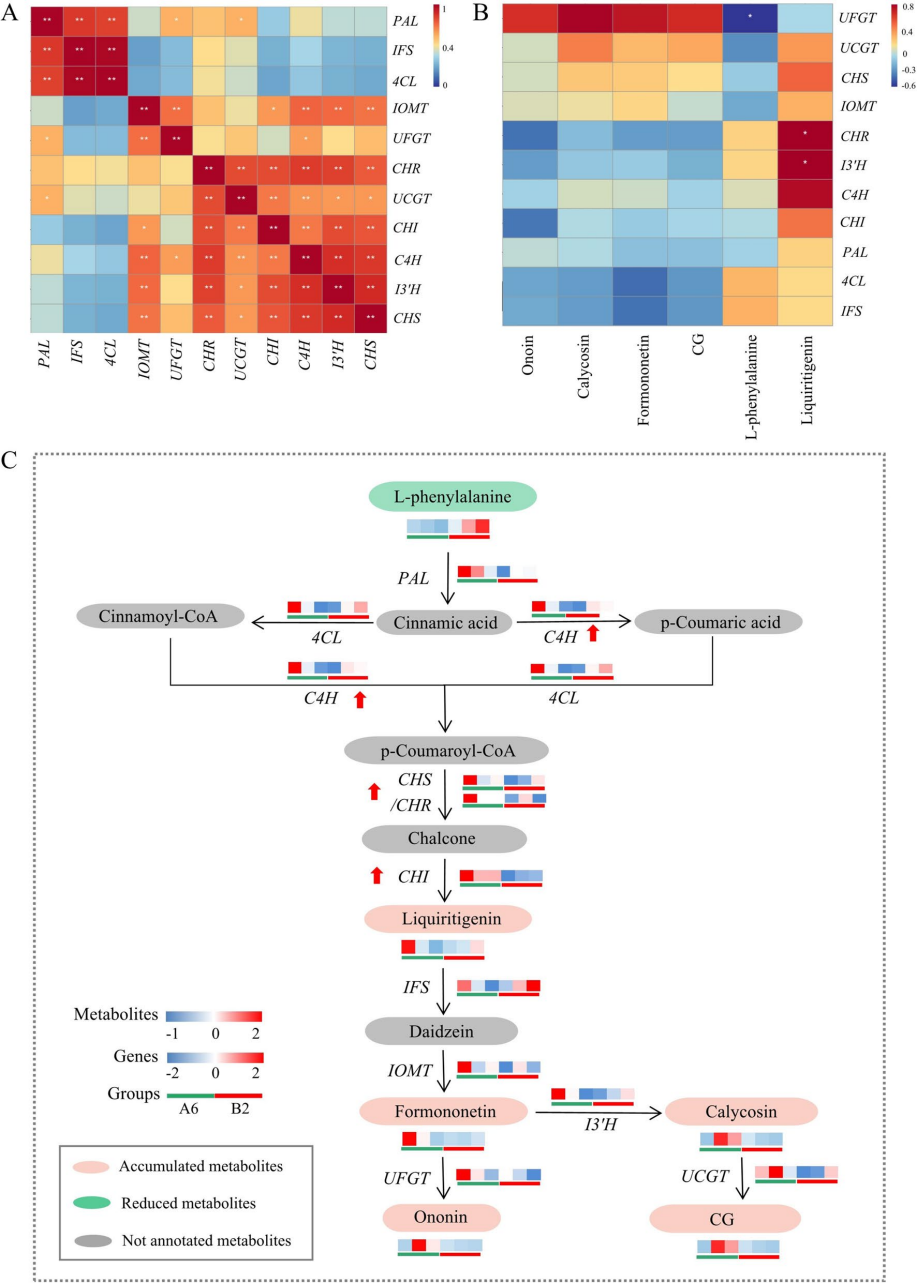
Correlation analysis and biosynthetic mechanisms of isoflavone-related genes and metabolites. **A** Pearson correlation analysis of 11 isoflavone-related genes by qRT–PCR. *P<0.05, **P<0.01. Correlation analysis was conducted by using an online tool (http://www.lc-bio.com/). **B** Pearson correlation analysis of 11 isoflavone-related genes and 6 metabolites by qRT–PCR and UHPLC-ESI-Q-TOF-MS/MS. **C** Expression profiles of genes and metabolites involved in the isoflavone biosynthetic pathway in A6 and B2

Pearson correlations of the expression levels of 11 isoflavone-related genes from qRT–PCR showed that *PAL* and *4CL* had a significant positive correlation with *IFS* (*P*<0.01), and *C4H* had a strong correlation with *I3′H*, *IOMT*, and *UCGT* (*P*<0.05). In addition, *CHR* and *CHI* had a positive correlation with *UCGT* (*P*<0.01) (Fig. 9A). Furthermore, *CHS* and *CHI* were strongly correlated with *IOMT, UCGT,* and *I3′H* (*P*<0.05) (Fig. 9A, Table S9). Moreover, the Pearson correlation between six metabolites and 11 genes across all samples showed that *CHR* and *I3′H* had a significant positive correlation with liquiritigenin (*P*<0.05) (Fig. 9B). *UFGT* had a significant negative correlation with L-phenylalanine (*P*<0.05) (Fig. 9B, Table S10). Hence, *CHS*, *CHI*, *CHR*, and *C4H* played an important role in the accumulation of isoflavones in the downstream pathway.

Transcriptomic and metabolomic analyses showed that the isoflavone biosynthetic pathway, particularly the phenylpropanoid pathway, plays an important role in the difference between A6 and B2. Based on these results, the accumulation mechanisms of isoflavones in A6 were primarily due to the high expression of the four key enzymes in the isoflavone biosynthetic pathway. L-phenylalanine was decreased, and liquiritigenin was produced, which led to the formation of the flavonoid backbone (C6-C3-C6). First, the high expression of *C4H* in the phenylpropane pathway led to a decrease in L-phenylalanine. Subsequently, the high expression of rate-limiting enzymes, such as CHI, CHS, and CHR, further promoted the accumulation of liquiritigenin in the midstream pathway. The downstream pathway focused on modifying the flavonoid backbone. Finally, isoflavones, including calycosin, formononetin, ononin, and CG, showed significant accumulation because of the high expression of *I3′H*, *IOMT*, *UFGT*, and *UCGT* (Fig. 9C).

## Discussion

In this study, samples of A1-A6 and B1-B2 were collected to explore the effects of different growth patterns and growth years on the chemical components of *A. mongholicus*. Although the diameters of A6 and B2 were relatively similar, the CG content in A6 was significantly higher than that in B2. Tissue anatomy also indicated that A6 has developed phloem fibers, thickened secondary walls, and a more well-developed vascular system than B2. Combined transcriptomics and metabolomics analysis showed that the metabolomics profile of WAM and CAM was consistent with the change in the transcriptomics profile. Further analysis of the DAMs showed that isoflavones such as formononetin, ononin, calycosin, and CG accumulated more in A6 than in B2. The results also indicated that the expression levels of 11 isoflavone-related genes in A6 were higher than those in B2, except for *IFS*. Finally, after comprehensive analysis of multiple methods, including an integrated analysis containing RNA-seq and qRT–PCR, Pearson correlation, and STEM analyses, the accumulation of isoflavones in A6 was primarily due to the high expression of the four key genes, including *C4H*, *CHS*, *CHI*, and *CHR*. In this study, *Am*UFGT (c778119) and *Am*UCGT (c303354) were also predicted to be 7-*O*-glycosyltransferases by phylogenetic analysis; these enzymes catalyze formononetin and calycosin, respectively.

Given its wide application in medicinal plants, metabolomics has been gradually applied to mechanistic research on the secondary metabolic pathways of these plants and the identification of traditional Chinese medicines, including *Panax ginseng* [34], *Bupleurum chinense* [35], *Saposhnikovia divaricata* [36], and *Scutellaria baicalensis* [37]. Gao et al. [38] identified 12 components in 2- to 6-year-old WAM by HPLC-UV-ELSD. PCA showed that A2-A3 and A4-A6 clustered into two groups, and within the second group, A4 was separated from the group containing A5 and A6. The results also suggested that eight isoflavones and four astragalosides led to differences in the groups containing the 2- to 6-year-old WAM samples. Our study clarified the differences between WAM and CAM across different growth years by UHPLC-ESI-Q-TOF-MS/MS. The PCA results in POS were consistent with those in previous studies [38]. Moreover, the differences among groups showed a gradually increasing trend with growth years, and the differences in metabolites between B2 and A4-A6 were the most evident.

Moreover, the DAMs in A6 and B2, including polysaccharides, saponins, amino acids, and isoflavones, were found through metabolomics analysis. We also found that the isoflavone content in A6 was significantly higher than that in B2. A study on WAM showed that the main factors affecting the content of flavonoids included altitude and latitude and that longitude also had a certain influence [39]. Therefore, this finding may be due to the high altitude and latitude of WAM, which stimulate WAM to produce a stress response and the accumulation of isoflavones. However, a study on tissue anatomy has indicated that the developed phloem fibers and thickened secondary walls are beneficial for blocking cold air. The well-developed vascular system of the roots is also more conducive to *A. mongholicus* absorbing water and mineral salt from arid environments [40]. This finding also further indicated that the well-developed secondary vascular tissues in A6 provide sufficient nutrients for the growth of *A. mongholicus*.

"orange is *Citrus reticulata* Blanco when it is born in Huainan, and it is *Citrus trifoliata* L. when born in Huaibei". This also shows that different growth environments could have different influences on the appearance of traits and accumulation of secondary metabolites in plants with the same origin. The reasons for this phenomenon are more complex and still need to be explored. In future experiments, we will combine the existing experimental results, try to use the same seeds of *A. mongholicus,* and let them grow under both growth patterns. We will also further explore the impact of altitude, temperature, nutrition, water, etc., on CAM and WAM.

Comprehensive analysis of transcriptomics and metabolomics is important for analyzing the internal changes in plants from both levels. Therefore, both techniques can provide a comprehensive understanding of the molecular functions and regulatory mechanisms in plants [41]. Most studies on the combination of metabolomics and transcriptomics use three biological replicates, and 3–10 plants at the same growth stage are usually mixed as a biological replicate [42–44]. Based on a survey, the planting areas of *A. mongholicus* in Hunyuan County (Shanxi, China) reached 18,666 hm^2^ in 2019 [45]. Previously, our research group found that the growth of the base and tip of *A. mongholicus* and the distribution positions of saponins and flavonoids on *A. mongholicus* differed across years [38]. As the design of experimental samples is important, we designed three quadrats of the same distance and size for *A. mongholicus* with the same growth years to select representative samples. In addition, we collected three plants in each quadrat and used different treatments for *A. mongholicus* at different growth stages.

Here, we found that A4-A6 clustered together at both the transcriptomic and metabolomic levels. A4-A6 and B2 were divided into two significantly different groups, which was consistent with previous results [38]. Similarly, the sample clustering results based on transcriptomics showed that A4-A6 and B2 clustered into two groups. However, we found that the differences among the three quadrats for A6 were greater than those for B2 based on the qRT–PCR results. Although the three biological replicates of A6 were weakly correlated in qRT–PCR results, we still found that the expression levels of isoflavone-related genes in A6 were higher than those in B2 based on qRT–PCR, and the expression level of *CHI* in A6 was significantly higher than that in B2.

The isoflavone biosynthetic pathway has attracted considerable attention in recent years as a branch of the phenylpropanoid pathway in legumes. Studies have found that the key enzymes in the isoflavone biosynthetic pathway include PAL, CHS, CHI, and IFS, which can regulate isoflavone biosynthesis at the pretranscriptional and posttranscriptional levels [46]. Moreover, with regard to plant disease resistance, CHS is the key rate-limiting enzyme for the synthesis of flavonoids and isoflavones [46]. Zhang et al. [47] pointed out that CHR is an essential enzyme in the synthesis of daidzein. The flavone branch of the phenylalanine pathway is an important pathway for flavone synthesis. C4H is the key enzyme in the second step of the phenylalanine pathway and is also considered a rate-limiting enzyme in this pathway [48]. Studies have shown that CHS and CHI constitute the rate-limiting enzymes for flavonoid biosynthesis [49–51]. A study on hairy roots of *A. membranaceus* examined methyl jasmonate (MJ) and UV radiation and showed that CHI and IFS are key enzymes in the MJ-induced regulation of the isoflavone biosynthetic pathway. Furthermore, PAL and C4H play an important role in the UV-B-induced regulation of isoflavone biosynthesis [52]. In our study, isoflavones such as formononetin, ononin, calycosin, and CG accumulated more in A6 than in B2 according to UHPLC-ESI-Q-TOF-MS/MS. Moreover, C4H, CHI, CHR, and CHS played an important regulatory role in *A. mongholicus* according to bioinformatics analysis, Pearson correlation analysis, and trend analysis. The high expression of these four enzymes further promoted the accumulation of isoflavones in A6.

The last step in the biosynthesis of isoflavones in plants is the glycation reaction, in which UGTs catalyze the substrates to form various glycosides [53]. The glycation reaction involves glycosyl donors and glycosyl receptors. Previous studies have shown that UGTs have substrate preferences. Flavonols, flavanones, isoflavones, and flavanols can be catalyzed by different UGTs to produce corresponding glycosides [54]. Glycosyl donors include UDP-glucose, UDP-galactose, UDP-rhamnose, and UDP-arabinose [55]. In this study, the functions of c303354 and c780119 were initially predicted as having a glycosyl transfer function of 7-O, but these functions were not verified. In the future, the functions of the two enzymes could be verified in *Escherichia coli* or *Saccharomyces cerevisiae*. Subsequently, RNA interference (RNAi) or overexpression of key enzymes, such as CHS, CHI, CHR, C4H, UCGT, and UFGT, could also be conducted in the transgenic receptor system of *A. mongholicus* to verify their functions and strengthen the relevant research foundation.

## Conclusions

Transcriptomic and metabolomic results have greatly clarified the similarities in the change trends of the metabolite profile and transcriptome profile of WAM and CAM. Isoflavones, including formononetin, onion, calycosin, and CG, were highly enriched in A6. Our analyses further indicate that the regulation of 4 key enzymes (C4H, CHS, CHI, and CHR) was the main reason for the high accumulation of isoflavones in A6.

## Supplementary Information

**Additional file 1: Table S1.** Information for all samples. **Table S2.** qRT–PCR primers used in this study. **Table S3.** Sequence information of UGTs used for phylogenetic analysis. **Table S4.** Diameter range of *A. mongholicus* under different growth modes. **Table S5.** Detailed information on the identified metabolites in *A. mongholicus*. **Table S6.** The peak area of metabolites in the isoflavone biosynthetic pathway. **Table S7.** Information on candidate genes. **Table S8.** FPKM of candidate genes. **Table S9.** Pearson correlation analysis of 11 isoflavone-related genes by qRT–PCR. **Table S10.** Pearson correlation analysis of 11 isoflavone-related genes and 6 metabolites. **Figure S1.** Schematic diagram of the sample processing method. **Figure S2.** Diameter scatter plot and frequency distribution histogram of *A. mongholicus*. **Figure S3.** Diameter scatter plot and frequency distribution histogram of *A. mongholicus*. **Figure S4.** Analysis of metabolomics for A6 and B2. **Figure S5.** Distribution of GO terms for all annotated unigenes in the biological process, cellular component, and molecular function categories. **Figure S6.** KEGG pathways enriched by unigenes. **Figure S7.** Expression of isoflavone-related genes in *A. mongholicus* by qRT–PCR analysis. **Figure S8.** Differential expression of isoflavone-related genes in A6 vs. B2 by qRT–PCR analysis. **Figure S9.** STEM analysis of isoflavone-related genes by qRT–PCR.

**Additional file 2.** The sequences of candidate genes.

**Additional file 3.** DESeq.A6_vs_B2.

## Data Availability

Data generated and analyzed during this study are included in the published article, its additional files and publicly available repositories.
