# Supplementary material for: Biosynthetic mechanisms of isoflavone accumulation affected by different growth patterns in Astragalus mongholicus products
Source: BMC Plant Biol. 2022 Aug 23;22:410. doi: 10.1186/s12870-022-03769-5 (PMC9396891; doi:10.1186/s12870-022-03769-5)
Supplement: Supplementary file 1 — Additional file 1: Table S1. Information for all samples. Table S2. qRT–PCR primers used in this study. Table S3. Sequence information of UGTs used for phylogenetic analysis. Table S4. Diameter range of A. mongholicus under different growth modes. Table S5. Detailed information on the identified metabolites in A. mongholicus. Table S6. The peak area of metabolites in the isoflavone biosynthetic pathway. Table S7. Information on candidate genes. Table S8. FPKM of candidate genes. Table S9. Pearson correlation analysis of 11 isoflavone-related genes by qRT–PCR. Table S10. Pearson correlation analysis of 11 isoflavone-related genes and 6 metabolites. Figure S1. Schematic diagram of the sample processing method. Figure S2. Diameter scatter plot and frequency distribution histogram of A. mongholicus. Figure S3. Diameter scatter plot and frequency distribution histogram of A. mongholicus. Figure S4. Analysis of metabolomics for A6 and B2. Figure S5. Distribution of GO terms for all annotated unigenes in the biological process, cellular component, and molecular function categories. Figure S6. KEGG pathways enriched by unigenes. Figure S7. Expression of isoflavone-related genes in A. mongholicus by qRT–PCR analysis. Figure S8. Differential expression of isoflavone-related genes in A6 vs. B2 by qRT–PCR analysis. Figure S9. STEM analysis of isoflavone-related genes by qRT–PCR. [file 12870_2022_3769_MOESM1_ESM.doc]

Table S1. Information for all samples

| Time and purpose | Sample | | Longitude | Latitude | Growth pattern | Growth year | Location |
| --- | --- | --- | --- | --- | --- | --- | --- |
| 2018.6.28-6.29  1. RNA-Seq and Iso-Seq 2.UHPLC-ESI-Q-TOF-MS/MS | A1 | A-1-1 | E：113°72′56.98″ | N：39°44′28.45″ | Wild | 14 months  (1 year old) | Youfanggou Village, Qianfoling Township, Hunyuan County, Datong City, Shanxi Province |
|  |  | A-1-2 | E：113°72′54.88″ | N：39°44′28.35″ | Wild |  |  |
|  |  | A-1-3 | E：113°72′53.90″ | N：39°44′27.05″ | Wild |  |  |
|  | A2 | A-2-1 | E：113°72′61.18″ | N：39°44′29.41″ | Wild | 26 months (2-year-old) | Kuanping Guizi Gitch, Qianfoling Township, Hunyuan County, Datong City, Shanxi Province |
|  |  | A-2-2 | E：113°72′58.81″ | N：39°44′30.36″ | Wild |  |  |
|  |  | A-2-3 | E：113°72′57.01″ | N：39°44′29.41″ | Wild |  |  |
|  | A3 | A-3-1 | E：113°72′92.56″ | N：39°44′38.56″ | Wild | 38 months (3-year-old) | Kuanping Dongpo Village,Qianfoling Township, Hunyuan County, Datong City, Shanxi Province |
|  |  | A-3-2 | E：113°72′92.53″ | N：39°44′37.51″ | Wild |  |  |
|  |  | A-3-3 | E：113°72′92.68″ | N：39°44′36.75″ | Wild |  |  |
|  | A4 | A-4-1 | E：113°72′84.11″ | N：39°44′41.56″ | Wild | 50 months (4-year-old) | Kuanping Dongpo Village,Qianfoling Township, Hunyuan County, Datong City, Shanxi Province |
|  |  | A-4-2 | E：113°72′85.70″ | N：39°44′42.10″ | Wild |  |  |
|  |  | A-4-3 | E：113°72′85.95″ | N：39°44′42.55″ | Wild |  |  |
|  | A5 | A-5-1 | E：113°71′88.85″ | N：39°43′50.10″ | Wild | 62 months (5-year-old) | Niuxingbao Village, Qianfoling Township, Hunyuan County, Datong City, Shanxi Province |
|  |  | A-5-2 | E：113°71′89.61″ | N：39° 43′49.00″ | Wild |  |  |
|  |  | A-5-3 | E：113°71′89.63″ | N：39°43′48.65″ | Wild |  |  |
|  | A6 | A-6-1 | E：113°72′19.55″ | N：39°43′83.55″ | Wild | 74 months (6-year-old) | Niuxingbao Village, Qianfoling Township, Hunyuan County, Datong City, Shanxi Province |
|  |  | A-6-2 | E：113°72′18.68″ | N：39°43′84.11″ | Wild |  |  |
|  |  | A-6-3 | E：113°72′18.98″ | N：39°43′83.68″ | Wild |  |  |
|  | B1 | B-1-1 | E：113°20′36.08″ | N：39°45′60.55″ | Cultivated | 14 months  (1 year old) | Zhongcaoshan Village, Ying County, Shuozhou City, Shanxi Province |
|  |  | B-1-2 | E：113°20′34.13″ | N：39°45′60.61″ | Cultivated |  |  |
|  |  | B-1-3 | E：113°20′34.13″ | N：38°93′56.15″ | Cultivated |  |  |
|  | B2 | B-2-1 | E：113°15′76.86″ | N：39°41′14.25″ | Cultivated | 26 months (2-year-old) | Zhongcaoshan Village, Ying County, Shuozhou City, Shanxi Province |
|  |  | B-2-2 | E：113°15′76.86″ | N：39°41′14.25″ | Cultivated |  |  |
|  |  | B-2-3 | E：113°20′36.08″ | N：39°45′60.55″ | Cultivated |  |  |

Table S1. *Cont.*

| Time and purpose | Sample | | Longitude | Latitude | Planting pattern | Growth year | Location |
| --- | --- | --- | --- | --- | --- | --- | --- |
| 2018.10.23-2018.10.24  1. HPLC  2. qRT-PCR | A1 | A-1-1 | E：113°76′16.63″ | N：39°47′98.71″ | Wild | 12 months  (1 year old) | Kuanping Guizigou Village, Qianfoling Township, Hunyuan County, Datong City, Shanxi Province |
|  |  | A-1-2 | E：113°76′22.76″ | N：39°47′92.00″ | Wild |  |  |
|  |  | A-1-3 | E：113°76′18.70″ | N：39°47′92.43″ | Wild |  |  |
|  | A2 | A-2-1 | E：113°76′22.76″ | N：39°47′92.00″ | Wild | 24 months (2-year-old) | Kuanping Guizi Gitch, Qianfoling Township, Hunyuan County, Datong City, Shanxi Province |
|  |  | A-2-2 | E：113°76′22.81″ | N：39°47′91.68″ | Wild |  |  |
|  |  | A-2-3 | E：113°76′20.87″ | N：39°47′94.10″ | Wild |  |  |
|  | A3 | A-3-1 | E：113°76′13.71″ | N：39°47′26.75″ | Wild | 36 months (3-year-old) | Kuanping Dongpo Village,Qianfoling Township, Hunyuan County, Datong City, Shanxi Province |
|  |  | A-3-2 | E：113°76′09.53″ | N：39°47′26.51″ | Wild |  |  |
|  |  | A-3-3 | E：113°76′12.68″ | N：39°47′26.35″ | Wild |  |  |
|  | A4 | A-4-1 | E：113°76′01.71″ | N：39°47′13.93″ | Wild | 48 months (4-year-old) | Kuanping Dongpo Village,Qianfoling Township, Hunyuan County, Datong City, Shanxi Province |
|  |  | A-4-2 | E：113°76′05.26″ | N：39°47′17.10″ | Wild |  |  |
|  |  | A-4-3 | E：113°75′85.95″ | N：39°44′42.25″ | Wild |  |  |
|  | A5 | A-5-1 | E：113°74′28.26″ | N：39°41′25.78″ | Wild | 60 months (5-year-old) | Niuxingbao Village, Qianfoling Township, Hunyuan County, Datong City, Shanxi Province |
|  |  | A-5-2 | E：113°74′38.30″ | N：39°41′15.05″ | Wild |  |  |
|  |  | A-5-3 | E：113°74′89.63″ | N：39°41′43.65″ | Wild |  |  |
|  | A6 | A-6-1 | E：113°74′18.11″ | N：39°41′28.68″ | Wild | 72 months (6-year-old) | Niuxingbao Village, Qianfoling Township, Hunyuan County, Datong City, Shanxi Province |
|  |  | A-6-2 | E：113°74′18.68″ | N：39°41′84.11″ | Wild |  |  |
|  |  | A-6-3 | E：113°73′18.98″ | N：39°41′80.68″ | Wild |  |  |
|  | B1 | B-1-1 | E：113°20′35.80″ | N：39°45′60.26″ | Cultivated | 12 months  (1 year old) | Zhongcaoshan Village, Ying County, Shuozhou City, Shanxi Province |
|  |  | B-1-2 | E：113°20′36.75″ | N：39°45′60.11″ | Cultivated |  |  |
|  |  | B-1-3 | E：113°20′36.24″ | N：39°45′60.15″ | Cultivated |  |  |
|  | B2 | B-2-1 | E：113°20′41.10″ | N：39°45′61.75″ | Cultivated | 24 months (2-year-old) | Zhongcaoshan Village, Ying County, Shuozhou City, Shanxi Province |
|  |  | B-2-2 | E：113°20′38.15″ | N：39°45′61.61″ | Cultivated |  |  |
|  |  | B-2-3 | E：113°20′40.70″ | N：39°45′61.15″ | Cultivated |  |  |

Note: A: imitative wild *A. mongholicus*, B: cultivated *A. mongholicus*; A-1: the number represents growth in years, A-1-1: the first quadrat of A-1.

Table S2. qRT–PCR primers used in this study

| No. | Gene | Primer | Sequence (5'to3') | Number (bp) |
| --- | --- | --- | --- | --- |
| 1 | *18S* | 18S-F | TCAACCATAAACGATGCCGACC | 18 |
|  |  | 18S-R | TTTCAGCCTTGCGACCATACTCC | 18 |
| 2 | *C4H* | c831948-F | GTCCAAAGTAGAAGCCAATG | 20 |
|  |  | c831948-R | CAAACGACCCAAAGTGATA | 19 |
| 3 | *CHS* | c780996-F | CCTGGTCGGACAAGCATT | 18 |
|  |  | c780996-R | TGTTAGCCCAACTTCACG | 18 |
| 4 | *CHR* | c828977-F | ATGGGCAGTGTTAGTGTTGA | 20 |
|  |  | c828977-R | CCTGTACCCTTGTTTGATGG | 20 |
| 5 | *I3'H* | c792702-F | GAGCGATTAGTAGAGGAGCA | 20 |
|  |  | c792702-R | TGAGGCAATAGTAGTGGAGC | 20 |
| 6 | *PAL* | c759450-F | ACTTCTCCAAGGCTACTCAG | 20 |
|  |  | c759450-R | TAAGCAAACCAGCAATGTAA | 20 |
| 7 | *IFS* | c759107-F | GCCATTTGGGTCTGGTAGGA | 20 |
|  |  | c759107-R | TGTGCCCTTGGAACAGTGAG | 20 |
| 8 | *4CL* | c795398-F | TCTCATCAACGGCGACAC | 18 |
|  |  | c795398-R | AGGGCGAATTGAGGACAG | 18 |
| 9 | *UCGT* | c303354-F | AGGAGTGCCGATGGTTAC | 18 |
|  |  | c303354-R | TGCCTCTTCATTCCGTGT | 18 |
| 10 | *IOMT* | c773593-F | TGAGGGAATGGCAAGTGA | 18 |
|  |  | c773593-R | CCCAGCAAGTTAGCGACA | 18 |
| 11 | *UFGT* | c778119-F | GAGGAAAGGACTAAAGGGAG | 20 |
|  |  | c778119-R | GGCCACGTAAGCATTGAC | 18 |

Table S3. Sequence information of UGTs used for phylogenetic analysis

| Name | GenBank | Species | Name | GenBank | Species |
| --- | --- | --- | --- | --- | --- |
| *Ac*F3GT1 | ADC34700 | *Actinidia chinensis* | UGT71A14 | BAG80554 | *Lycium barbarum* |
| UGT73A9 | BAG31950 | *Antirrhinum majus* | UGT73A10 | BAG80536 | *L. barbarum* |
| UGT73E2 | BAG16513 | *A. majus* | UGT71G1 | AAW56092 | *Medicago truncatula* |
| *Ac*GaT | BAD06514 | *Aralia cordata* | UGT78G1 | A6XNC6 | *M. truncatula* |
| UGT89C1 | 6IJD_B | *Arabidopsis thaliana* | UGT71G1 | AAW56092 | *M. truncatula* |
| UGT71B5 | OAO99526 | *A. thaliana* | *Os*79 | Q7XT97 | *Oryza sativa* |
| UGT71B6 | OAP05506 | *A. thaliana* | UGT707A3 | BAC83989 | *O. sativa* |
| UGT71C1 | AEC08300 | *A. thaliana* | UGT84A13 | AHA54051 | *Quercus robur* |
| UGT74C1 | Q9SKC1 | *A. thaliana* | *Ph*A5GT | BAA89009 | *Petunia x hybrida* |
| UGT71C5 | OAP14418 | *A. thaliana* | *Pf*A5GT | BAA36421 | *Perilla frutescens* |
| UGT79B2 | Q9T080 | *A. thaliana* | UGT88D7 | BAG31948 | *P. frutescens* |
| UGT72B1 | OAP00532 | *A. thaliana* | *Pa*GT3 | 6LZX_A | *Phytolacca americana* |
| UGT79B3 | Q9T081 | *A. thaliana* | UGT88A9 | ACB56925 | *Pilosella officinarum* |
| UGT73C6 | OAP07438 | *A. thaliana* | UGT88A8 | ACB56924 | *P. officinarum* |
| UGT76F1 | Q9M052 | *A. thaliana* | *Pt*UGT1 | 5NLM_B | *Polygonum tinctorium* |
| UGT84A1 | OAO99238 | *A. thaliana* | *Pl*UGT1 | EU889119 | *Pueraria lobata* |
| UGT74D1 | OAP11252 | *A. thaliana* | UGT78H2 | AJW67515 | *Rubus hybrid* |
| UGT74E2 | OAP17332 | *A. thaliana* | UGT71A8 | BAF96581 | *Sesamum alatum* |
| UGT74F2 | OAP07463 | *A. thaliana* | UGT71A10 | BAF96583 | *S. radiatum* |
| UGT78D1 | OAP13716 | *A. thaliana* | UGT71A9 | BAF96582 | *S. indicum* |
| UGT78D2 | OAO89857 | *A. thaliana* | UGT94D1 | BAF99027 | *S. indicum* |
| UGT94B1 | Q5NTH0 | *Bellis perennis* | UGT88B1 | AAR06919 | *Stevia rebaudiana* |
| UGT78K6 | 4REM_A | *Clitoria ternatea* | *Th*A5GT | BAC54093 | *Torenia hybrid* |
| UGT707B1 | CCG85331 | *Crocus sativus* | *Vl*RSgt | ABH03018 | *Vitis labrusca* |
| UGT71F3 | BAF75886 | *Dianthus caryophyllus* | *Vv*GT1 | 2C9Z_A | *V. vinifera* |
| *Gh*A5GT | Q9ZR25 | *Glandularia x hybrida* | *Vv*gGT1 | AEW31187 | *V. vinifera* |
| UGT73F1 | BAC78438 | *Glycyrrhiza echinata* | *Vv*gGT3 | AEW31189 | *V. vinifera* |
| UGT74S1 | AFJ52951 | *Linum usitatissimum* | UGT78A12 | BAI22846 | *V. vinifera* |

Table S4. Diameter range of *A. mongholicus* under different growth modes

| Groups | Samples | Diameter range (mm) | Mean (mm) | Standard deviation (mm) |
| --- | --- | --- | --- | --- |
| A1 | 62 | 2.62-3.69 | 3.22 | 0.63 |
| A2 | 51 | 4.74-6.36 | 5.81 | 1.79 |
| A3 | 50 | 4.35-6.49 | 5.51 | 1.31 |
| A4 | 52 | 5.11-11.57 | 8.44 | 3.90 |
| A5 | 24 | 6.52-12.80 | 10.06 | 3.77 |
| A6 | 24 | 7.07-10.69 | 8.59 | 1.84 |
| B1 | 40 | 4.48-8.11 | 6.13 | 2.04 |
| B2 | 28 | 7.45-9.50 | 8.55 | 1.55 |

Table S5. Detailed information on the identified metabolites in *A. mongholicus*

| No. | Rt /min | m/z | Identification | Adducts | Formula | A6vsB2 |
| --- | --- | --- | --- | --- | --- | --- |
| 1 | 1.01 | 146.046 | L-Glutamic acid | M−H | C_21_H_20_O_10_ | 0.00 ↑^**^ |
| 2 | 5.78 | 203.083 | L-Tryptophan | M−H | C_11_H_12_N_2_O_2_ | 0.02 ↓^*^ |
| 3 | 0.97 | 259.022 | Glucose-1-phosphate | M−H | C_16_H_12_O_6_ | 0.00 ↑^**^ |
| 4 | 1.10 | 341.108 | Sucrose | M−H | C_12_H_22_O_11_ | 0.02 ↑^*^ |
| 5 | 11.89 | 283.060 | Calycosin | M−H | C_16_H_12_O_5_ | 0.03 ↓^*^ |
| 6 | 13.41 | 267.065 | Formononetin | M−H | C_16_H_12_O_4_ | 0.02 ↑^*^ |
| 7 | 13.48 | 871.471 | Astragaloside Ⅱ | M+FA−H | C_43_H_70_O_15_ | 0.00 ↑^**^ |
| 8 | 14.56 | 868.485 | Astragaloside Ⅰ | M+FA−H | C_45_H_72_O_16_ | 0.00 ↑^**^ |
| 9 | 18.44 | 279.230 | linolenic acid | M+H | C_18_H_30_O_2_ | 0.00 ↑^**^ |
| 10 | 10.94 | 431.131 | Ononin | M+H | C_22_H_22_O_9_ | 0.04 ↑^*^ |
| 11 | 11.87 | 301.107 | 3-Hydroxy-9,10-dimethoxyptercarpan | M+H | C_17_H_16_O_5_ | 0.04 ↑^*^ |
| 12 | 8.79 | 447.129 | CG | M+H | C_22_H_22_O_10_ | 0.03 ↑^*^ |
| 13 | 14.55 | 870.989 | Isoastragaloside I | M+H | C_45_H_72_O_16_ | 0.00 ↑^**^ |
| 14 | 13.13 | 166.086 | L-phenylalanine | M+H | C_9_H_11_NO_2_ | ↑ |
| 15 | 4.37 | 257.081 | liquiritigenin | M+H | C_15_H_12_O_4_ | ↓ |

Note: ↑: upregulated metabolites in A6, ↓: downregulated metabolites in A6. **P*＜0.05, ***P*＜0.01.

Table S6. The peak area of metabolites in the isoflavone biosynthetic pathway

| Sample  Metabolites | Onoin | CG | Calycosin | Formononetin | L-phenylalanine | Liquiritigenin |
| --- | --- | --- | --- | --- | --- | --- |
|  | Peak area | Peak area | Peak area | Peak area | Peak area | Peak area |
| A1-1 | 915833 | 1896667 | 4752667 | 3059333 | 191033 | 78110 |
| A1-2 | 2010767 | 2966333 | 6230333 | 4966000 | 212200 | 79370 |
| A1-3 | 1174433 | 2644667 | 5887333 | 3359667 | 249300 | 121213 |
| A2-1 | 1344000 | 3575667 | 7457333 | 4903333 | 135833 | 27322 |
| A2-2 | 1733433 | 4487500 | 6302333 | 4752667 | 144490 | 4866 |
| A2-3 | 1575000 | 3099000 | 6995667 | 4763000 | 186067 | 9492 |
| A3-1 | 1695000 | 3490667 | 5908500 | 3873000 | 115880 | 16125 |
| A3-2 | 1085500 | 2630667 | 5487000 | 2868000 | 158800 | 29674 |
| A3-3 | 1675667 | 3745667 | 6929000 | 4277000 | 179420 | 16308 |
| A4-1 | 369833 | 1704000 | 5231333 | 2042333 | 127733 | 8154 |
| A4-2 | 2968000 | 8986333 | 10610667 | 7437667 | 93713 | 58927 |
| A4-3 | 2422000 | 7323000 | 11890000 | 8853000 | 102780 | 19247 |
| A5-1 | 2484333 | 4593667 | 8037667 | 5826000 | 109230 | 33472 |
| A5-2 | 2321667 | 4774333 | 8979333 | 6579667 | 111230 | 10637 |
| A5-3 | 2564667 | 5525667 | 9122333 | 7161667 | 351533 | 14422 |
| A6-1 | 283413 | 569987 | 93681 | 3899245 | 65697 | 17691 |
| A6-2 | 1092506 | 1853729 | 281660 | 1845874 | 55147 | 6435 |
| A6-3 | 531276 | 1374642 | 211288 | 1012580 | 41530 | 3016 |
| B1-1 | 2539500 | 2865000 | 638200 | 413550 | 171093 | 49390 |
| B1-2 | 3351333 | 6307667 | 1526700 | 819367 | 228900 | 87003 |
| B1-3 | 3392667 | 6670667 | 1572867 | 996533 | 320667 | 69941 |
| B2-1 | 350420 | 744044 | 127007 | 1206284 | 93597 | 5441 |
| B2-2 | 308484 | 630886 | 104751 | 1125776 | 164700 | 6219 |
| B2-3 | 293601 | 584296 | 97956 | 1338658 | 234633 | 9498 |

Table S7. Information on candidate genes

| No. | Gene | Unigene ID | Blastx | | Nucleucide blast | | DANMAN |
| --- | --- | --- | --- | --- | --- | --- | --- |
|  |  |  | Query Cover | Per. Identity | Query Cover | Per. Identity | Multi-sequence alignment |
| 1 | *PAL* | c759450_g1 | 90% | 73.46% | 99% | 96.54% | 91.78% |
| 2 | *C4H* | c831948_g1 | 76% | 99.57% | 88% | 98.93% | 75.54% |
| 3 | *4CL* | c795398_g1 | 85% | 84.53% | 86% | 99.27% | 85.97% |
| 4 | *CHS* | c780996_g1 | 83% | 98.46% | 90% | 91.00% | 77.51% |
| 5 | *CHR* | c828977_g1 | 79% | 64.24% | 97% | 97.14% | 79.39% |
| 6 | *CHI* | c801189_g1 | 54% | 99.50% | 72% | 99.43% | 54.12% |
| 7 | *IFS* | c759107_g1 | 81% | 70.49% | 92% | 98.82% | 75.36% |
| 8 | *IOMT* | c773593_g1 | 88% | 100.00% | 88% | 99.62% | 88.48% |
| 9 | *I3'H* | c792702_g1 | 84% | 99.40% | 98% | 98.64% | 84.59% |
| 10 | *UCGT* | c303354_g1 | 62% | 99.58% | 62% | 99.58% | 62.52% |
| 11 | *UFGT* | c778119_g1 | 88% | 76.73% | 99% | 79.71% | 71.02% |

Table S8. FPKM of candidate genes

| Sample | *UCGT* | *PAL* | *C4H* | *CHS* | *CHR* | *CHI* | *IOMT* | *IFS* | *I3'H* | *4CL* | *UFGT* |
| --- | --- | --- | --- | --- | --- | --- | --- | --- | --- | --- | --- |
| A1-1 | 0.54 | 9.17 | 31.92 | 36.18 | 12.53 | 28.61 | 7.82 | 14.65 | 30.6 | 27.17 | 2.22 |
| A1-2 | 0.48 | 13.41 | 28.29 | 35.73 | 14.63 | 29.86 | 1.79 | 10.73 | 36.08 | 16.61 | 1.78 |
| A1-3 | 4.74 | 13.13 | 114.23 | 572.99 | 80.35 | 361.07 | 2.15 | 45.32 | 166.82 | 28.78 | 2.12 |
| A2-1 | 3.17 | 19.68 | 15.87 | 77.36 | 11.97 | 59.08 | 1.58 | 23.32 | 30.48 | 12.64 | 2.83 |
| A2-2 | 4.43 | 9.96 | 27.27 | 62.17 | 10.16 | 56.28 | 1.78 | 40.51 | 24.14 | 13.9 | 2.65 |
| A2-3 | 1.93 | 19.92 | 16.43 | 42.58 | 11.56 | 70.6 | 1.21 | 23.44 | 18.38 | 14.67 | 1.86 |
| A3-1 | 7.41 | 17.65 | 12.17 | 49.57 | 9.67 | 57.12 | 4.92 | 24.84 | 22.62 | 10.95 | 5.68 |
| A3-2 | 2.49 | 11.93 | 7.57 | 26.56 | 5.03 | 52.39 | 3.1 | 24.18 | 20.17 | 8.39 | 1.93 |
| A3-3 | 3.14 | 20.5 | 10.53 | 37.06 | 8.16 | 45.1 | 1.85 | 24.65 | 17.63 | 14.54 | 1.45 |
| A4-1 | 0.43 | 1.09 | 6.47 | 17.03 | 7.66 | 51.06 | 16.3 | 12.15 | 35.04 | 10.97 | 2.06 |
| A4-2 | 0.83 | 4.1 | 17.78 | 54.56 | 9.72 | 55.5 | 10.41 | 13.17 | 47.38 | 20.59 | 2.66 |
| A4-3 | 1.67 | 4.6 | 12.27 | 77.47 | 21.96 | 57.52 | 9.13 | 19.99 | 47.96 | 16.03 | 3.77 |
| A5-1 | 2.17 | 6.3 | 16.47 | 53.72 | 14.14 | 40.42 | 9.75 | 12.14 | 37.47 | 14.11 | 7.14 |
| A5-2 | 1.93 | 3.52 | 8.36 | 34.02 | 10.72 | 47.83 | 14.62 | 10.09 | 30.6 | 17.39 | 4.58 |
| A5-3 | 4.95 | 4.48 | 11.54 | 50.17 | 7.76 | 68.11 | 4.37 | 17.44 | 23.88 | 11.42 | 4.11 |
| A6-1 | 0.68 | 6.02 | 21.33 | 41.18 | 40.99 | 63.32 | 8.04 | 15.96 | 50.48 | 12.07 | 0.72 |
| A6-2 | 2.4 | 34.88 | 9.02 | 71.55 | 13 | 57.11 | 21.19 | 14.36 | 59.22 | 29.01 | 3.41 |
| A6-3 | 1.68 | 10.11 | 8.53 | 61.35 | 23.2 | 67.7 | 14.84 | 19.99 | 67.65 | 24.48 | 2.98 |
| B1-1 | 0.98 | 6.61 | 11.47 | 64.63 | 8.89 | 57.89 | 4.22 | 23.8 | 74.07 | 20.97 | 2.81 |
| B1-2 | 0.44 | 8.33 | 24.1 | 42.4 | 14.5 | 72.52 | 4.34 | 14.19 | 42.81 | 21.96 | 0.94 |
| B1-3 | 0.7 | 9.83 | 7.64 | 33.11 | 10.57 | 61.86 | 8.48 | 11.52 | 59.34 | 21.75 | 2.11 |
| B2-1 | 0.54 | 3.47 | 14.73 | 38.92 | 6.79 | 48.78 | 15.24 | 12.73 | 47.58 | 25.78 | 1.66 |
| B2-2 | 0.33 | 1.79 | 21.64 | 25.4 | 4.63 | 42.92 | 7.64 | 5.09 | 51.12 | 41.11 | 4.53 |
| B2-3 | 1.03 | 7.14 | 38.35 | 49.31 | 11.73 | 36.08 | 28.66 | 9.25 | 52.67 | 28.29 | 4.84 |

Table S9. Pearson correlation analysis of 11 isoflavone-related genes by qRT–PCR

| Gene  Gene | *4CL* | *C4H* | *CHI* | *CHR* | *CHS* | *I3'H* | *IFS* | *IOMT* | *PAL* | *UCGT* | *UFGT* |
| --- | --- | --- | --- | --- | --- | --- | --- | --- | --- | --- | --- |
| *4CL* | 0.00^**^ | 0.98 | 0.51 | 0.26 | 0.53 | 0.65 | 0.00^**^ | 0.56 | 0.00^**^ | 0.51 | 0.80 |
| *C4H* | 0.98 | 0.00^**^ | 0.00^**^ | 0.00^**^ | 0.00^**^ | 0.00^**^ | 0.80 | 0.00^**^ | 0.29 | 0.01^*^ | 0.01^*^ |
| *CHI* | 0.51 | 0.00^**^ | 0.00^**^ | 0.00^**^ | 0.00^**^ | 0.00^**^ | 0.62 | 0.01^*^ | 0.96 | 0.00^**^ | 0.42 |
| *CHR* | 0.26 | 0.00^**^ | 0.00^**^ | 0.00^**^ | 0.00^**^ | 0.00^**^ | 0.21 | 0.08 | 0.07 | 0.00^**^ | 0.22 |
| *CHS* | 0.53 | 0.00^**^ | 0.00^**^ | 0.00^**^ | 0.00^**^ | 0.00^**^ | 0.65 | 0.00^**^ | 0.62 | 0.01^*^ | 0.06 |
| *I3'H* | 0.65 | 0.00^**^ | 0.00^**^ | 0.00^**^ | 0.00^**^ | 0.00^**^ | 0.71 | 0.00^**^ | 0.58 | 0.01^*^ | 0.19 |
| *IFS* | 0.00^**^ | 0.80 | 0.62 | 0.21 | 0.65 | 0.71 | 0.00^**^ | 0.48 | 0.00^**^ | 0.37 | 0.80 |
| *IOMT* | 0.56 | 0.00^**^ | 0.01^*^ | 0.08 | 0.00^**^ | 0.00^**^ | 0.48 | 0.00^**^ | 0.49 | 0.29 | 0.00^**^ |
| *PAL* | 0.00^**^ | 0.29 | 0.96 | 0.07 | 0.62 | 0.58 | 0.00^**^ | 0.49 | 0.00^**^ | 0.03^*^ | 0.04^*^ |
| *UCGT* | 0.51 | 0.01^*^ | 0.00^**^ | 0.00^**^ | 0.01^*^ | 0.01^*^ | 0.37 | 0.29 | 0.03^*^ | 0.00^**^ | 0.05 |
| *UFGT* | 0.80 | 0.01^*^ | 0.42 | 0.22 | 0.06 | 0.19 | 0.80 | 0.00^**^ | 0.04^*^ | 0.05 | 0.00^**^ |

Note: **P*＜0.05, ***P*＜0.01.

Table S10. Pearson correlation analysis of 11 isoflavone-related genes and 6 metabolites

| Gene  Metabolites | Calycosin | CG | Formononetin | L-phenylalanine | Liquiritigenin | Onoin |
| --- | --- | --- | --- | --- | --- | --- |
| *4CL* | 0.67 | 0.86 | 0.95 | 0.93 | 0.93 | 0.88 |
| *C4H* | 0.26 | 0.69 | 0.12 | 0.54 | 0.08 | 0.39 |
| *CHI* | 0.37 | 0.18 | 0.56 | 0.85 | 0.48 | 0.38 |
| *CHR* | 0.98 | 0.62 | 0.59 | 0.60 | 0.03^*^ | 0.61 |
| *CHS* | 0.34 | 0.41 | 0.22 | 0.75 | 0.41 | 0.24 |
| *I3'H* | 0.71 | 0.47 | 0.46 | 0.31 | 0.03^*^ | 0.29 |
| *IFS* | 0.83 | 0.83 | 0.86 | 0.91 | 0.93 | 0.87 |
| *IOMT* | 0.54 | 0.73 | 0.58 | 0.53 | 0.80 | 0.76 |
| *PAL* | 0.86 | 0.37 | 0.80 | 0.39 | 0.93 | 0.36 |
| *UCGT* | 0.94 | 0.85 | 0.99 | 0.19 | 0.68 | 0.68 |
| *UFGT* | 0.52 | 0.57 | 0.86 | 0.01^*^ | 0.59 | 0.37 |

Note: **P*＜0.05, ***P*＜0.01.


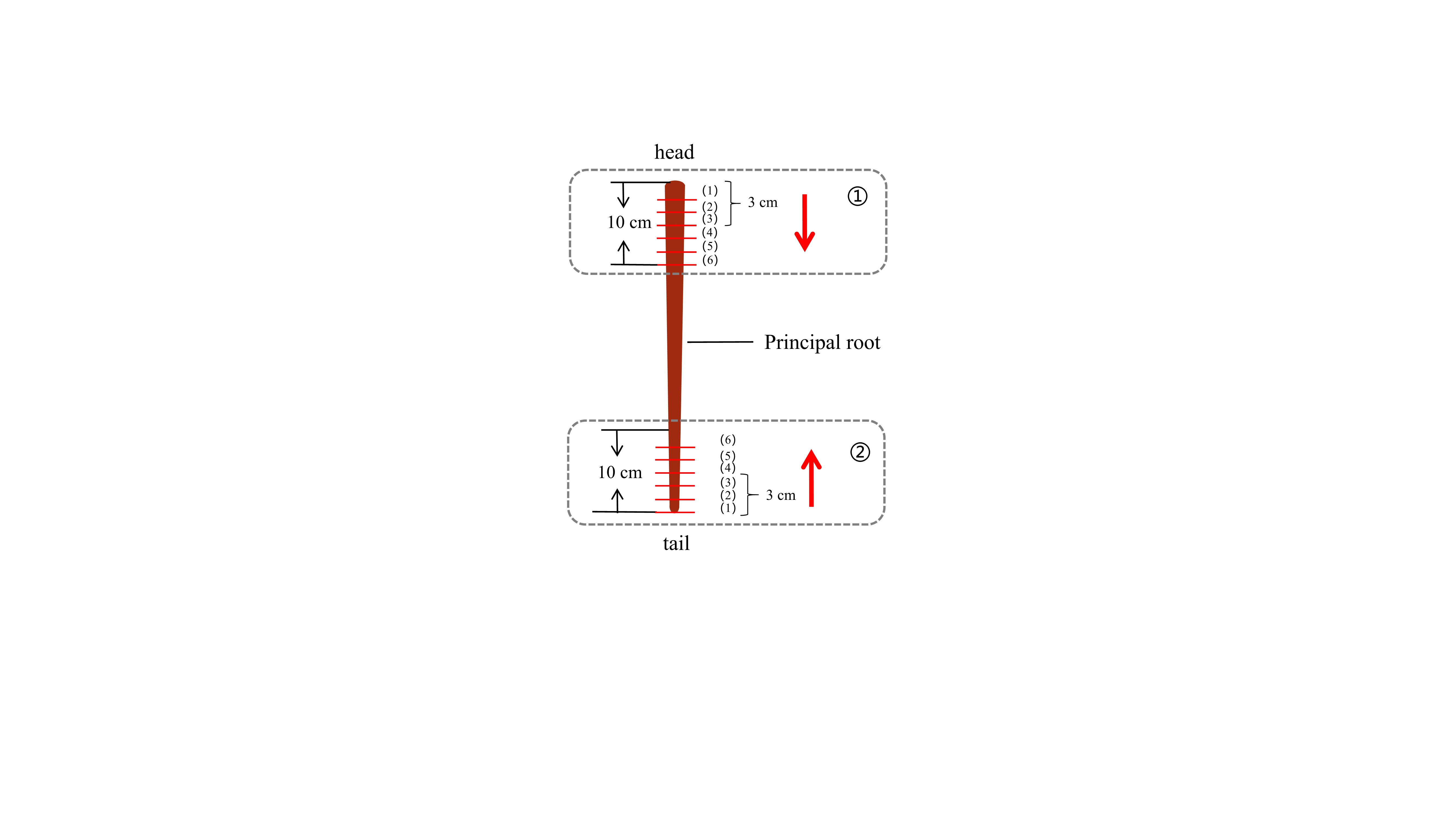


Figure S1. Schematic diagram of the processing method about *A. mongholicus*

Note: (1)/(2)/(3)/(4)/(5)/(6): the slices of the sample, the thickness of each slice is about 1-2cm. The red arrow indicates the direction of slicing. For A2-A6 and B2, the rhizomes and lateral roots of each sample were removed first. The principal roots and thick lateral roots were retained. **UHPLC-ESI-Q-TOF-MS/MS, Iso-Seq, and RNA-Seq**: The first part (①) was cut out following the direction from head to tail, while the other part (②) was cut out from tail to head. Each part was about 3 cm. Then, the above two parts (① and ②)were mixed sequentially. All samples were wrapped with aluminum foil and immediately placed in liquid nitrogen. Then, they were stored at −80 °C for preservation. **HPLC and qRT-PCR**: Two-part (① and ②) were cut out separately, then mixed sequentially. Each part was about 10 cm. The samples for qRT-PCR were wrapped with aluminum foil and immediately placed in liquid nitrogen, while the samples for HPLC just need to dry at room temperature.


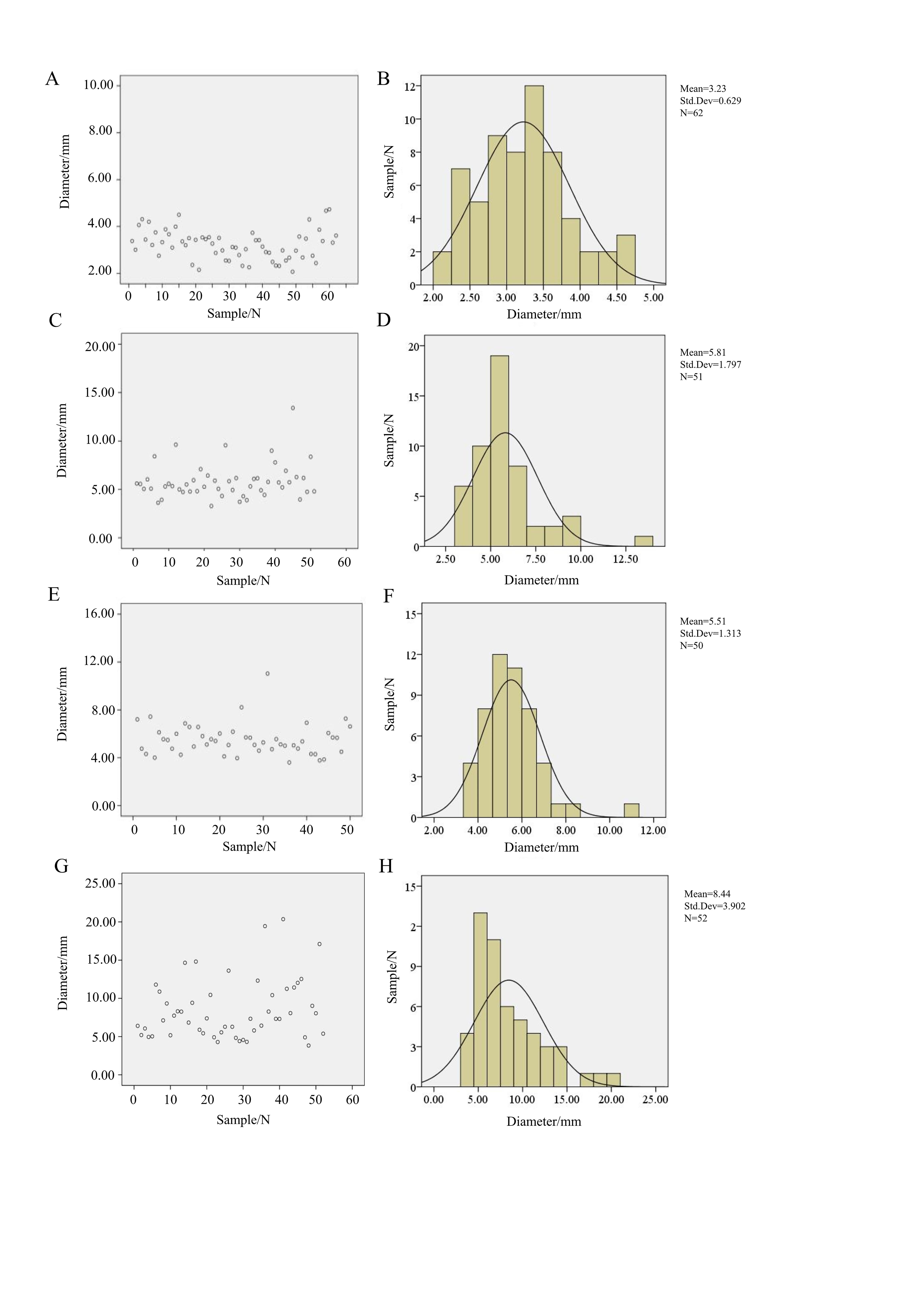


Figure S2. Diameter scatter plot and frequency distribution histogram of *A. mongholicus*

(A/B: A1, C/D: A2, E/F: A3, G/H: A4)


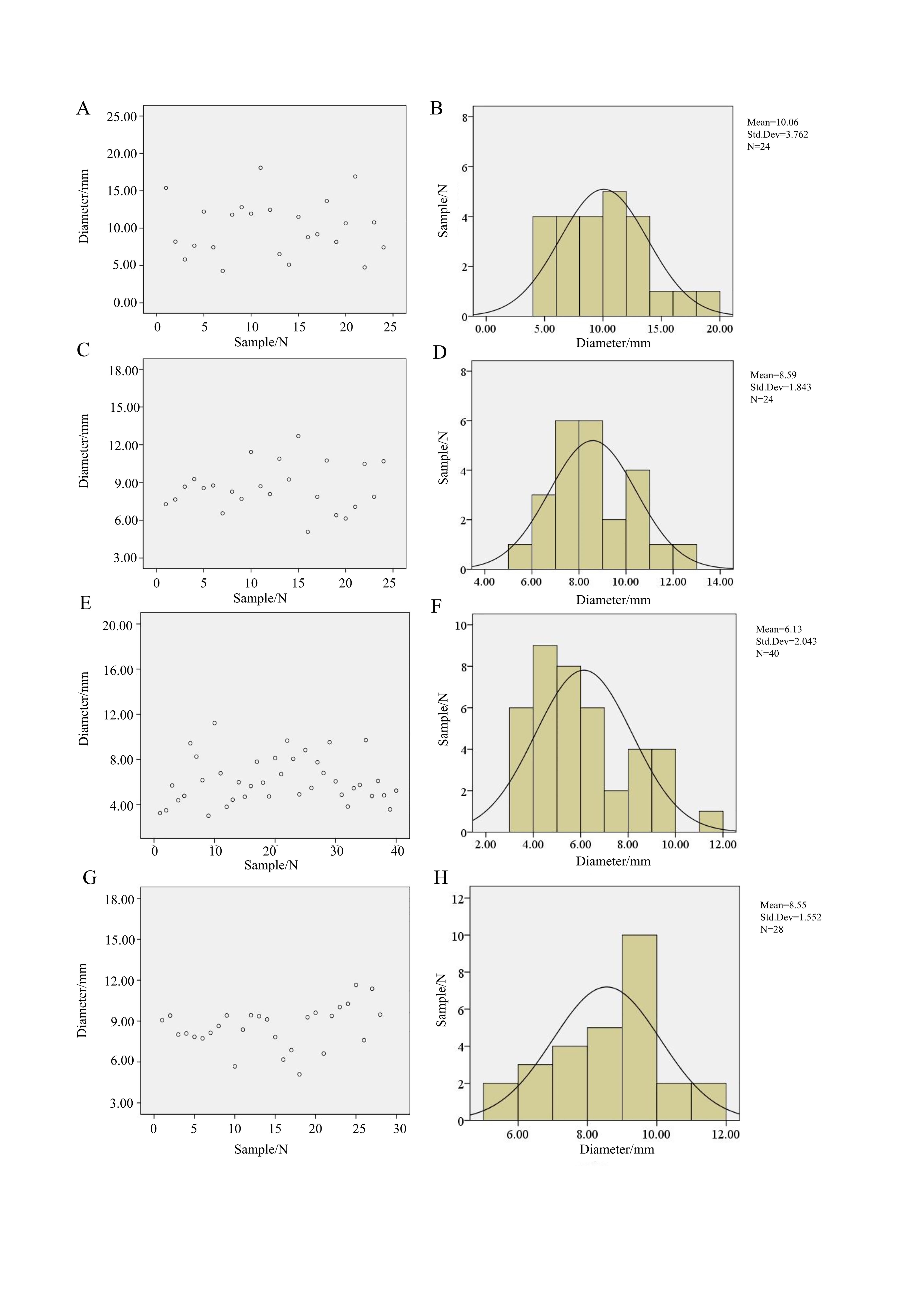


Figure S3. Diameter scatter plot and frequency distribution histogram of *A. mongholicus*

(A/B: A5, C/D: A6, E/F: B1, G/H: B2)


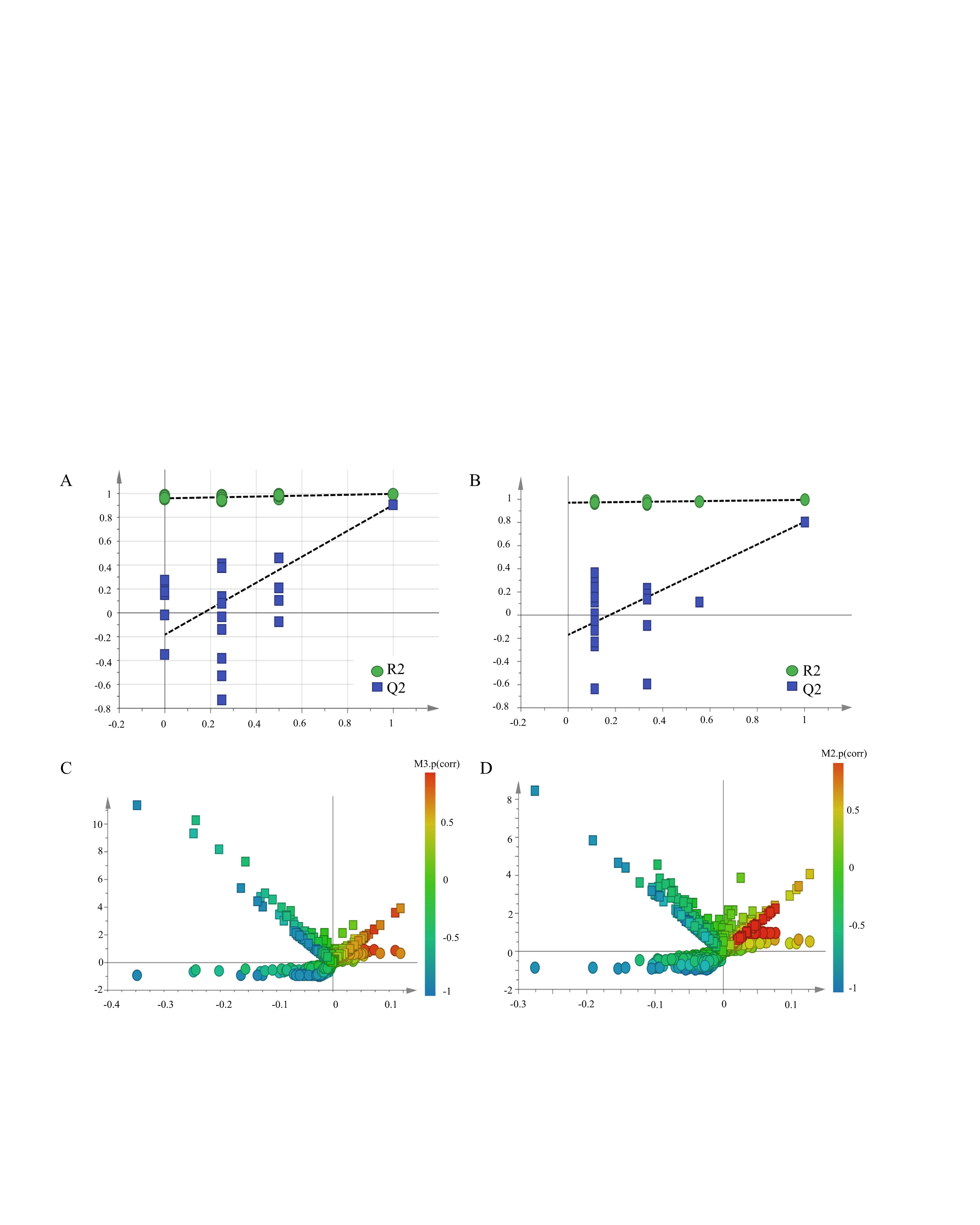


Figure S4. Analysis of metabolomics for A6 and B2. (A) PLS-DA of A6 and B2 in POS. (B) PLS-DA of A6 and B2 in NEG. (C) Scatter plot of A6 and B2 in POS. (D) Scatter plot of A6 and B2 in NEG.


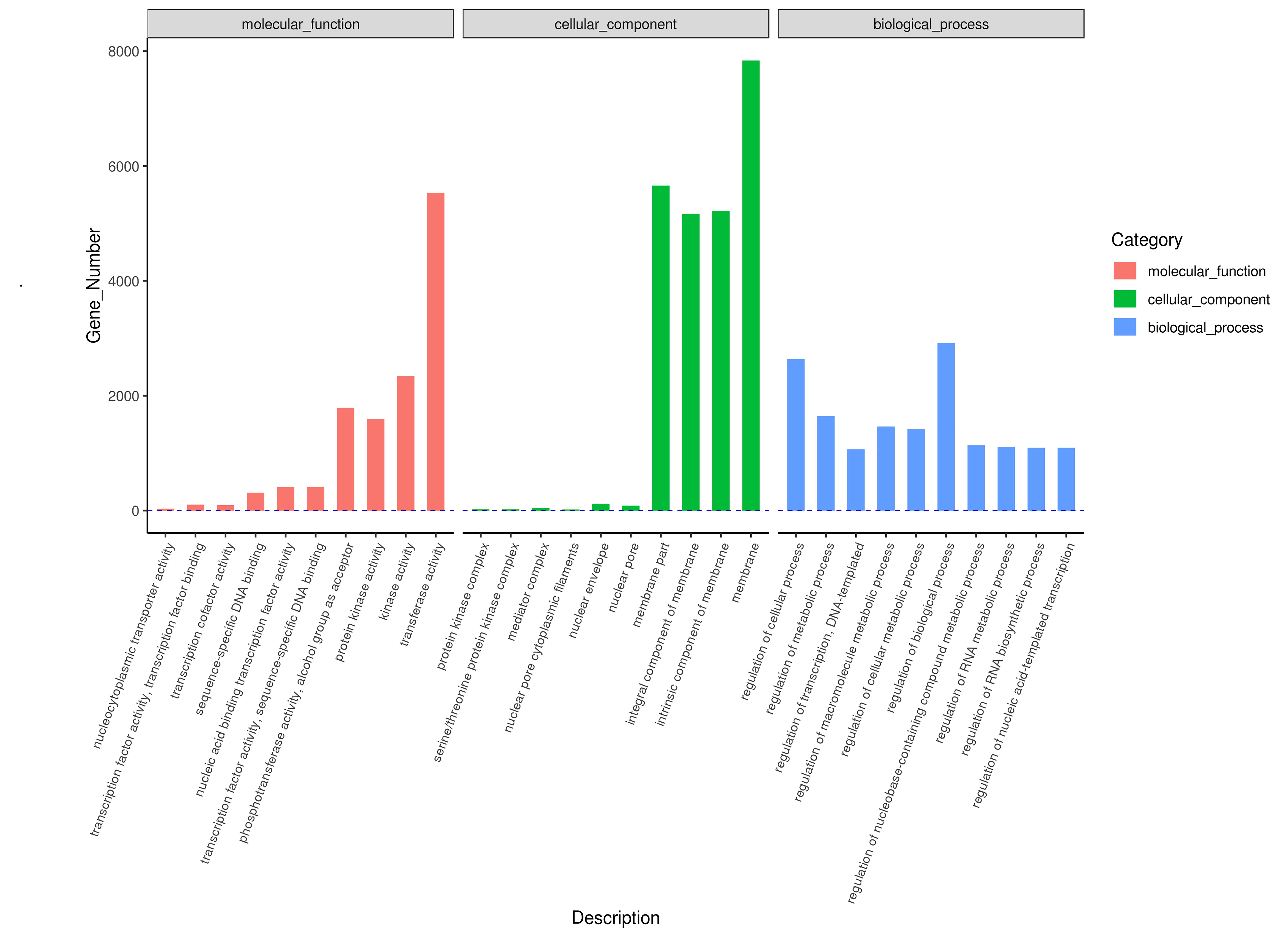


Figure S5. Distribution of GO terms for all annotated unigenes in the biological process, cellular component, and molecular function categories.


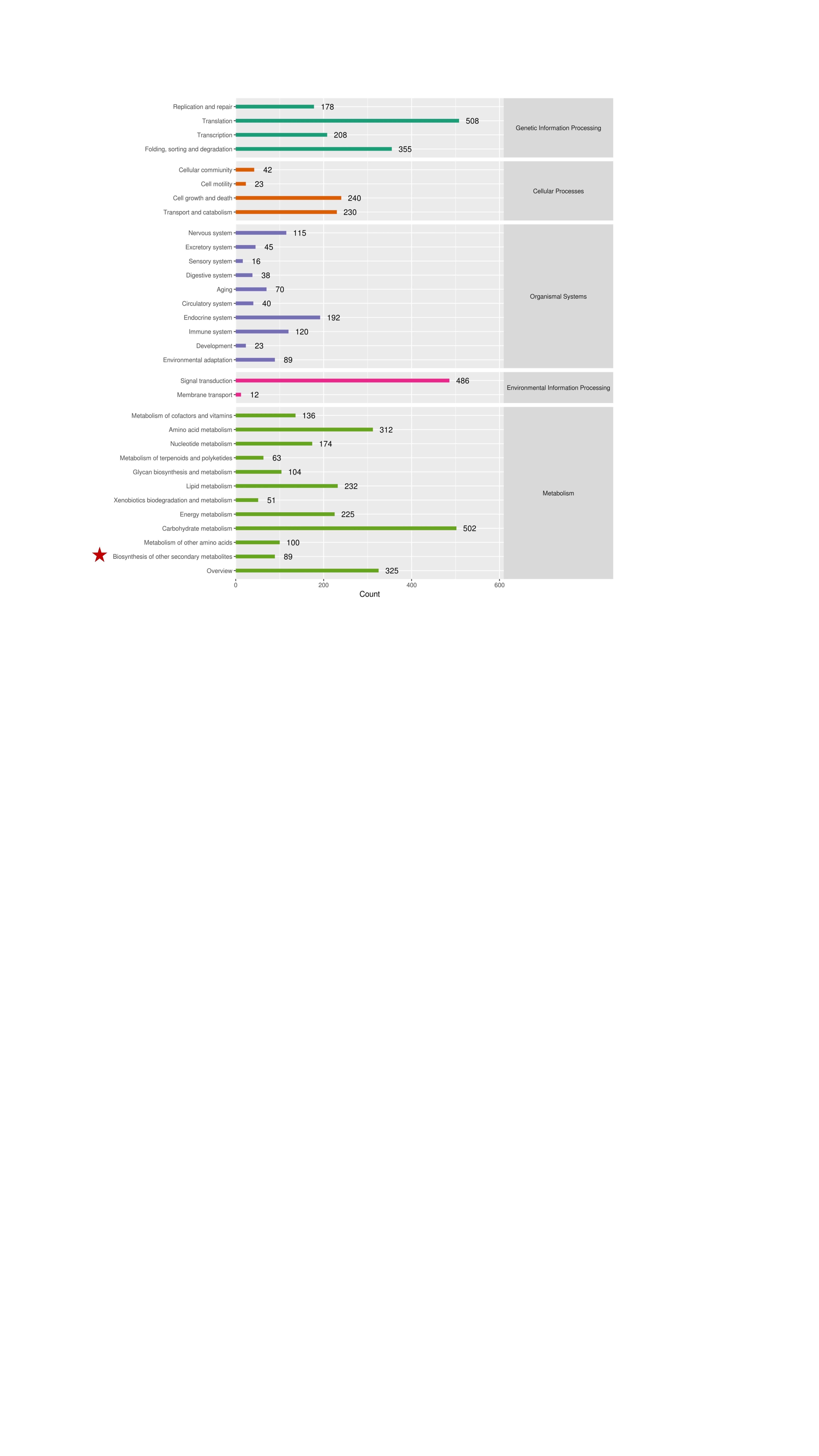


Figure S6. KEGG pathways enriched by unigenes.

(The five-point star indicates the biosynthesis of secondary metabolites)


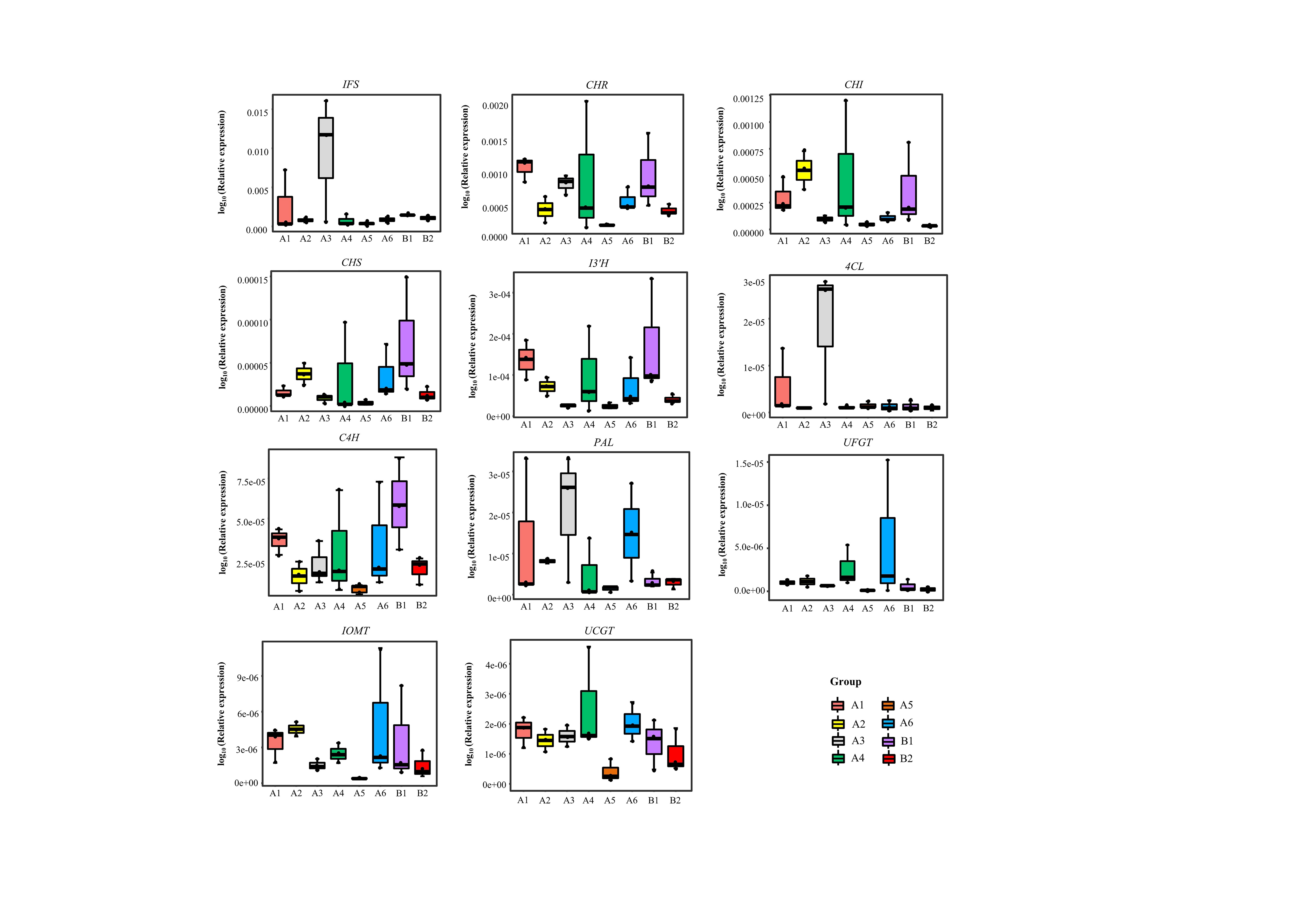


Figure S7. Expression of isoflavone-related genes in *A. mongholicus* by qRT–PCR analysis.


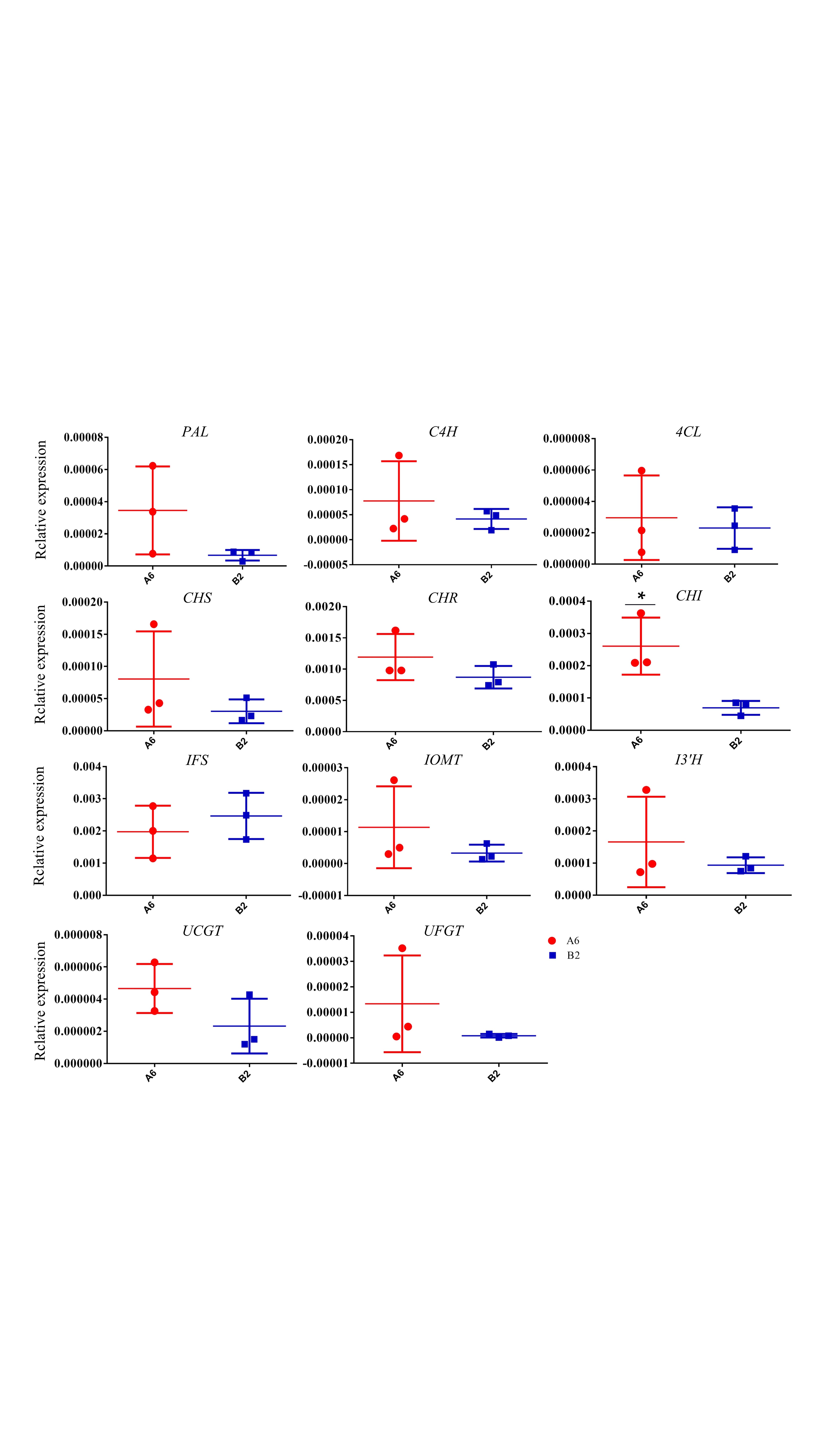


Figure S8. Differential expression of isoflavone-related genes in A6 vs. B2 by qRT–PCR analysis. **P*＜0.05


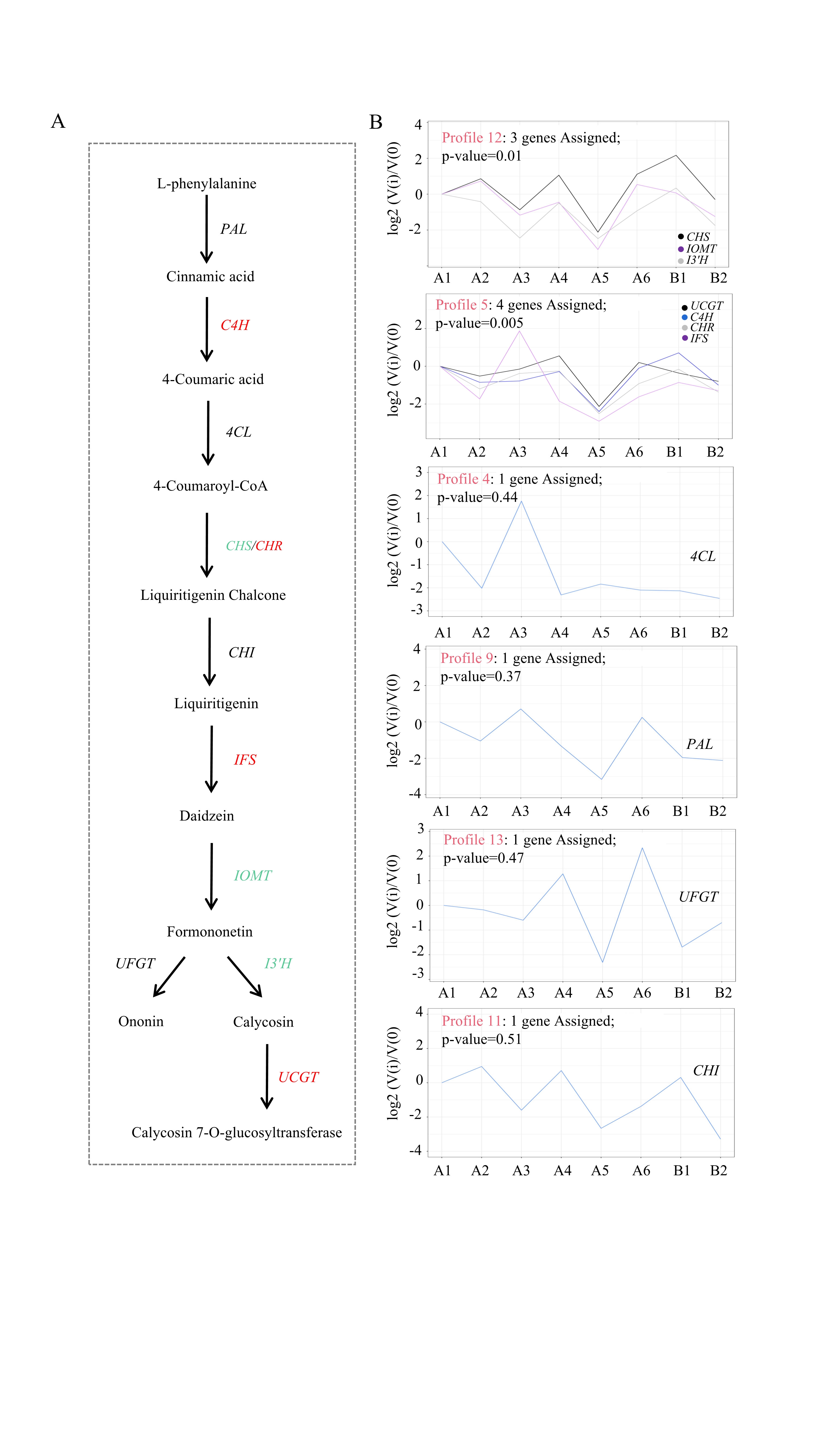


Figure S9. STEM analysis of isoflavone-related genes by qRT–PCR. The results were analyzed by STEM online at <http://www.omicshare.com/.> (A) Isoflavone biosynthetic pathway; the same color indicates that the profile of those genes was the same. Black indicates that the gene had a separate profile. (B) Profiles of isoflavone-related genes in different samples. (Note: Profile 5 included *IFS*, *C4H*, *CHR*, and *UCGT*, and profile 13 included *CHS*, *IOMT*, and *I3′H*, indicating that the abovementioned genes had the same change trends (Fig. S9B). The other four profiles were not significantly enriched and included *PAL*, *4CL*, *CHI*, and *UFGT* corresponding to profile 4, profile 9, profile 11, and profile 13, respectively (Fig. S9B). The results further suggested that *CHS*, *CHR*, and *IFS* had a similar expression pattern to *UCGT* between WAM and CAM. Likewise, *CHS* and *IOMT* shared the same expression pattern as *I3′H* (Fig. S9A)).


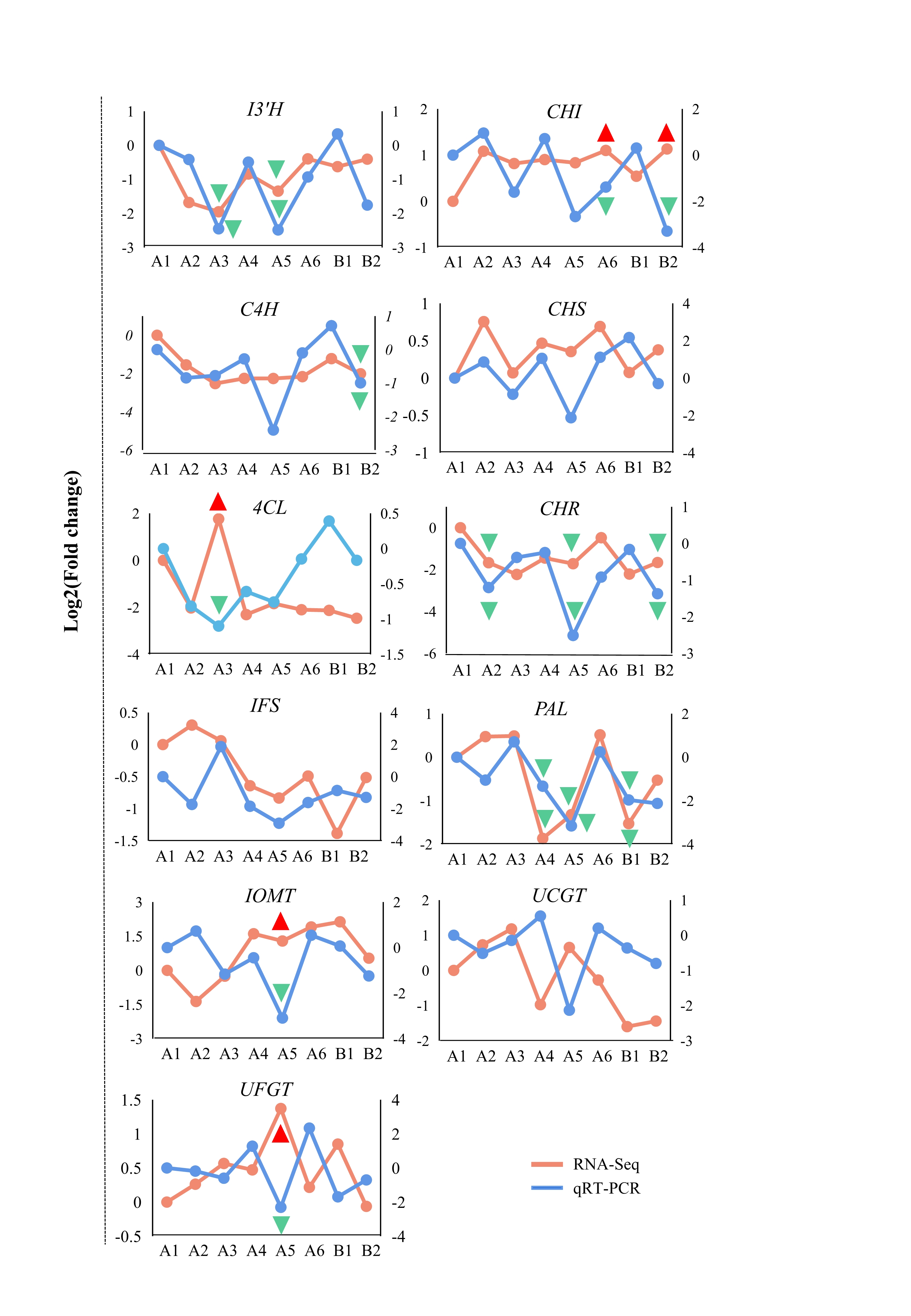


Figure. S10 The integrated analysis of qRT–PCR and RNA-Seq validation for 11 isoflavone-related genes in *A. mongholicus.* Fold change in expression is calculated by the ratio of other samples to A1.
