# Supplementary material for: Biosynthetic mechanisms of isoflavone accumulation affected by different growth patterns in Astragalus mongholicus products
Source: BMC Plant Biol. 2022 Aug 23;22:410. doi: 10.1186/s12870-022-03769-5 (PMC9396891; doi:10.1186/s12870-022-03769-5)
Supplement: Supplementary file 2 — Additional file 2. The sequences of candidate genes. [file 12870_2022_3769_MOESM2_ESM.docx]

**>c759450_g1**

**Gene:**

CAGAAAATCAATTCTCCACAACTCTCTTTCATAACGTTTTCTTTCTCTCCATATTTTTAATTTCTTTCCTTCAATTTTCCTCTCTAAAACATTTGTATTAACAAAATGGAAACTGCAACCATTACCAACAACTTTAACGGTGGTTCATTCTGTTTGAAGAATAGTACTATCAATGCTAGTGATCCATTGAATTGGGGTGTGGCTGCGGAGTCAATGAAGGGGAGTCACCTAGATGAGGTGAAGCGGATGGTGGAGGAGTACCGGACACCGGTGGTCAGGCTCGGTGGGGAGACGTTGACAATCTCGCAGGTGGCTGCGATCGCCGGAGCATACGATGATGGTGTGAGGGTGGAGCTGTCGGAATCTGCAAGGGCTGGTGTTAAGGCTAGTAGTGACTGGGTGATGAACAGTATGAACAATGGCACTGACAGTTACGGTGTTACCACCGGCTTCGGTGCTACCTCTCACCGCCGAACTAAACAAGGTGGCGCTTTGCAGAAAGAACTAATCAGATTTTTAAATGCCGGAATATTTGGCAATGGGACAGAGTCAAGCCACACGCTACCACAAACCGCAACAAGAGCAGCCATGTTAGTGAGAATCAACACACTTCTCCAAGGCTACTCAGGCATTAGATTTGAAATCTTAGAGGCCATAACCAAGCTTCTCAACAACAATATCACCCCATGTTTACCACTTCGTGGTACAATCACAGCTTCAGGAGATTTGGTCCCTCTTTCTTACATTGCTGGTTTGCTTACTGGTAGGCCAAACTCCAAAGCTGTTGGACCCTCTGGAGAGGTACTCAATGCAAAGGAAGCTTTTCAATTAGCCGGAATCAATGATGGTTTCTTTGAATTGCAGCCCAAAGAAGGCCTTGCACTTGTCAATGGCACTGCAGTTGGTTCTGCCTTAGCTTCTATCGTTCTCTTTGATGCTAACATATTGGTTGTCTTGTCTGAAGTTCTATCAGCTATATTTGCTGAAGTCATGCAAGGAAAACCAGAATTTACTGACCATTTGACACACAAGTTGAAGCACCACCCTGGTCAAATTGAAGCCGCTGCTATTATGGAACATATTTTGGATGGAAGTTCTTATGTCAAAGCAGATAAGAAGCTGCATGAGATGGATCCTTTACAGAAGCCAAAACAAGATAGATATGCACTTAGAACATCGCCACAATGGCTTGGTCCTCTTATTGAAGTCATTAGATTCTCTACCAAGTCAATTGAGAGAGAGATTAACTCTGTCAATGACAACCCTTTGATTGATGTTTCAAGAAACAAGGCCCTACATGGTGGAAACTTCCAAGGAACACCTATTGGAGTCTCCATGGACAACACACGTTTGGCACTTGCAGCAATTGGAAAGCTCATGTTTGCTCAATTCTCTGAGCTTGTTAATGACTTTTACAACAATGGGTTGCCTTCAAATCTCTCTGCTAGTAGAAATCCCAGCTTGGATTATGGTTTCAAGGGAGCTGAAATTGCCATGGCGTCTTATTGTTCCGAGCTACAATATCTTGCAAATCCCGTCACAACTCATGTTCAAAGTGCTGAGCAGCATAACCAAGATGTGAATTCTTTGGGCTTGATTTCATCTAGGAAAACATATGAAGCCATTGAGATCCTTAAGTTAATGTCTTCCACATTTTTGATTGCACTTTGCCAAGCAATTGACTTGAGGCATTTGGAGGAGAACTTGAAAAGCTCAGTAAAGGACTGTGTAAGCCAAGTTGCCAAGAGGACCCTTACCATAGGTTTCAATGGAGAACTCCATCCTTCAAGGTTTTGTGAAAAAGATTTGTTGAAAGTGGTTGAAAGGGAACACGTGTTTGCCTATATTGATGACGCATGCAGTGCTACATACCCATTGATGCAAAAGCTAAGGCAAGTGCTTGTTGACCATGCATTAGTAAATGGTGAAAGTGAGAAGAACTTGAACACATCAATCTTCCAAAAGATTTCTACTTTTGAGGATGAGTTGAAGACCCTTTTACCAAAGGAGGTTGAAAGTACAAGGATTGCATATGAGAGTGGCAATTCACCAATTCCAAACAAGATCAATGGGTGCAGATCATACCCACTTTATAAGTTTGTGAGAGAGGAGTTGGGGACTGGTTTGCTAACTGGAGAAAATGTCATATCACCTGGTGAAGAATTTGACAAACTATTCACAGCTATGTGTCAAGGCAAAATCATTGATCCTCTTTTGGAATGTCTTGGGGAGTGGAACGGTGCCCCTCTTCCGATTTGTTAATTTTATCATTATTTTAGAAATATTTTATTGTATTTATACAAAGTACACCGATAATCATTTG

**CDS:**

QKINSPQLSFITFSFSPYF-FLSFNFPL-NICINKMETATITNNFNGGSFCLKNSTINASDPLNWGVAAESMKGSHLDEVKRMVEEYRTPVVRLGGETLTISQVAAIAGAYDDGVRVELSESARAGVKASSDWVMNSMNNGTDSYGVTTGFGATSHRRTKQGGALQKELIRFLNAGIFGNGTESSHTLPQTATRAAMLVRINTLLQGYSGIRFEILEAITKLLNNNITPCLPLRGTITASGDLVPLSYIAGLLTGRPNSKAVGPSGEVLNAKEAFQLAGINDGFFELQPKEGLALVNGTAVGSALASIVLFDANILVVLSEVLSAIFAEVMQGKPEFTDHLTHKLKHHPGQIEAAAIMEHILDGSSYVKADKKLHEMDPLQKPKQDRYALRTSPQWLGPLIEVIRFSTKSIEREINSVNDNPLIDVSRNKALHGGNFQGTPIGVSMDNTRLALAAIGKLMFAQFSELVNDFYNNGLPSNLSASRNPSLDYGFKGAEIAMASYCSELQYLANPVTTHVQSAEQHNQDVNSLGLISSRKTYEAIEILKLMSSTFLIALCQAIDLRHLEENLKSSVKDCVSQVAKRTLTIGFNGELHPSRFCEKDLLKVVEREHVFAYIDDACSATYPLMQKLRQVLVDHALVNGESEKNLNTSIFQKISTFEDELKTLLPKEVESTRIAYESGNSPIPNKINGCRSYPLYKFVREELGTGLLTGENVISPGEEFDKLFTAMCQGKIIDPLLECLGEWNGAPLPIC-FYHYFRNILLYLYKVHR-SF

**>c831948_g1**

**Gene:**

ACAACAACACACCACACACAAATCCCTAATTCCCCATTCTATCCTTCACATTCCACGATGGATCTCCTCCTCTTAGAGAAGATCCTAACCGCTCTCTTCGCAACCGCAGTAGTTGCCATTGCCGTCGCGAAACTTCGTGGCAAGCGTTTCAAGCTTCCGCCGGGGCCTCTCCCGGTGCCAATCTTCGGTAACTGGCTCCAAGTAGGCGATGATCTCAACCACCGAAACCTCACCGATTTAGCAAAGAAATTCGGCGATATCTTCCTCCTCCGCATGGGACAGCGTAATCTGGTGGTAGTTTCCTCGCCGGAGCTTGCTAAAGAAGTGCTCCACACACAAGGTGTGGAGTTCGGCTCCCGAACGCGCAACGTCGTCTTCGACATTTTCACCGGAAAGGGACAAGACATGGTGTTCACTGTTTACGGCGAACACTGGCGGAAGATGCGGCGGATCATGACGGTGCCGTTCTTCACTAATAAGGTTGTGCAGCAGTACCGCTTTGGTTGGGAAGACGAAGCGGCCAGCGTCGTTGAGGATGTGCGGCGGAACCCTGAGTCGGCGACTAACGGCATCGTTTTACGGAAACGGTTGCAGATGCTGATGTATAACAATATGTACAGGATTATGTTCGATAGAAGGTTCGAAAATGAGGATGATCCTTTGTTCGAGAAGCTTAAGGTTTTAAACGGTGAGAGGAGCCGTTTGGCTCAGAGCTTTGATTATAACTACGGTGATTTTATCCCTATTTTGAGACCTTTCTTGCGAGGCTATTTGAAGATCTGTAAGGAAGTTAAGGAACGCAGGTTGCAACTCTTCAAGGATTATTTCGTTGAAGACAGAAAGAATATTGGAAGTACAAAGAGAACTGATAATGAAGGACTCAAGTGTGCGATCGATCATATTTTGGATGCCCAGAAGAAGGGCGAGATCAGCGAGGACAATGTTCTTTACATTGTTGAGAATATTAACGTTGCTGCAATAGAGACAACTCTGTGGTCAATTGAATGGGGCATTGCAGAGCTTGTGAACCACCCAGAGATCCAGAAGAAGGTCCGGGAAGAGATTGACAGAGTTCTTGGACCTGGTCACCAAGTGACAGAGCCAGATACTCACAAGCTCCCTTACCTGCAAGCTGTGATCAAGGAAACACTCCGCCTTCGAATGGCGATTCCACTTCTTGTTCCACACATGAACCTTCACGATGCAAAGCTTGGTGGTTTTGACATTCCAGCTGAAAGCAAGATCTTGGTCAATGCATGGTGGCTTGCCAACAACCCTGCTCACTGGAAAAACCCTGAGCAGTTCAGGCCTGAGAGGTTCTTGGAGGAAGAGTCCAAAGTAGAAGCCAATGGGAATGACTTTAGGTACCTTCCCTTCGGTGTTGGAAGAAGGAGCTGCCCTGGAATTATTCTTGCATTGCCTATACTTGGTATCACTTTGGGTCGTTTGGTCCAAAATTTGAGCTATTGCCTCCTCCAGGACAGTCCAAGCTTGATACTACTGAGAAAGGAGGGCAGTTTAGTTTGCATATACTGAAACATTCTACCATCGTCTTAAAGCCAAGATCATTTTAGTCTTCAGAATAAATGAATCCCTTTATTCTTTTAGCGTATTTCTGTATCTTATCAATGTTGTGACTGTTTCAAAATGGGGATGTCGTAGAAGTGTCAGCTTGGCCTGCCTATGCATACGGGTGCACTATTATTCTGTGCATGTCTTGCTGTAAATTGCTCAACTAGAAATGTTGGCTTTGTGGAAATCTTGAAATAAAGTCCATGCTATGCTCCCTATCTTGTTATTGCAATATTGTACTATCAATTATCATCATCTTAGCTTAGCCTTTAGGTATTTGATTAACTTGATCGAAAGCCAATATAAGGTAGGCAATGAAGATCACGAGATCAGCCAAATAAAGCTCTTTGCAAGTCCAAAAGGTTGATCTCAAATGGGAATTTTTCACAAGCATCTCAAAAGCAAACTTCACCTATAT

**CDS:**

TTTHHTQIPNSPFYPSHSTMDLLLLEKILTALFATAVVAIAVAKLRGKRFKLPPGPLPVPIFGNWLQVGDDLNHRNLTDLAKKFGDIFLLRMGQRNLVVVSSPELAKEVLHTQGVEFGSRTRNVVFDIFTGKGQDMVFTVYGEHWRKMRRIMTVPFFTNKVVQQYRFGWEDEAASVVEDVRRNPESATNGIVLRKRLQMLMYNNMYRIMFDRRFENEDDPLFEKLKVLNGERSRLAQSFDYNYGDFIPILRPFLRGYLKICKEVKERRLQLFKDYFVEDRKNIGSTKRTDNEGLKCAIDHILDAQKKGEISEDNVLYIVENINVAAIETTLWSIEWGIAELVNHPEIQKKVREEIDRVLGPGHQVTEPDTHKLPYLQAVIKETLRLRMAIPLLVPHMNLHDAKLGGFDIPAESKILVNAWWLANNPAHWKNPEQFRPERFLEEESKVEANGNDFRYLPFGVGRRSCPGIILALPILGITLGRLVQNLSYCLLQDSPSLILLRKEGSLVCIY-NILPSS-SQDHFSLQNK-IPLFF-RISVSYQCCDCFKMGMS-KCQLGLPMHTGALLFCACLAVNCSTRNVGFVEILK-SPCYAPYLVIAILYYQLSSS-LSL-VFD-LDRKPI-GRQ-RSRDQPNKALCKSKRLISNGNFSQASQKQTSPI

**>c795398_g1**

**Gene:**

AAGCAAACTTCTCCAAATTAAATCAAAATGTCGCCAGCTCCTACACAAGAAGAATTCATATTCCGCTCTAAACTCCCCAACATTCCCATCCCAACACACCTCCCTTTACACTCTTACTGTTTCGAAAATCTCTCCAAATTCCACCACCGTCCATGTCTCATCAACGGCGACACCGGGGAAACCCTCACCTACGCCGAAGTCCACCTCACCGTCCGCAAAATCGTCGCCGGTCTCAACTCCCTTAACATCCATCAAGGTGACGTCATCATGCTTGTCCTCCGCAACTGTCCTCAATTCGCCCTCTCCTTCCTCGGTGCCGCCCACCGCGGCGCCGTAATCACCACCGCTAACCCCTTCTACACTACGACGGAGCTCGCCAAACAAGCAACCGCCACGAAATCAAAACTCATCATAACGCAATCCGCATACGTGGAGAAAATCAAACATTTCGCAAAAACAAATGATATCAAAATAGTGTCCATTGATTCCTCTACAGAGGAAGAAGATGTTCTGCATTTCTCCGTTTTAACACAAGCTAACGAAAACGACGCGCCTGATGTTAAAATCAACCCAGACGACGTCGTTGCACTTCCGTTTTCTTCCGGCACGTCAGGACTTCCAAAAGGAGTGATGTTAACGCATAAAAACTTGGTGACAACGATAGCGCAGTTAGTTGACGGCGAAAATCCACACCAGTATACTAACTGTGAGGATGTGTTACTCTGTGTGTTGCCTATGTTTCACATCTATGCGCTTAATTCAATTTTATTGTGTGGGATTCGTTCTGGTGCGGCCGTGCTTATTGTGCAGAAGTTTGAGATTACTACGCTGTTGGAACTTATTCAGAAGTATAGGGTGACGGTAGCGTCGTTTGTGCCGCCAATTGTTTTGGCGTTGGTTAAGAGTGGGGAGTCTAATCGATACGACCTGTCGTCTATTCGAGCCATGATAACTGGTGCCGCACCCATGGGAATGGAACTTGAAGAAGCTGTTAAGGCTAGGCTTCCAAAAACCACACTTGGACAAGGATACGGGATGACAGAGGCAGGGCCACTTTCCATTAGCCTGGCATTTGCAAAGGAGCCATTAGGGACAAAACCAGGTGCATGCGGGACTGTCGTAAGAAACGCAGAGATGAAAATAGTTGACACAGACACTGGTGCTTCCCTTCCTAAAAACAAACCCGGTGAAATTTGCATCAGAGGCACAAAGGTTATGAAAGGATACTTAAATGATCCGGAGGCCACAAAGAGAACTATAGACCAAGAGGGATGGCTACACACAGGTGATATAGGTTTCATTGACGATGATGATGAGCTTTTCATTGTTGATCGGTTAAAGGAGCTAATTAAATACAAAGGGTACCAAGTAGCTCCTGCAGAACTCGAAGCAATGTTGATTTCCCACCCAAACATTTCTGATGCTGCTGTTGTACCCATGAAAGACGAAGCTGCTGGAGAAGTCCCAGTTGCATTTGTTGTAAGATCAAATGGTTCTAAGATCACCGAGGATGAAATCAAGCAATACATTTCACAACAGGTGGTATTTTACAAAAGAATCAACAAAGTTTTCTTCACAGATACTATTCCTAAAGCAGCCTCAGGTAAAATTCTGCGAAAGGAATTAACTGCAAGACTTAGCGAAGGTCTGGTGTCCACTTAGGTTTCTGTCATAAGGACTGTCTCCTCCATATGCAAGTGCACCTGCAAGGAGATATGTATTTGAAAATTAGGTTGTTAACTCACGTAAGATCAAAGGCCCTTCTTATTTTGGCCTTCTATCGTAAATTTTATTAGCTATCTAAAGCTAAGATGTTAACATTTGTGACATAAAAAAAGGGATTTTAAAAACTCGATACAATATCAAGCTAATCCATACATGTTTCCAC

**CDS:**

KQTSPN-IKMSPAPTQEEFIFRSKLPNIPIPTHLPLHSYCFENLSKFHHRPCLINGDTGETLTYAEVHLTVRKIVAGLNSLNIHQGDVIMLVLRNCPQFALSFLGAAHRGAVITTANPFYTTTELAKQATATKSKLIITQSAYVEKIKHFAKTNDIKIVSIDSSTEEEDVLHFSVLTQANENDAPDVKINPDDVVALPFSSGTSGLPKGVMLTHKNLVTTIAQLVDGENPHQYTNCEDVLLCVLPMFHIYALNSILLCGIRSGAAVLIVQKFEITTLLELIQKYRVTVASFVPPIVLALVKSGESNRYDLSSIRAMITGAAPMGMELEEAVKARLPKTTLGQGYGMTEAGPLSISLAFAKEPLGTKPGACGTVVRNAEMKIVDTDTGASLPKNKPGEICIRGTKVMKGYLNDPEATKRTIDQEGWLHTGDIGFIDDDDELFIVDRLKELIKYKGYQVAPAELEAMLISHPNISDAAVVPMKDEAAGEVPVAFVVRSNGSKITEDEIKQYISQQVVFYKRINKVFFTDTIPKAASGKILRKELTARLSEGLVST-VSVIRTVSSICKCTCKEICI-KLGC-LT-DQRPFLFWPSIVNFISYLKLRC-HL-HKKRDFKNSIQYQANPYMFP

**>c780996_g1**

**Gene:**

GTAGCACACCCCTACAGCTCTTTTCTACAAGCAGTGTAATTAACTACCAATTGAGTTCAGATCTCAGTACATTAATTTGCTACCAACATATCTTCTTTGCTTAATATCAACAAGATGGTGAGTGTAGCTGAAATTCGTAAGGCTCAGAGGGCAGAAGGCCCTGCAACCATTTTGGCTATTGGTACCGCAAACCCATCAAACTGTGTTGAACAAAGCACTTATCCTGACTTTTACTTTAGAATCACAAACAGTGAACACAAGACTGAACTCAAAGAGAAATTTCAACGCATGTGTGATAAATCTATGATTAAGAGGCGATACATGTACCTAACAGAAGAGATTTTGAAAGAGAATCCTAATGTTTGCGAATACATGGCACCTTCTTTGGATGCTAGACAAGACATGGTGGTGGTAGAGGTACCTAGACTCGGGAAAGAGGCCGCAGTGAAAGCTATAAAAGAATGGGGGCAACCAAAGTCAAAGATTACACACTTAATCTTTTGTACCACGAGTGGTGTTGACATGCCTGGAGCGGATTACCAACTCACCAAACTCTTGGGTCTTCGCCCATATGTGAAAAGGTATATGATGTACCAACAAGGGTGCTTTGCAGGTGGAACGGTGCTTCGTTTGGCCAAAGACTTGGCAGAGAACAACAAAGGTGCTCGTGTGCTCGTTGTTTGTTCTGAAGTCACTGCAGTCACATTCCGTGGCCCAAGTGACACTCACTTGGACAGCCTGGTCGGACAAGCATTGTTTGGAGATGGAGCAGCAGCGGTCATTGTTGGTTCTGACCCCGTTCTTGAAATCGAGAAACCTATATTTGAGTTAGTTTGGACTGCACAAACAATTGCTCCGGATAGTGAAGGAGCTATTGATGGTCACCTACGTGAAGTTGGGCTAACATTTCATCTTCTTAAAGATGTTCCTGGGATTGTCTCAAAGAACATTGATAAAGCACTAGTGGAGGCATTCAAACCATTAGGAATCTCTGATTACAACTCAATCTTTTGGATTGCACACCCAGGAGGACCTGCAATTTTAGACCAAGTTGAGCAAAAGTTGGCCTTGAAACCTGAAAAGATGAGTGCCACTAGAGAGGTGCTTAGTGAATATGGTAATATGTCGAGTGCATGTGTCCTCTTCATCTTGGATGAGATGAGAAAGAAATCAGTTCAAGATGGACTTAAGACCACGGGTGAGGGGCTTGAATGGGGTGTGTTATTCGGCTTTGGACCTGGACTTACTATTGAAACAGTTGTTCTGCATAGTGTAGCTATATGAGATTCTTGATTGTTTTATATTCATGTATACTTTCAAACCCGTTTGAGTTTGTATCGAAGAAGAATAAACTCATCCAAAGTTCAATAATTTATATTTTCACATGTACTT

**CDS:**

VAHPYSSFLQAV-LTTN-VQISVH-FATNISSLLNINKMVSVAEIRKAQRAEGPATILAIGTANPSNCVEQSTYPDFYFRITNSEHKTELKEKFQRMCDKSMIKRRYMYLTEEILKENPNVCEYMAPSLDARQDMVVVEVPRLGKEAAVKAIKEWGQPKSKITHLIFCTTSGVDMPGADYQLTKLLGLRPYVKRYMMYQQGCFAGGTVLRLAKDLAENNKGARVLVVCSEVTAVTFRGPSDTHLDSLVGQALFGDGAAAVIVGSDPVLEIEKPIFELVWTAQTIAPDSEGAIDGHLREVGLTFHLLKDVPGIVSKNIDKALVEAFKPLGISDYNSIFWIAHPGGPAILDQVEQKLALKPEKMSATREVLSEYGNMSSACVLFILDEMRKKSVQDGLKTTGEGLEWGVLFGFGPGLTIETVVLHSVAI-DS-LFYIHVYFQTRLSLYRRRINSSKVQ-FIFSHVL

**>c828977_g1**

**Gene:**

AAACACATCTGATTCAAAGTGTAGTTATCTTCTTCCCAAGCCAACAACAACAACAAGATGGGCAGTGTTAGTGTTGAAAATATTCCTACAAAGGTTCTTACGAACAGCCCCTCTGGTGAAGTAAAGGTGCCTGTTATTGGCATGGGATCAGCCCCTGACTTCACCTGTAAGAAAGACACAAAAGAGGCCATCATTGAGGCCATCAAACAAGGTTACAGGCATTTTGACACTGCTGCTGCATATGGCTCTGAACAGGCTCTTGGAGAAGCTCTCAAGGAGGCAATCCACCTTGGCCTCGTCACCCGACAAGACCTCTTTGTCACTTCCAAGCTTTGGGTCACTGAAAATCATCCTCATCTCGTTCTTCCAGCTCTCCGCAAATCTCTCCAGACTCTTCAACTTGACTACTTGGACTTGTATCTCATCCACTGGCCCCTCACTTCTCAGCCTGGAAAGTTTTCATTCCCAATTCCTGTAGAGGACCTCTTGCCGTTTGATGTGAAGGGTGGTTGGGAATCCATGGAAGAATCCTTGAAACTTGGCCTCACTAAAGCTATTGGAGTCAGCAACTTCTCCGTCAAGAAGCTTCAAAGTCTGCTAGATGTTGCCACCATTCTTCCCGCAGTGAATCAAGTGGAGATGAATCTAGCATGGCAACAAAAGAAGCTTAGAGAGTTCTGCAATGCAAATGGAATAGTGTTAACAGCATTCTCACCATTGAGAAAAGGTGCTAGCAGGGGACCAAATGAAGTTATGGAGAACGATCTGCTTAAAGAGATTGCAGATGCTCATGGCAAGTCTATAGCACAGATCTCTTTGAGATGGTTGTATGAACAAGGAATCACATTTGTTCCCAAGAGCTATGATAAGGGGAGGATGAACCAAAATCTGCAAATCTTTGATTGGGCATTGACAAAGGAGGATCACCTCAAAATTGATCAAATCAAGCAGAGCCGTTTGATTGCAGGACCAACCAAGCCCAACCTCAATGATCTCTGGGATGACGAAATTTAAAATTAATATTCATTAATCTTTGCCTCGCGATCTATGATCTTTCTTTTTACACTTGTTGCTTCCTTTAGAGCTTTGTAGCTCTGTCTCTGTGTATCAAGTTTAATAATTTGTTTCCCAGTTCATCTCCCTAAAATTAATATTCATTAATCTTTGCCTCGCGACC

**CDS:**

KHIFKVLSSSQANNNNKMGSVSVENIPTKVLTNSPSGEVKVPVIGMGSAPDFTCKKDTKEAIIEAIKQGYRHFDTAAAYGSEQALGEALKEAIHLGLVTRQDLFVTSKLWVTENHPHLVLPALRKSLQTLQLDYLDLYLIHWPLTSQPGKFSFPIPVEDLLPFDVKGGWESMEESLKLGLTKAIGVSNFSVKKLQSLLDVATILPAVNQVEMNLAWQQKKLREFCNANGIVLTAFSPLRKGASRGPNEVMENDLLKEIADAHGKSIAQISLRWLYEQGITFVPKSYDKGRMNQNLQIFDWALTKEDHLKIDQIKQSRLIAGPTKPNLNDLWDDEINYSLIFASRSMIFLFTLVASFRALLCLCVSSLIICFPVHLPKINIHSLPRD

**>c801189_g1**

**Gene:**

ACGTCAAATCTTAAAGCAAAGACATTTAGATAAAAAATACTAGTCCCTCCTCCCATGGCAACAATAGCAACCTTAGCCGGAGTTAAAGTAGAGTTTCTTGAATTCCCACCAGTTGTCTCACCTCCAGGTTCCACCAAGTCCTATTTCCTCGGTGGAGCAGGTGTGAGAGGATTAGATATTAATGGGCAGTTCATAACTTTCACTGGTATCGGAGTTTACTTGGAAGAAAACGCCATAGCATCACTAGCTCCTAAGTGGAAGGGTAAGACTCCAGCTGAGCTCATACAATCCCTTGATTTCTACAGAGACATCATCAAAGGTCCCTTTGAGAAATTGGTGAGAGGGTCAAAGTTAAAAAAACTGGATGGTAATGAATATGTGAGGAAGGTATCAGAAAATTGTGCTAGCTTTATAAAATCTGAGGGAATACACAGTGAGGCTGAAGAAAAAGCTATTGAGGAATTTAGAGAAGCATTCAAGGATCGAGTTTTTCCACCAGGATCTACTGTTTTCTACAGACAATCACCTGCTGGAGAATTAGGACTTAGTTTCTCTAAAGATGAGACAGTACCAGAACATGAACATGCTGTTATAAATAACAAGGCACTTTCTGAGGCTGTGTTGGAGACTATGATTGGAGAGATTCCTGTTCCCCTGCTTTGAAAGAGAGCTTGGCTACACGGTTTTTTGAGCTTTTCAATGGGGTCAACTAATCCTAATAATGATATTTGAGATGATTTTTAAGGTGAGGTGGTGTGATCTTTGATTAAATAAGAGAATAATAATACGAGTGATATCTATGTTGATTAAATAAGAGAATAACTAGGCCAATCATTTCAAATAATTGCAATAAATCCACCATGCTTGTGTTGTTGCTGATATTTGAAGTGTTGTGAGATGGACTTGTGTATGATTTTAGTGCCATCTTCATCTTGCACCGGAGTCTCTTTTTCTTGGGCGGGTAGGACCATAATGAACGGCAAAACTCTCTCTCAAGAGTTTTAACGCTCGTGCATTTCCAAGGTTGGATGCAGTGGAGTCTCTATGGTTAGAGCTCCGGTGTCATTACACTTTACTTTTCTTGTAATATTACAAGAAAGTAAACAAAATTAAGATACCAAAAATTGACACCAATTTTGGTAGTCATTACAGTTTACCTACAAAGTTGATGAAGTGATGAACTCGAATAATAAATATATCAAGGTATTGCAAC

**CDS:**

TSNLKAKTFR-KILVPPPMATIATLAGVKVEFLEFPPVVSPPGSTKSYFLGGAGVRGLDINGQFITFTGIGVYLEENAIASLAPKWKGKTPAELIQSLDFYRDIIKGPFEKLVRGSKLKKLDGNEYVRKVSENCASFIKSEGIHSEAEEKAIEEFREAFKDRVFPPGSTVFYRQSPAGELGLSFSKDETVPEHEHAVINNKALSEAVLETMIGEIPVPLL-KRAWLHGFLSFSMGSTNPNNDI-DDF-GEVV-SLIK-ENNNTSDIYVD-IRE-LGQSFQIIAINPPCLCCC-YLKCCEMDLCMILVPSSSCTGVSFSWAGRTIMNGKTLSQEF-RSCISKVGCSGVSMVRAPVSLHFTFLVILQESKQN-DTKN-HQFW-SLQFTYKVDEVMNSNNKYIKVLQ

**>c759107_g1**

**Gene:**

AGGCAAAAACACAAAATCCTACTAGCTGTTTAGCCTCAACAGACTCGAGACTCTTACTTGTTCGTTCCTACTTCCTAGCAAGTTAATTAATTAATTTAGCAAAATCAAAGACGATGTTGTTAGAACTTGCAGTAACTCTATTGGTGATAGCTCTCTTCATACACCTTCGTCCCACACCTTCTGCAAAATCAAAGGCTCTTCGCCACCTTCCTAATCCTCCAAGTCCTAAACCTCGTCTCCCTTTCATTGGCCACCTTCACCTATTGGACAAACCTCTTCTTCATCAATCTCTCATCCGTCTAGGTGAACGCTATGGCCCTTTGTACTCTCTCTATTTTGGCTCCATGCCTTGCGTTGTTGCATCCACCCCTGAACTGTTCAAACTCTTTCTTCAAACCCATGAGGCTTCTTCCTTTAATACCAGGTTCCAAACCTCTGCTATTAGACGCCTCACCTATGATAACTCTGTTGCCATGGTTCCCTTTGGACCTTACTGGAGGTTCATTAGGAAGCTCATCATGAATGACCTTCTCAACGCCACCACTGTTAACAAGTTGAGGCCTTTGAGGAGCCAGGAAATCCGTAAGGTTCTTAATGTCATGGCAAAGAGTGCTGAGGCTCAGCAGCCCCTCAATGTTACCGAGGAGCTTCTCAAGTGGACCAATAGCACCATCTCTAGGATGATGTTGGGTGAAGCTGAAGAGATTAGAGATATTGCTCGTGATGTGCTTAAGATCTTTGGGGAGTATAGTCTTACAGACTTCATTTGGCCATTGAAGAAGTTCAAGGTTGGCCAGTATGAGAAGAGAATTGACGATATTTTCAACAGGTTTGATCCTGTGATTGAGAAGGTCATCAAGAAACGCCAAGAGATAATCAAGAGAAGAAAGGAGAGAAATGGAGAACTTGAAGAGGGTGAGCAGAGTGTAGTGTTTCTCGATACTTTGCTTCAATATGCTGAGGACGAGACGATGGAGATCAAAATTACCAAAGAACAAATCAAGGGTCTTGTTGTGGATTTCTTCTCTGCTGGAACGGATTCAACCGCCGTCGCAACAGATTATGCTTTGGCGGAGCTGATCAACAACCCCAAGGTCCTTAGAAAAGCACGAGAGGAGGTTGACACGGTTGTGGGAAAAGATAGACTGGTTGATGAATCAGATGTTCAACATCTTCATTACATTAGAGCTATTGTGAAGGAGACATTCCGTATGCACCCACCACTGCCCGTTGTGAAAAGAAAGTGTACACAAGACTGTGAGATCGACGGCTTTGTCATCCCAGAGGGAGCATTGATACTTTTCAATGTTTGGGCCGTTGGAAGAGATCCAAAGTACTGGGATAGGCCCTCAGAATTTCTTCCTGAGAGATTTTTAGAAAAAGCCGGTGGTGAAGGGGAAGTAGGTCCGATTGATCTTAGGGGTCAACATTTCCAACTTTTGCCATTTGGGTCTGGTAGGAGGATGTGCCCGGGAGTAAATTTGGCTACTGCTGGAATGGCCACGCTGCTTGCATCCGTTATCCAAACCTTTGATCTACAGGTACCAGGCCCACAAGGCCAAATATTAAAAGGAGATGAGGCTAAGGTTAGCATGGAAGAAAGAGCTGGTCTCACTGTTCCAAGGGCACATAATCTCATCTGTGTTCCGCTTGCAAGAGCAGGTGTCGCAGCTAAACTCCTTTCCTCTTGAAACCTGCAACAAAAAAGACAAGATAATGATGTCATGGAAGATGTTATTTTATAATTTATATATGTTTTGTAGTAATACTCATTTTCAATAAGGCGTCATTAATTAAGAGACAATGAGTCCAAGCCACGACACCCACAGGATGTTGTTGGAAGAGACACGTATATGCATAGTCTCAACTAGTCTCTCTTGTTATACAATGTACTTCTTTTTTAGTCACAAAATGAAAATTGTTGCAACTTCTATTTGTATTTTTAAGGGC

**CDS:**

AKTQNPTSCLASTDSRLLLVRSYFLASLINLAKSKTMLLELAVTLLVIALFIHLRPTPSAKSKALRHLPNPPSPKPRLPFIGHLHLLDKPLLHQSLIRLGERYGPLYSLYFGSMPCVVASTPELFKLFLQTHEASSFNTRFQTSAIRRLTYDNSVAMVPFGPYWRFIRKLIMNDLLNATTVNKLRPLRSQEIRKVLNVMAKSAEAQQPLNVTEELLKWTNSTISRMMLGEAEEIRDIARDVLKIFGEYSLTDFIWPLKKFKVGQYEKRIDDIFNRFDPVIEKVIKKRQEIIKRRKERNGELEEGEQSVVFLDTLLQYAEDETMEIKITKEQIKGLVVDFFSAGTDSTAVATDYALAELINNPKVLRKAREEVDTVVGKDRLVDESDVQHLHYIRAIVKETFRMHPPLPVVKRKCTQDCEIDGFVIPEGALILFNVWAVGRDPKYWDRPSEFLPERFLEKAGGEGEVGPIDLRGQHFQLLPFGSGRRMCPGVNLATAGMATLLASVIQTFDLQVPGPQGQILKGDEAKVSMEERAGLTVPRAHNLICVPLARAGVAAKLLSSNLQQKRQDNDVMEDVILFIYVLYSFSIRRHLRDNESKPRHPQDVVGRDTYMHSLNSLLLYNVLLFSQNENCCNFYLYFG

**>c773593_g1**

**Gene:**

AAGCAACTGAGTTGTAACATTTCACATAGCTATGGGCTCAAGGTATGTCCAAAAAGCAAATGACCTTTTTGAAGGCCAAACTCTCTTGTACGCACAAATTTTTGGTTACCTTAAAACTGTGTGTCTTAAGTGGGCTGTCCAACTAGGTATTCCAGACATAATAAAAATCATGGAGAATCCATTACCCTTCCTGAGTTACTATCAAAGCTGAAAGTTCCGCCATCTAAAACAAGTTGTGTTCCACGGTTAATGCGCTTTTTGGCACACAATAGAATCTTTGATATCCACGTTAACCAAAAAGGTCATCTATCATATTCTCTTACTCCTGCATCAGAACTTTTGGTGAGTAGCAGTGACCATTGTTTATCTCCCGTGGTTACTATGTTCACTAATCAAGTTCTGATGGGTGTTAACCATCACTTGGGAGAATGGGTTTGTGGGGAAGTTCCAACGCTATTTGAAGTAGCTTTAGGAACATCCTTTTGGGAGCTTGTTAAGGACAAACCTTCATATATGAATCTATTTAATGAGGGAATGGCAAGTGATTCTAAAATGGTTGACCTGGCTTTGAAAAATTATAGTTCTATATTTGAAGGGATTGATTCTATCGTGGATGTTGGTGGTGGAACTGGAACCACGGCAAAAATTATGTCTGCAAAATTTTCTAACCTCAAATGTATTGTGTTTGACCTTCCACATGTTGTCGCTAACTTGCTGGGAAGTGACAATTTGAGTTATGTTGGTGGGGACATGTTCATATCTATCCCCAAAGCTGATGCAGTTCTACTAAAGTGGATTTTACATGATTGGACCGATGAGAAATGCATAGAGATTTTGGAAAAGTGTAAATACTCAGTTTCAAGCAAAGAAAGTAAAGGAAAAGTGATCATCATAGATACCGTAATAATTGAAAAGGAAGACGATCAATATATGGCTGACACAAAACTTAGTATGGATATCTTTATGTTGGGTCTCAAAGGGAAAGAGCGAACTGAAAAAGAATGGAAACAACTCTTTATTGAAGCAGGATTTAAACACTACAAAATATTTCCCATCTTTGGTTTTAGATCTCTTATTGAGGTTTATCCGTAGACAATATCTATGACAGCTTGCCACGTCCTTATTATGTTAACCATTGTAGTGATGTATTTAATGCAATTAGCATATATGTTTTGAGCTGAATAAAGTTTTCAAG

**CDS:**

KQLSCNISHSYGLKVCPKSK-PF-RPNSLVRTNFWLP-NCVS-VGCPTRYSRHNKNHGESITLPELLSKLKVPPSKTSCVPRLMRFLAHNRIFDIHVNQKGHLSYSLTPASELLVSSSDHCLSPVVTMFTNQVLMGVNHHLGEWVCGEVPTLFEVALGTSFWELVKDKPSYMNLFNEGMASDSKMVDLALKNYSSIFEGIDSIVDVGGGTGTTAKIMSAKFSNLKCIVFDLPHVVANLLGSDNLSYVGGDMFISIPKADAVLLKWILHDWTDEKCIEILEKCKYSVSSKESKGKVIIIDTVIIEKEDDQYMADTKLSMDIFMLGLKGKERTEKEWKQLFIEAGFKHYKIFPIFGFRSLIEVYP-TISMTACHVLIMLTIVVMYLMQLAYMF-AE-SFQ

**>c792702_g1**

**Gene:**

CCATATCCCAAAACACACAAACACACATAACATAGGGGGAAAGGTTTAAAAGATGGCACCTTTATTGTATTACTCTCTTCTTTCTCTAGCTTTCATCCTAACCGTTAAAATTATCCTCCAAATTCAATCAAGAAGGCTAAAAAATCTTCCACCTGGTCCACCAACAATTCCAATAATAGGAAATCTCCACCATCTAAAACACCCTCTCCACCGCACCTTCACAACTCTATCTCAAAAATACGGCGACATCATTTCCCTGTGGTTCGGTTCACGTCTTGTCGTCGTTGTGGCTTCACCTTCCATAGCTCAGGAATGCTTCACTAAAAACGACGTCGTTTTAGCAAACAGGCCTAAATTCCTCACTGGAAAGTACATATTCTACAACTACACAACACTCGGCTCAGCTTCCTATGGCGACCACTGGCGTAACCTCCGCCGTATCACCACCATCGACGTCCTTTCCAACCACCGTCTCAATTCCTTCCATGGAGTCCGCAAAGACGAGACCTTGAGACTCGTGGAGAAGCTTCGTAACGACGTCGTTAAAGAAGGAAATTTCAGTTTCACTGATGTGGAATTGAGACATAGATTAACGGAGATGACGTTTAACGCAATGATGAGGATGATATCAGGGAAGAGATATTACGGAGATGACGGAGACGTGACGGATGTTAAAGAAGCGAAGCAATTTAGGGATATTATATCGGAAATATTGTCACTTTTAGGGGCTAATAATAAGGGTGATTTTTTGCCTTTGTTGAGATTGTTTGATCTTGATCACTTGGAGAAGAGGTGTAAGAGGATTTCAAAGAGAGCTGATGCGTTTTTACAGGGATTAATTGAACAACATCAAAATGCAAATCATAGTGATAATGATGGGGATACAATGATTGATCATCTTTTGAAGTTGAGGGAGACTCAGCCTGAGTACTATTCTGATCACATGATTAAAGGTCTTATCCAGGCTATGCTTCTTGCGGGGACAGACACATCAGCTGTGACAATAGAATGGGTGATGGCTGAATTGTTGAACAACCCAGAAGTATTGAAGAAAGCAAAAAAAGAAATTGAAACTACAATTGGAAAAGAGCGATTAGTAGAGGAGCAAGATTTGTCTCAGCTTCCGTACCTTCAAAACGTTATATCTGAGGCACTTCGGTTGCATCCTCCAGCTCCACTACTATTGCCTCATTCTGCTTCAGAAGATTGCACTATTGGAGGATTCAATGTCCCAAAAGACACTATAATATTAACTAATATTTGGGCCATTCATAGAGATCCTGAACTTTGGACTGACCCTTCAAGTTTTAAGCCCGAGAGATTTGAAAAAGAAGGAGAAGTAAACAAGTTACTTTCATTTGGGTTGGGAAGGAGGGCTTGTCCGGGCCTCAGTTTGGCCCAACGTACTGTGGGCTACACGGTGGGCTTACTGATTCAAGGCTTTGAATGGAAAACAGAAAGCGAGGAAAAACTTGATCTGGCAGAGGGCAAAGGAATCACCATGCCAATGAAGTTTCCGTTACGGGCCATGTGTAAACCACTGCCTATTGTCAATGACATTATGAAGTGATTAATTAATCTGAGGCTTCTTGAAGTTATGTGATGATAGAATAAAATGAAAGTGAGTTTTTGTTTTGTTTTTGTATCTAGTATAGCTGGGTACGGTTCTCAATTGCATAATAGTATCATACTACATTGTAAAATAAAGCATGAATGACTTATCTATGTATTTTCTACTTCAGTTGCTATCAATTACAGATAAATGATATTAGTTTTTACAAG

**CDS:**

HIPKHTNTHNIGGKVKMAPLLYYSLLSLAFILTVKIILQIQSRRLKNLPPGPPTIPIIGNLHHLKHPLHRTFTTLSQKYGDIISLWFGSRLVVVVASPSIAQECFTKNDVVLANRPKFLTGKYIFYNYTTLGSASYGDHWRNLRRITTIDVLSNHRLNSFHGVRKDETLRLVEKLRNDVVKEGNFSFTDVELRHRLTEMTFNAMMRMISGKRYYGDDGDVTDVKEAKQFRDIISEILSLLGANNKGDFLPLLRLFDLDHLEKRCKRISKRADAFLQGLIEQHQNANHSDNDGDTMIDHLLKLRETQPEYYSDHMIKGLIQAMLLAGTDTSAVTIEWVMAELLNNPEVLKKAKKEIETTIGKERLVEEQDLSQLPYLQNVISEALRLHPPAPLLLPHSASEDCTIGGFNVPKDTIILTNIWAIHRDPELWTDPSSFKPERFEKEGEVNKLLSFGLGRRACPGLSLAQRTVGYTVGLLIQGFEWKTESEEKLDLAEGKGITMPMKFPLRAMCKPLPIVNDIMKLINLRLLEVMNKMKVSFCFVFVSSIAGYGSQLHNSIILHCKIKHELIYVFSTSVAINYRMILVFT

**>c303354_g1**

**Gene:**

CTCGTAAAAAAAAATAATATGTCATTCATGACGTTTTTGCGTCAACCTTTTTACACCTGTCGTGGTCAGAAACAAACGTCTATGAGTTGCCATTCGTGAAATAAAATTATTATTAAATAGAAGTTGTGATTGAATTTGAATCTGCCACTTGCATCTCGATCCCTCATCACTAATTAAGCAAGTGAACATCTCCATTTTCCATTTCAACATGGAGTCCAAAACTGATTCAATCAAAATGTTCTTCTTCCCCTTTGTAGGTGGAGGTCATCAAATTCCAATGATAGACACTGCACGAGTATTCGCAGCACATGGAGCCATGTCAACTATCCTAACCACACCCTCCAACGCCCTCCATTTCCAAAAATCAATCACTCGCGACCAGCAATCATGTCTTCCCATCACCATCCACCTCCTCACCACCACCGTAGACATAACCGACACAGACATGTCAGCTGGTCCCATGATCGACACCTCGATTCTCCTCGAACCTCTGAGAGAGTTCCTACTTCAACACCCACCCGATTGCATAGTGGTTGACATGTTTCATCGTTGGGCCAACGATGTTATTGACGAACTCAAAATCCCAAGAATTTTCTTCACTGGTAACGGATGCTTCCCTCGTTGTGTCCATGAAAATATTTCAAGACATGCTGTCCTTGACAATCTAAGTTCAGATTCAGAACCTTTTATTGTCCCTGGTCTTCCCGACAAAATCGAAATGACGAGGTCTCAGCTTCCGATTTTCGCGAGAAACCCGTCTCAGTTTCCTGATAGGCTGAGGCAAATGGAGGGCAAGAGTTTTGGCACCGTCATCAATAGCTTCTATGACTTGGAACCAGCTTATGCTGATTACATCATAAACGTGTTGGGAAAGAAAGCATGGCTTGTAGGACCAGTTTCCCTTTGCAACATAAGCGTAAAAGATAAAACAGAGAGAGGGAAGCAACCCACAGTAGATGAACAAAGTTGCTTGAATTGGTTGAATTCAAAGAAACCCAATTCAGTTATTTATGTCAGTTTTGGAAGCTTGGCTCGCTTACCCCCTGAGCAACTCTGTGAAATTGCTTATGGCCTCGAAGCTTCTGAGCAATCTTTCATTTGGGTAGTGGGAAAGGTTCTCAACTCTTCCAAAAAAGAAGAAGTTGGCGGTGATCAAAATTGGCTTCCTAATGGGTTTGAAGAGAGGATGAAAGAAACGAATAAAGGATTGATCCTAAGGGGTTGGGCTCCTCAGTTGTTGATACTGGAGCATGCTGCAGTTGGAGGGTTTGTGACACACTGTGGATGGAACTCGACTTTAGAAGGAGTGTGTGCAGGAGTGCCGATGGTTACGTGGCCACTAACGGCTGAGCAATTTTCAAATGAGAAGTTGATAACGAATGTGTTGGGGATTGGGGTCCAAGTGGGTAGCAGAGAGTGGTGGTCATGGAATGGAGAATGGAAACAAGTTGTGGGGAGAGAGAGGGTGGAGATGGCGGTGAAGAAGCTAATGACACGGAATGAAGAGGCAGAGGAGATGAGAAGGCGAGTCAAACACATTGCAGGGAATGCGAGAAGAGCTATCAAAGAAGGTGGAACTTCTTATGCTGATATCGATGCCTTAATTCAGGAGCTAAAAGCTAACAGATTCACAAGCCAAGTTTAGATTAGTAAAACCATTGGAAAAAATACTTCATCCTTCCAATTTTAAAACGTTAATATTACTGGCTTTCTTTTTTTAGTTTGTGTATGTCTGTTAAAAAAACTTCGTGACCCATTATGGTGCTGTCAGTGAACAAACTCAGCAAGCTTGCAGGCTGAAATTACCAGTGCGAACTTCTATTACTCCACGTGTCGTAATTGTTCCCATTCATGGCCGGAGTAACGAGAATCGCCCCTGGGTTCTCGGCCGGATGAAGGTAGTAGGGGCTCGTGACGATGGACGCAGGGTCTTTGGGTTCGCCATTGTTCTTTTGTGTGTGTTCTATCTCGGCGGAATCATCGGAGTGAACGACTGGTTCTGTCATGGTGGAATGTGAGAAGAGGGTGGATCGAAACGCAGAGTGGGTTACTCCGCTCCTGATACCATAAAAGTGAAATTCACAAAAATGTGAAAATGTTTCTCATTTGTTCAATTGAAGTTCTGAAAGAGTACATATATATGATGATTCTGATAAAGAATCTGTTATACTAACTAGTACACCCGTGGGGATGTATTATACACCCGCGTGGGTGTATAGAAGGGCCAAAAGTGAGACTTTATACAAAGGTAATCATAGTAAACTAATATAGAGTTTTGTGTATTCCAAT

**CDS:**

S-KKIICHS-RFCVNLFTPVVVRNKRL-VAIREIKLLLNRSCD-I-ICHLHLDPSSLIKQVNISIFHFNMESKTDSIKMFFFPFVGGGHQIPMIDTARVFAAHGAMSTILTTPSNALHFQKSITRDQQSCLPITIHLLTTTVDITDTDMSAGPMIDTSILLEPLREFLLQHPPDCIVVDMFHRWANDVIDELKIPRIFFTGNGCFPRCVHENISRHAVLDNLSSDSEPFIVPGLPDKIEMTRSQLPIFARNPSQFPDRLRQMEGKSFGTVINSFYDLEPAYADYIINVLGKKAWLVGPVSLCNISVKDKTERGKQPTVDEQSCLNWLNSKKPNSVIYVSFGSLARLPPEQLCEIAYGLEASEQSFIWVVGKVLNSSKKEEVGGDQNWLPNGFEERMKETNKGLILRGWAPQLLILEHAAVGGFVTHCGWNSTLEGVCAGVPMVTWPLTAEQFSNEKLITNVLGIGVQVGSREWWSWNGEWKQVVGRERVEMAVKKLMTRNEEAEEMRRRVKHIAGNARRAIKEGGTSYADIDALIQELKANRFTSQV-ISKTIGKNTSSFQF-NVNITGFLFLVCVCLLKKLRDPLWCCQ-TNSASLQAEITSANFYYSTCRNCSHSWPE-RESPLGSRPDEGSRGS-RWTQGLWVRHCSFVCVLSRRNHRSERLVLSWWNVRRGWIETQSGLLRS-YHKSEIHKNVKMFLICSIEVLKEYIYMMILIKNLLY-LVHPWGCIIHPRGCIEGPKVRLYTKVIIVN-YRVLCIP

**>c778119_g1**

**Gene:**

GTTCATTCATTCATTCATTCATTCAAACAGATGGCGTGCAACAAAACACCTCAACTCCACTTCGTTTTATTTCCACTAATGTCACCAGGCCACATGCTTCCAATGATAGACCTAGCAACAACATTAGCATCACAACAAAACATAATTGTAACCGTAATAACAACTCCACACAACGCATCACGTTTCTCACAAACCTTCTCACGTGCTTCCAATTCCAACCGTCAACTCCATTTACTCCAACTCCAATTCCCATCTAATGATTTTGGATTCCCACGAGGTTGTGAGAATTTCGATATGCTTCCTTCAATGGGGATGGGACACAGTTTCTTCATAGCAGCAAATGCAATTCTACAAGAACCAGCTGAAAAGGCTTTTGAGAAGTTAACTCCAAAACCAAATTGCATTATCTCTGATGTTAGTTTCCCTTACACTTCTCATATTGCTATAAAATTCAACATTCCTAGAATTTCATTTTACGGTGTTAGTTGCTTTTGTCTTGTTTGGCAACAAAATTTGATTATTTCAAAAGTTATGGAGAATATAGATACCGATTCAGAGTACTTTGTTATACCTGAAATTCCGGATAAAATTGAGATTACTAAAGCACAGATCCCATCTTCTTCAAATTCAATGGATGAAAAATGGAAGGAGTTTGTTGATATAATGGTTGCAGCTGAAATGGTTAGTTATGGAGTGGTAGTGAATTCTTTTGAGGAGTTAGAGTTTGCTTATGCTAGTGATTTCAAGAAGGGTAAAAATGGCAAAGTGTGGTGTGTTGGTCCTCTTTCACTTAGAAATAGAAGCCACGTGGATATCGCTGAGCGAGGGAACAACAACAACAACAAGGACACAAATTCAGTTGATGTTGAACAGTGGTTGGAGTTGCAAAAACCAAATTCTGTTATTTATGTATGTCTTGGGAGCATGTGTAATTTAACACCTATGCAGTTTATTGAGCTTGGTTTGGCCTTAGATGAATGTGAGAGGCCATTCATTTGGGTTATAAGGGAAAGAAATCAAACTGAAGAATTGAATAAGTGGATAAAGGAATATGGTTTTGAGGAAAGGACTAAAGGGAGAGGACTATTGATTAAGGGTTGGGCACCTCAAGTGTTGATACTATCAAATCCTGCAATTGGAGGGTTTATAACACATTGTGGTTGGAACTCTAGTTTAGAAGCAATATGTGCTGGGGTGTCAATGCTTACGTGGCCATTGTTTGGTGATCAGTTTTTTAATGAGAGGTTTGTTGTGGAAATATTAAGAGTTGGAGTGAGTGTTGGTGTGGAGAGTCCTGTAAATTGGGGTGATGAAGAGAGAGCTGGAGTGTTGGTTACGAAAGAAGATGTTGTGAGAGGTATTGAGAAGTTGATTAGTGAGGGAAGTGATTGTGATGAGAGGAGAAAAAGGGCTAAAGAATTTGCTAACATGGCTAAGAGAGCGGTAGAAGAAGGAGGATCTTCTCACTTCAATGTGAAGCTACTAATCCAAGATATTGCACAATAGTCCAACAATGAGTATTTTGATGTTGTTCATAATTGATTCTGTAACTTTGTCCAACATCAACAACTAGTTGTTGTTTCGCACAACTTGTTTTTTGTTACTTGTTACTAAGTACACATGTTTCTTCAAATCGAGTATTTTGAG

**CDS:**

VHSFIHSFKQMACNKTPQLHFVLFPLMSPGHMLPMIDLATTLASQQNIIVTVITTPHNASRFSQTFSRASNSNRQLHLLQLQFPSNDFGFPRGCENFDMLPSMGMGHSFFIAANAILQEPAEKAFEKLTPKPNCIISDVSFPYTSHIAIKFNIPRISFYGVSCFCLVWQQNLIISKVMENIDTDSEYFVIPEIPDKIEITKAQIPSSSNSMDEKWKEFVDIMVAAEMVSYGVVVNSFEELEFAYASDFKKGKNGKVWCVGPLSLRNRSHVDIAERGNNNNNKDTNSVDVEQWLELQKPNSVIYVCLGSMCNLTPMQFIELGLALDECERPFIWVIRERNQTEELNKWIKEYGFEERTKGRGLLIKGWAPQVLILSNPAIGGFITHCGWNSSLEAICAGVSMLTWPLFGDQFFNERFVVEILRVGVSVGVESPVNWGDEERAGVLVTKEDVVRGIEKLISEGSDCDERRKRAKEFANMAKRAVEEGGSSHFNVKLLIQDIAQ-SNNEYFDVVHN-FCNFVQHQQLVVVSHNLFFVTCY-VHMFLQIEYFE
